# Utility of in silico-identified-peptides in spike-S1 domain and nucleocapsid of SARS-CoV-2 for antibody detection in COVID-19 patients and antibody production

**DOI:** 10.1038/s41598-022-18517-w

**Published:** 2022-09-05

**Authors:** Karen Cortés-Sarabia, Víctor Manuel Luna-Pineda, Hugo Alberto Rodríguez-Ruiz, Marco Antonio Leyva-Vázquez, Daniel Hernández-Sotelo, Fredy Omar Beltrán-Anaya, Amalia Vences-Velázquez, Oscar Del Moral-Hernández, Berenice Illades-Aguiar

**Affiliations:** 1 Laboratorio de Inmunobiología y Diagnóstico Molecular, Facultad de Ciencias Químico Biológicas, Universidad Autónoma de Guerrero, Chilpancingo de los Bravo, Guerrero Mexico; 2 Unidad de Investigación en Inmunología y Proteómica, Laboratorio de Investigación en COVID-19, Hospital Infantil de México “Federico Gómez”, Mexico City, Mexico; 3 Laboratorio de Biomedicina Molecular, Facultad de Ciencias Químico Biológicas, Universidad Autónoma de Guerrero, Chilpancingo de los Bravo, Guerrero Mexico; 4 Laboratorio de Epigenética del Cáncer, Facultad de Ciencias Químico-Biológicas, Universidad Autónoma de Guerrero, Chilpancingo de los Bravo, Guerrero Mexico; 5 Laboratorio de Virología, Facultad de Ciencias Químico Biológicas, Universidad Autónoma de Guerrero, Chilpancingo de los Bravo, Guerrero Mexico

**Keywords:** Viral infection, Applied immunology

## Abstract

SARS-CoV-2 contains four structural proteins, two of which, the spike and nucleocapsid, are commonly used for the standardization of novel methods for antibody detection; however, some limitations in their use have been observed due to the homology of this virus with other phylogenetically-related viruses. We performed in silico analysis to search for novel immunogenic and antigenic peptides. A total of twenty-five peptides were preliminarily selected, located in the 3D structure of both proteins. Finally, eight peptides were selected: one located in the N protein and seven in the S1 domain of the spike protein. Additionally, the localization of selected peptides in 2D structures and possible changes in the sequences of these peptides in SARS-CoV-2 variants of concern were analyzed. All peptides were synthetized in MAP8 format, and recombinant S (trimer and RBD) and N proteins were used as antigens to search for antibodies in serum samples derived from COVID-19 patients, and for antibody response in New Zealand rabbits. Results showed high recognition of the serum derived from COVID-19 patients to all selected peptides; however, only the RBD3 peptide induced antibody production. In conclusion, this work provides evidence for a new strategy in peptide selection and its use for antibody detection or antibody production in animals.

## Introduction

COVID-19 disease is currently the primary public health issue worldwide, and it is caused by infection with severe acute respiratory syndrome coronavirus type 2 (SARS-CoV-2). The first cases were reported in the city of Wuhan in the province of Hubei, China, in a seafood wholesale market^1^, and as of July 18, 2022, a total of 559,469,605 confirmed cases, 12,130,881,147 vaccine doses and over 6.3 million deaths had been reported worldwide (https://covid19.who.int/). The primary symptoms associated with COVID-19 are fever, cough, myalgia, fatigue, dyspnea, sore throat, runny nose and headache, and less common symptoms include diarrhea, nausea, and vomiting. Patients with comorbidities such as diabetes, hypertension, cardiovascular disease, or chronic obstructive pulmonary disease have a higher risk of developing clinical complications in response to COVID-19, such as pneumonia, acute respiratory distress syndrome (ARD) and acute cardiac injury^2^.

SARS-CoV-2 is an enveloped, positive single-stranded RNA (+ ssRNA) of approximately 30 kb, with 14 open reading frames (ORFs) that encode 27 proteins^3,4^. The four structural proteins spike (S), envelopment (E), matrix (M), and nucleocapsid (N) are widely used for the development of diagnostic methods and vaccines, with the S and N proteins being the primary targets. The S protein mediates entry into host cells by interacting with the angiotensin-converting enzyme 2 (ACE2) receptor, and the N protein forms the viral capsid and interacts with RNA^5^. SARS-CoV-2 variants are classified according to their lineage and component mutations, on 31 May 2021, the WHO designated as variants of concerns (VOC), those variants that spread widely and display evidence for being more transmissible, causing more severe disease or reducing neutralization by antibodies generated during previous infection or vaccination. The current VOCs were named based on the Greek alphabet as Alpha (B.1.1.7), Beta (B.1.351), Gamma (P.1), Delta (B1.617.2) and the newly described Omicron (B.1.1.529)^6–8^.

Several tests have been developed for the diagnosis of SARS-CoV-2 infection^9^. Although quantitative reverse transcription polymerase chain reaction (RT–qPCR) is currently considered the “gold standard” method for diagnosis^10,11^, additional or alternative tests such as antibody and antigen detection are widely used as complementary tests^12^. The use of serological tests to evaluate the presence of IgM or IgG antibody production in response to SARS-CoV-2 infection or vaccination has many advantages for diagnosis (less invasive because only blood samples are needed, faster and cheaper)^13^. To which, many studies have focused their efforts on determining the best strategy for the selection of immunogenic or antigenic sites in SARS-CoV-2 using immunoinformatic tools as IEDB, NetCTL server, BCPred, VaxiJen server, ProtParam, among other, for the design of novel methods for antibody detection or vaccines. The main objective of in silico analysis is to identify immunogenic peptides derived from conserved and highly networked regions with topological importance (exposition on the surface) from the S and N protein that can promote the activation of B and T-cells to elicit immune response in the host system^14–18^.

The use of different bioinformatics tools have improved the search of immunogenic peptides^19,20^; however, in silico analysis to identify novel epitopes that could be used for the development of antibody-based diagnostic tests or the production of monoclonal or polyclonal antibodies for the development of antigen-based diagnostic tests using immunogenic and antigenic peptides continues. Thus, the objective of this study was to search for and characterize epitopes in the S and N proteins of SARS-CoV-2 that could be used for antibody detection in serum samples derived from COVID-19 patients using indirect ELISA and immune response induction in immunized animals for antibody production that could be used for diagnostic purposes.

## Results

### Identification of immunogenic and antigenic peptides using in silico analysis

Using the IEDB analysis resource, we searched for the presence of linear epitopes, sequential epitopes, surface accessibility and antigenicity; complementary analyses were performed in BepiPred-2.0 to predict antigenic peptides. The raw material of all peptides is shown in Supplementary Material S1, whereas in Table 1, a concentrated number of peptides found in each part of the S (S1 and S2) and N proteins is shown. During the first two rounds of selection, we observed that most of the peptides in the S protein were in the S2 domain. Furthermore, 59% were linear epitopes, 42% were sequential epitopes, 55% had surface accessibility and 52%-65% exhibited antigenicity, while the number of peptides found in the S1 NTD and S1 RBD was between 15 and 32% in each category. The S protein is highly antigenic; however, some peptides were shorter than eight amino acids, and during the second round of selection, they were eliminated: eleven in S1 NTD, five in S1 RBD and twenty-six in S2. With respect to the N protein, twelve linear epitopes and sequential epitopes were identified, ten with surface accessibility, fifteen with antigenicity and seven B cell epitopes, with no changes between the first and second rounds of selection (Table 1). For the third round of selection, only S1 (without subclassifications) and S2 domains were used considering immunogenicity, antigenicity, and the type of epitope (linear or conformational) for the selection of fifteen peptides in S1, four peptides in S2 and six peptides in N (Table 2).

**Table 1:** Number of immunogenic and antigenic peptides localized in each type of software used.

SARS-CoV-2 Proteins	IEDB analysis resource				BepiPred2.0	PAP
	Linear epitopes	Sequential epitopes	Surface accessibility	Antigenicity		
**First round of selection**						
**Protein S**						
S1 NTD	3 (18)	8 (26)	8 (28)	15 (33)	7 (30)	9 (19)
S1 RBD	4 (24)	10 (32)	5 (17)	7 (15)	6 (26)	8 (17)
S2	10 (59)	13 (42)	16 (55)	24 (52)	10 (43)	31 (65)
Total	17 (100)	31 (100)	29 (100)	46 (100)	23 (100)	48 (100)
**Protein N**	12 (100)	12 (100)	10 (100)	15 (100)	7 (100)	7 (100)
**Second round of selection**						
**Protein S**						
S1 NTD	3 (18)	8 (27)	3 (23)	4 (24)	7 (30)	9 (19)
S1 RBD	4 (24)	9 (30)	2 (15)	7 (41)	6 (26)	7 (15)
S2	10 (59)	13 (43)	8 (62)	6 (35)	10 (43)	31 (66)
Total	17 (100)	30 (100)	13 (100)	17 (100)	23 (100)	47 (100)
**Protein N**	12 (100)	12 (100)	10 (100)	15 (100)	7 (100)	7 (100)

*PAP* predicted antigenic peptides.

**Table 2:** Selected immunogenic and antigenic peptides in the S and N proteins of SARS-CoV-2.

Epitope	Protein	Domain	Epitope sequence*	Epitope length	Position
1	S	S1	SQCVNLTT	8	13–20
2			**RTQLPPAYTNS**	11	21–31
3			GVYYPDKVF	9	35–43
4			SNVTWFHAIHVSGTNGTKRF	20	60–79
5			FLGVYYHKNNKSWME	15	140–154
6			**MESEFRVYSSANN**	13	153–165
7			INLVRDLPQGFSA	13	210–222
8			LTPGDSSSGWTAG	13	249–261
9			VRQIAPGQTGKIAD	14	407–420
10			**SNNLDSKVGGNYNYLY**	15	438–453
11			**RLFRKSNLKPFERD**	14	454–467
12			**ISTEIYQAGSTPCNGVEGF**	19	468–486
13			**YGFQPTNGVGYQ**	12	495–506
14			**GPKKSTNLVKNK**	12	526–537
15			RDIADTTDAVRDPQ	14	567–580
16	S	S2	QTQTNSPRRARSV	13	675–687
17			ILPDPSKPSKRS	12	805–816
18			VPAQEKNFTT	10	1068–1077
19			FDEDDSEPVL	10	1256–1265
20	N		**MSDNGPQNQRNAPRIT**	16	1–16
21			AALALLLLDRL	11	217–227
22			KHWPQIAQFAPSASAFF	17	299–315
23			RIRGGDGKMKDL	12	93–104
24			SPRWYFYYLG	10	105–114
25			ILLNKHIDAYKT	12	351–362

*In bold: peptides selected for MAP8 synthesis.

### Localization of selected peptides in tertiary structures

**Figure 1:**
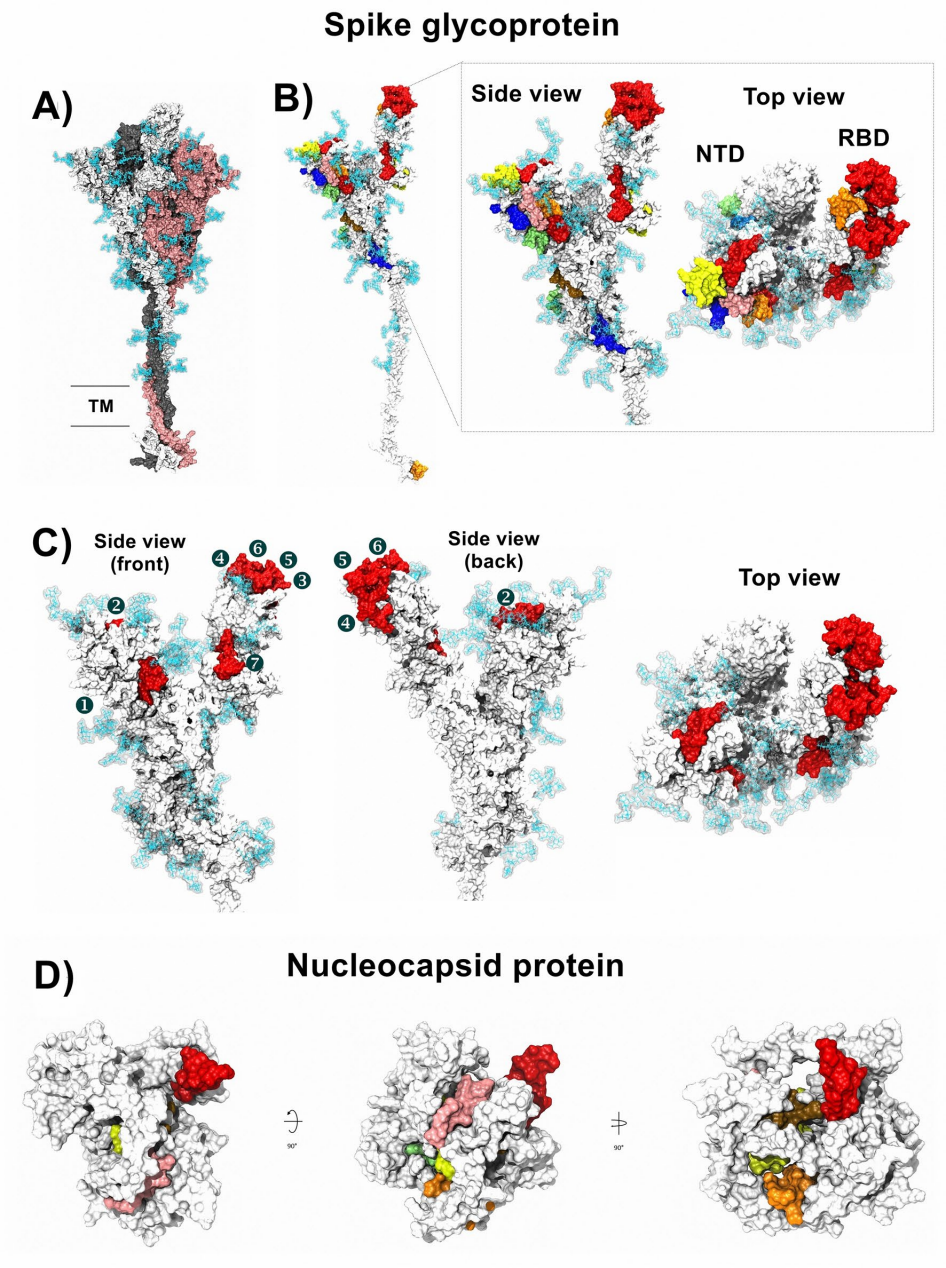
Localization of immunogenic and antigenic peptides in the 3D structures of SARS-CoV-2 S and N proteins. (**A**) The structure of the glycosylated trimeric S protein is shown in a side view. (**B**) Localization of all selected peptides in the monomeric glycosylated form of S protein. In the frame, the zoom of the side and top views of the S1 domain in the monomeric form of S protein shows the localization of each selected peptide in color patterns (see Supplementary Material S2). (**C**) Localization of the seven selected peptides (red) for the synthesis of MAP8 format is shown with numbers in side view (front and back) and top view. Glycans are represented as cyan lines of surf structures, and the transmembrane viral region is represented with two lines in gray (TM). (**D**) General view of a model structure of nucleocapsid protein. The color pattern used for each immunogenic and antigenic peptide located in the N protein is shown in Supplementary Material S2. Selected peptide is show in red.

To analyze the localization of each peptide in the 3D structure, first, the trimeric structure of the glycosylated spike protein was constructed (Fig. 1A), and the localization of all preselected peptides was determined in the monomeric structure. Most of the peptides were exposed, and none of them were in glycosylation sites (Fig. 1B). The localization of each peptide and the color pattern used are shown in Supplementary Material S2. For the final selection step, we performed conservancy analysis (result of 100%) and protein BLAST using sequences derived from SARS-CoV-2 (Supplementary Material S3), and complementary conservancy analysis were performed considering other Coronavirus-related virus that infect human and animals (Supplementary materials S4 and S5), also, we considered the exposition in 3D structure. We selected seven peptides in the S1 domain, two in the S1 NTD from amino acids 13 to 20 (RTQLPPAYTNS) and 153 to 165 (MESEFRVYSSANN), while the selected peptides in the S1 RBD were located from amino acids 438 to 537. All selected peptides are marked in red in their monomeric structure (Fig. 1C). Additionally, all preselected and selected peptides were marked in the trimeric structure of the spike protein, and similar results were observed (Supplementary Material S6). Later, we analyzed the localization of preselected and selected peptides in the 3D structure of the N protein. Unlike the S protein, most of the peptides in the N protein were found internally, and only the selected peptide was fully exposed and located from amino acids 1 to 16 (MSDNGPQNQRNAPRIT) (Fig. 1D).

### Characterization of selected peptides in the secondary structure and SARS-CoV-2 VOC

**Figure 2:**
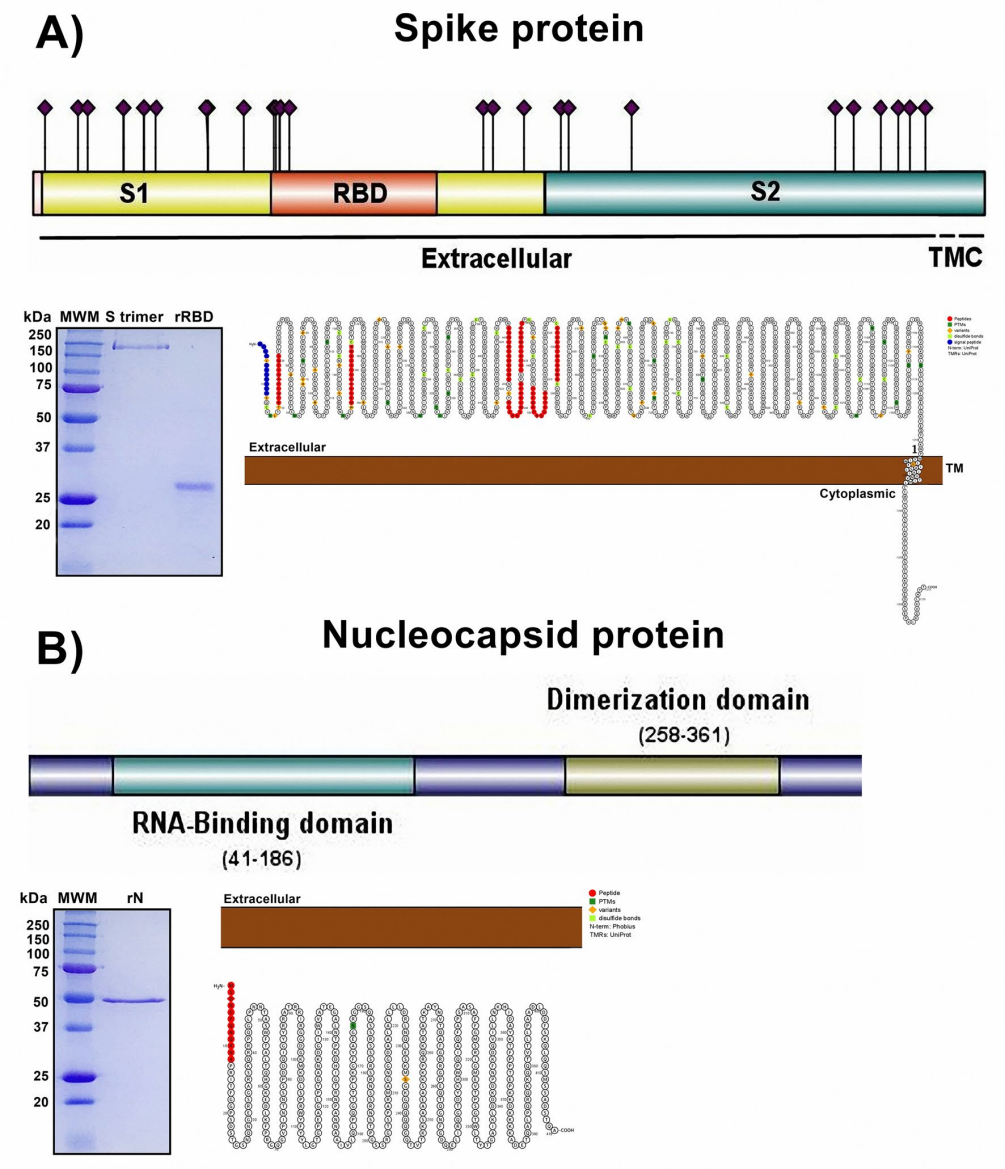
Recombinant proteins of the spike glycoprotein and nucleocapsid of SARS-CoV-2 were obtained, and the primary and secondary structures were analyzed. (**A**) Spike protein. Upper panel and lower right panel, analysis of the primary and secondary amino acid sequences showing the extracellular, TM (transmembrane) and cytoplasmic (**C**) localization of each amino acid. Purple diamonds represent glycosylated residues. (**B**) N protein. Upper panel and lower right panel showing analysis of the primary and secondary amino acid sequences with intraviron localization, phosphorylated amino acids, RNA-binding and dimerization domains. In both proteins, selected peptides are shown in red in 2D structure, and the peptide signal is marked in blue. Middle panel in (**A**) and (**B**): electrophoresis and Coomassie blue staining of recombinant RBD, S trimer, or N. *MWM* molecular weight marker.

We analyzed the primary amino acid sequence of both proteins and localized specific domains as receptor binding domains or posttranscriptional modifications (PTMs) such as glycosylation in the spike protein and RNA-binding or dimerization domains in the nucleocapsid protein. Interestingly, analysis of the selected peptides in protein plots or snake diagrams provided information about the localization of selected peptides (extra, transmembrane, or intraviron) in the spike or N protein, as well as PTM, disulfide bonds and signal peptide. During the chemical synthesis of selected peptides, we obtained the recombinant proteins of trimeric spike (S trimer), recombinant RBD (rRBD) and recombinant N (rN), and once the purification procedure was completed, electrophoresis and Coomassie blue staining were performed. The S trimer and rRBD possessed approximate molecular weights (MWs) of 250 kDa and 25 kDa, respectively, whereas rN had an approximate MW of 50 kDa (Fig. 2) (Supplementary Material S7). Recombinant proteins were quantified by Bradford assay and stored at 20 °C until use. We analyzed the effect of amino acid changes (obtained from the database of CoVariants) found in the VOCs (Supplementary Material S8) and their localization in all selected peptides. We observed a change of proline for serine in position 26 (P26S) in the sequence of NT-1 of the Gamma variant. NT-2 contains an amino acid change of arginine to glycine position 158 (R158G), and a deletion in position 156–157 in the Delta variant. Furthermore, two amino acid changes in the peptides RBD1 (N440K and G446S) and RBD3 (S477N and T478K), were are also observed in the Delta variant. The sequence of RBD4 peptide, contains a key amino acid change, N501Y, associated with VOCs Alpha, Beta, Gamma and Omicron. The Omicron variant also contains another three amino acid changes; G496S, Q498R and Y505H in the RBD4 peptide sequence. The peptides RBD2 and RBD5 do not contain any amino acid changes or deletions, whereas, the sequence of N peptide presents a single amino acid change in the Alpha variant (D3L) (Table 3).

**Table 3:** Amino acid changes and deletions in SARS-CoV-2 variants of concern and their localization in the selected peptides.

Peptides	Localization	Alpha (B.1.1.7)	Beta (B.1.351)	Gamma (P.1)	Delta (B.1.617.2)	Omicron (BA.1)
**NT-1** RTQLPPAYTNS	21–31	–	–	P26S	–	–
**NT-2** MESEFRVYSSANN	152–164	–	–	–	R158G Δ156–157	–
**RBD1** SNNLDSKVGGNY	438–450	–	–	–	N440K G446S	–
**RBD2** RLFRKSNLKPFE	454–465	–	–	–	–	–
**RBD3** ISTEIYQAGST	468–478	–	–	–	S477N T478K	–
**RBD4** YGFQPTNGVGYQ	495–506	N501Y	N501Y	N501Y	–	G496S Q498R N501Y Y505H
**RBD5** GPKKSTNLVKNK	526–537	–	–	–	–	–
**N** MSDNGPQNQRNA	1–12	D3L	–	–	–	–

*Amino acid changes and deletions of each variants were collected from the database CoVariants (https://covariants.org/variants). Δ: Deletion.

The eight selected peptides were synthetized in MAP8 format, provided that information regarding the synthesis included HPLC and MS. The length of the peptides was approximately 11–12 amino acids each with a molecular weight (MW) > 10 kDa due to the eight ramifications of the multiantigenic peptide. The predicted molecular weight correlated with the observed MW in MS, and HPLC results showed that MAP8 peptides had an estimated purity of 50–78% (Table 4). Crude data from the MS and HPLC are included in Supplementary Material S9. After synthesis was complete, MAP8 peptides were hydrated in sterile PBS to a final concentration of 1 mg/mL and used as antigens for antibody detection in COVID-19 patients and immune response induction in New Zealand rabbits.

**Table 4:** Characteristics of MAP8 peptides.

Name	Sequence	Length	Predicted MW	MW^a^	Purity
NT-1	RTQLPPAYTNS	11	10.7	10.7	57.3%
NT-2	MESEFRVYSSAN	12	12.1	12.1	NP
RBD1	SNNLDSKVGGNY	12	10.9	10.9	74.8%
RBD2	RLFRKSNLKPFE	12	13	13	65%
RBD3	ISTEIYQAGST	11	10.1	10.1	63.6%
RBD4	YGFQPTNGVGYQ	12	11.4	11.4	68.3%
RBD5	GPKKSTNLVKNK	12	11.3	11.3	60.2%
N	MSDNGPQNQRNA	12	11.4	11.4	78.5%

*MW* molecular weight provided in kDa, *NP* not provided.

^a^Observed molecular weight in MS.

### Utility of MAP8 peptides and recombinant proteins for antibody detection in healthy and COVID-19 patients

**Figure 3:**
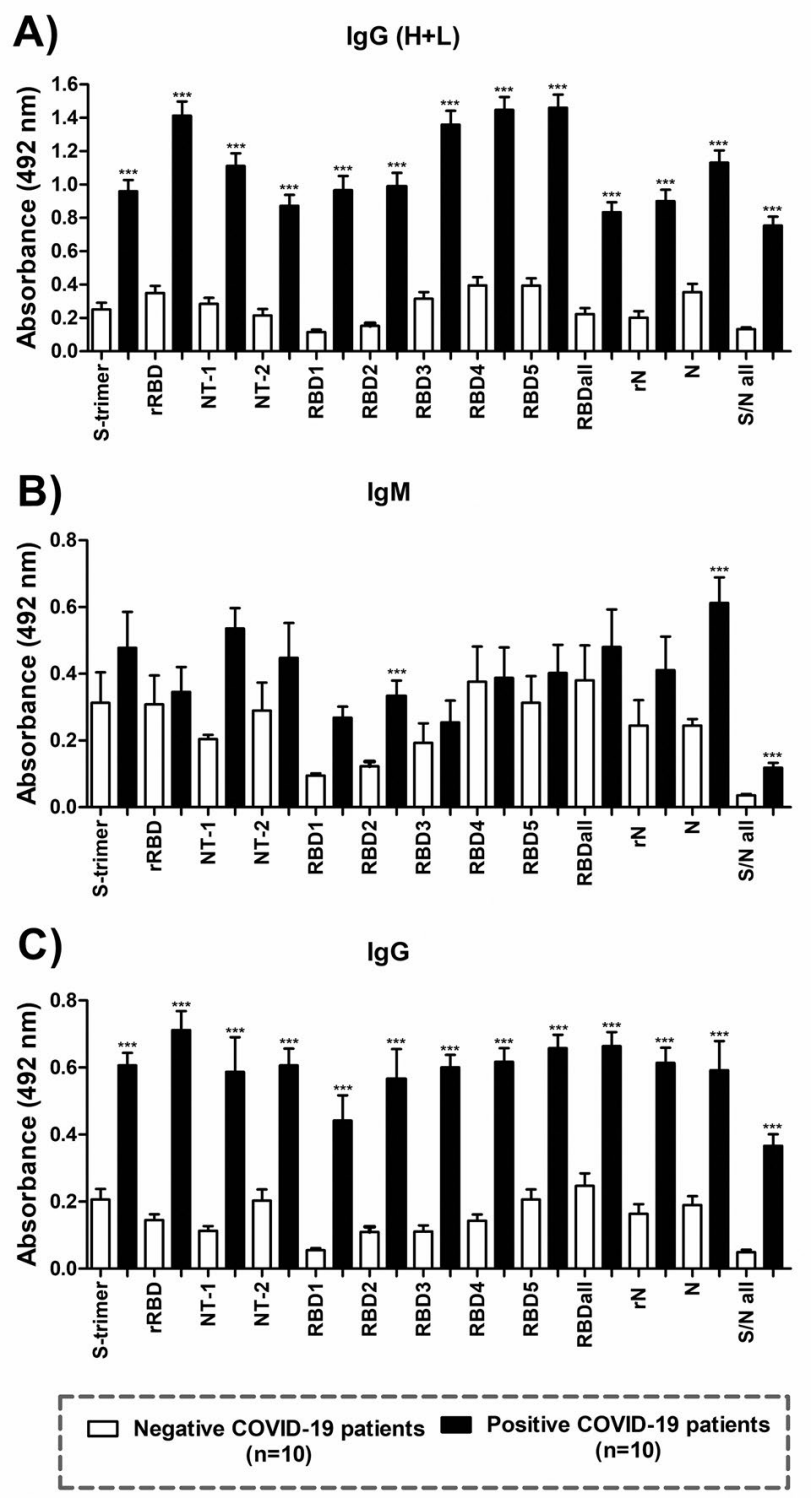
Antibody evaluation in serum samples derived from healthy and COVID-19 patients. Selected NT1, NT-2, RBD1-5 and N peptides were used as antigens, and recombinant proteins of S trimer, RBD and N were used at a final concentration of 0.1 µg/mL. Positive and negative serum samples from COVID-19 patients were used as primary antibodies, and anti-human IgG antibody (whole molecule), anti-mu and anti-gamma coupled to HRP were used as secondary antibodies. P value was calculated using one-way ANOVA: *** < 0.0001, * 0.01. Recombinant proteins are tagged as S trimer, rRBD and rN.

To evaluate the potential utility of the eight selected peptides and the full-length proteins for antibody detection by indirect ELISA, we used serum samples derived from healthy and COVID-19 patients (diagnosed by RT–PCR with at least 15 days since the onset of symptoms). The experiment included evaluation of IgM/IgG (whole molecule), IgM and IgG levels using recombinant proteins (S trimer, rRBD and rN), individual peptides (NT-1/2, RBD1-5 and N in MAP8 format) or a mixture of all the peptides found in protein S and all of them (S and N) at a final concentration of 0.1 µg/mL. We observed a high recognition of each individual peptide with at least four times the optical density of difference between healthy and COVID-19 patients in the detection of IgM/IgG. In relation to individual IgM and IgG detection, similar results were obtained, most of which exhibited statistically significant differences (Fig. 3) (p value < 0.001).

### Immune response induction in rabbits using MAP8 peptides derived from S and N structural proteins

**Figure 4:**
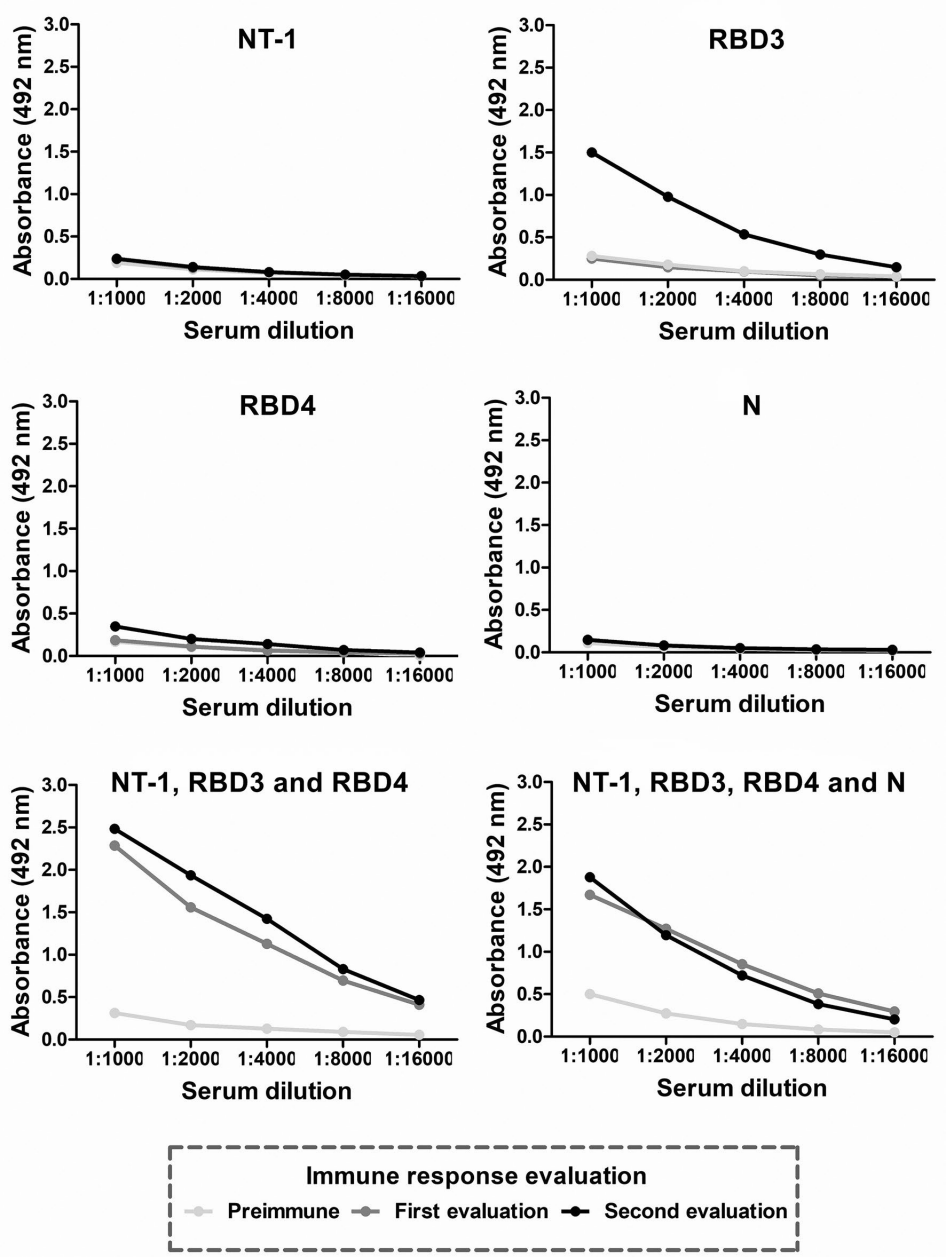
Antibody evaluation in New Zealand rabbits immunized with MAP8 peptides. Six New Zealand rabbits were immunized with 100 µg/mL of each individual peptide (NT-1, RBD3, RBD4 and N), a mixture of all peptides found in S (NT-1, RBD3, and RBD4) or a mixture of all peptides (N/S). Before immunizations, rabbits were bled, preimmune serum was obtained, and serum samples for the first and second evaluations were obtained 15 and 35 days after the first immunization. As antigens, peptides were used to a final concentration of 0.1 µg/mL, and anti-rabbit IgG antibody-HRP coupled was used as the secondary antibody.

Finally, we selected four peptides (NT-1, RBD3, RBD4 and N) for the immunization of New Zealand rabbits to test the capacity of selected peptides for humoral immune response induction. Over thirty days, three immunizations were performed using 100 µg of each peptide, the mixture of the peptides found in S protein or all peptides. Blood samples were collected before immunizations (pre-immune serum) and 15 (first evaluation) and 35 (second evaluation) days after the first immunization. In rabbits immunized with the peptides NT-1, RBD4 and N, no antibody response was observed until day 35, and the only individual peptide that was able to induce antibody response was RBD3. Both, the mixture of peptides situated in the S protein and the mixture of all peptides induced an antibody response (Fig. 4).

## Discussion

SARS-CoV-2 is responsible for COVID-19 infection; structurally, it is composed of four structural proteins called the spike (S), membrane (M), envelope (E) and nucleocapsid (N). Recombinant proteins of structural N and S (S1 and RBD subunit) proteins are commonly used as antigens in immunoassays for antibody detection, including lateral flow immunoassays (LFAs), enzyme-linked immunosorbent assays (ELISAs) and chemiluminescent immunoassays (CLIAs). However, some doubts regarding the utility of antibody tests have been voiced due to the antigenic similarity between SARS-CoV-2 and other circulating coronaviruses^21,22^. To avoid potential cross-reactivity, in silico analysis can be performed for the selection of immunogenic peptides that could be used for the standardization of a novel ELISA for antibody detection or the production of monoclonal and polyclonal antibodies for its use in the standardization of novel methods for viral antigen detection, which was the primary objective of this work.

The spike glycoprotein mediates the entry of coronavirus into host cells through angiotensin-converting enzyme 2 (ACE2) recognition, which is an attractive antiviral target and the most common antigen for antibody detection. The S protein is composed of two functional subunits, S1 and S2, the S1 subunit is composed of an N-terminal domain and receptor binding domain (RBD)^5,22^. The nucleocapsid protein physically links the envelope to the + RNA genome and contains N-terminal RNA binding (N-NTD) and C-terminal dimerization domains^23^. Both proteins were considered primary targets for epitope identification in this work.

Several in silico approaches have been performed to identify antiviral peptides^24^, epitopes for the development of vaccine-based peptides or multiepitope-based vaccines or diagnostics^25,26^. In this study, a total of twenty-five peptides were reported, nineteen of which were in the S protein and six of in the N protein. We selected eight peptides, two of which were located in the S1 NTD, five in the RBD and one in N. Interestingly, previous studies reported that each monomer of the spike protein has twenty-two glycosylation sites^27^, and this post-translational modification plays an important role in immune response evasion, function and viral pathobiology^28^. This approach indicates that the selected peptides, in addition to all the characteristics associated with immune response induction, may be used as antigens in the development of immunoassays. Interestingly, the amino acids present in the RBD3 peptide are almost exclusively located in the RBD of SARS-CoV-2 but are not found in SARS-CoV according to previous alignments performed by other authors^29,30^.

When we searched for the presence of mutations in SARS-CoV-2 VOCs and their effect on the sequence of the selected peptides, we found that in six of the eight peptides at least one amino acid change was identified. The RBD4 peptide contains the N501Y amino acid change in the receptor-binding domain of the S protein, which may contribute to the 40–70% increase in transmission observed with the Alpha, Beta, Gamma and Omicron variants^6^. The Delta variant contains amino acid changes in the RBD1 (N440K and G446S) and RBD3 (S477N and T478K) peptides, whereas Omicron has 4 amino acid changes in the RBD4 peptide (G496S, Q498R, N501Y, Y505H). These changes are commonly associated with the prevalence, phylogenetic relation and the neutralizing potency of each variant, for which future studies should be performed^7,31^. The emergence of new SARS-CoV-2 variants is a matter of concern for diagnostic testing, however the use of peptides in MAP8 format could be a novel strategy to solve problems associated with cross reactivity of recombinant proteins. An important consideration is the possibility of obtaining a negative result if only one peptide is used, due to amino acid changes that could affect antibody recognition. Therefore, we propose the mixture of two or more peptides for serosurveillance.

The eight selected peptides were synthetized in MAP8. This format increases the size and structural complexity of the peptide, allowing the exposition of eight epitopes in a single molecule, which would favor the use of low concentrations of antigen during its use for immunoassays (0.1 µg/mL in this study). Another interesting characteristic is that this format stimulates antibody production^32^. Using the aforementioned peptides, their use as antigens for antibody detection in COVID-19 patients using indirect ELISA and humoral immune response induction in rabbits was assessed. The use of peptides found in the N protein is important due to IgG seroconversion appears an average 2 days sooner than the S protein^33^, so the use of this peptide could be helpful in the detection of early immune response products. To evaluate the presence of antibodies, in addition to selected peptides, we also obtained recombinant proteins derived from the S (trimer and RBD) and N proteins. In both antigens, the results were considerably higher in COVID-19-positive patients than in COVID-19-negative patients for IgG and total antibodies. In the case of IgM, no significant difference was observed, which may be due to the time that had passed from the onset of symptoms to serum collection. A complete standardization of this method is still necessary, but preliminary results are worthy of a more in-depth investigation. Previously, antibodies against N and S proteins were tested, and no significant difference between the two proteins was observed for the detection of total antibodies and IgG^34^, which could mean that all reported peptides could be mixed during the development of the immunoassay to increase the number of available epitopes or used for the standardization of ELISA methods for each individual protein.

In relation with the use of peptides as antigens for the standardization of methods for antibody detection, several evidence has been published. Woo et al.^35^, developed an ELISA test for IgM and IgG antibody detection against the Spike and Nucleocapsid protein of SARS-CoV. Whereas, during the COVID-19 pandemic the use of antigenic epitopes derived from the structural proteins of the SARS-CoV-2 (Spike, Nucleocapsid, Membrane and Envelope) have been employed as antigens for the standardization of novel ELISA methods with high sensitivity (91.4%) and specificity (83.7%)^36^. In another study performed by Polvere et al., reported ten predicted peptides derived from the S, M and N proteins of SARS-CoV-2, of which, seven were tested by ELISA the reactivity to a panel of serum samples from twenty-four COVID-19 convalescents patients. Results showed differences in the reactivity to selected peptides ranging from the recognition to only one peptide to all seven peptides^37^. The ELISA method used in this study has several similarities with the aforementioned examples, thus providing evidence about the potential clinical utility of the reported peptides in the standardization of an ELISA method.

Although all peptides could be used as antigens for the search for antibodies against SARS-CoV-2, not all of them could be used for humoral immune response induction. The RBD3 peptide was the only peptide that induced a humoral immune response by itself, and the mixture of peptides could induce an immune response due to the increased complexity and molecular weight of the combined peptides. The RBD3 peptide is highly conserved in VOCs Alpha, Beta, Gamma and Omicron, therefore the serum derived from the immunized rabbits could be used for analysis of neutralizing activity against these VOCs. A notable result was that the RBD4 peptide that contains N501Y (and three additional amino acid changes in the Omicron variant), did not induce humoral immune response in rabbits, but was recognized by the serum samples derived from patients.

Positive immune response induction in rabbits and the recognition of peptides by serum samples derived from COVID-19 patients provide evidence regarding the interaction of selected peptides with MHC molecules, T cell response, and B cell activation. The use of synthetic peptides for immune response induction has the advantage that polyclonal antiserum in theory will be as specific as monoclonal antibody but much easier to produce, and synthetic peptides are cheaper and less time-consuming to produce than the synthesis of other antigens, such as recombinant proteins^38^. Some experiments still need to be performed to assess the clinical application of peptides as antigens (for serosurveillance of VOCs) or in the production of antibodies for antigen detection; however, our preliminary results provide not only in silico approaches (localization in 2D and 3D structure, post-translational modifications and analysis of VOC) but also experimental data comparing peptides to the full-length protein. In addition, we provide a list of seventeen other peptides that could be tested with several applications, such as immunoassay development, antibody production or vaccine candidates.

Experimental procedures to be performed include the standardization of ELISA methods using reported peptides for antibody detection in vaccinated or infected patients (with circulating VOC or new variants) or the recognition of native protein using serum derived from immunized rabbits. In conclusion, this work provides evidence regarding the potential utility of MAP8 peptides selected based on in silico analysis of the primary amino acid structure of the spike glycoprotein and nucleocapsid protein of SARS-CoV-2 for the standardization of novel immunoassays for antibody detection in serum samples of COVID-19 patients and vaccinated individuals or for antibody production in animal models that can be used for the detection of antigens in infected individuals.

## Materials and methods

### In silico analysis

First, we downloaded the amino acid sequences of the S (access number QJX59884.1) and N (access number QJX60131.1) proteins of SARS-CoV-2 from the NCBI protein database (https://www.ncbi.nlm.nih.gov/). The amino acid sequence of the S protein was divided into two segments: S1 (14–685) and S2 (686–1273). In S1, subdivision was performed as S1 NTD (N-terminal domain) from amino acids 14–305 and S1 receptor binding domain (RBD) from amino acids 319–685. Considering the importance of the S1 domain during SARS-CoV-2 infection, immune response, and diagnosis, most of our efforts were allocated to analyzing this specific region, although our analysis also included the S2 domain.

Using the aforementioned sequences, in silico analysis was performed using the B cell epitope prediction tools of the IEDB analysis resource, specifically the methods for predicting continuous antibody epitopes (http://tools.iedb.org/bcell/) from protein sequences as follows: Bepipred-1.0 (linear epitope prediction) and 2.0 (sequential B cell epitope predictor), Emini surface accessibility scale and Kolaskar and Tongaonkar antigenicity scale. Additionally, complementary analysis was performed using BepiPred-2.0 (http://www.cbs.dtu.dk/services/BepiPred/index.php) to search for B cell epitopes and predicted antigenic peptides (http://imed.med.ucm.es/Tools/antigenic.pl) to search for antigenic peptides. All predicted peptides are shown in Supplementary Material S1, and the number of obtained peptides is shown in Table 1. For the second selection, all peptides with a length < 8 amino acids were filtered and deleted, and we considered the presence of at least two different properties (accessibility, antigenicity, or immunogenicity) for preselection. Finally, we performed conservancy analysis using the IEBD analysis resource (http://tools.iedb.org/conservancy/), nucleotide BLAST (https://blast.ncbi.nlm.nih.gov/Blast.cgi) and considered the localization of each peptide in the primary, secondary and tertiary structures.

### PDB accession, 3D structure modeling and visualization of S and N proteins

The PDB of full-length SARS-CoV-2 S protein in the open state and glycans was obtained from The Amaro Lab (https://amarolab.ucsd.edu/covid19.php), which was manually renumbered by eliminating the membrane, water molecules and ions. The 3D structure of the complete N protein was predicted for homology modeling using the Robetta program by Baker Lab, and PDB 7ACS and 6YUN (https://robetta.bakerlab.org/) were used as templates. All 3D structures were visualized using the VMD 1.9.1 program (Visual Molecular Dynamics), which is a molecular visualization program for displaying, animating, and analyzing large biomolecular systems using 3D graphics with built-in scripting (http://www.ks.uiuc.edu/Research/vmd/).

### Schematic representations of N and S protein

The schematic representation (at scale) of the amino acid sequences of S and N proteins, including domains and localization, was illustrated using the Illustrator of Biological Sequences (IBS)^39^. The 2D schematic representation of both proteins was created in Protter (https://wlab.ethz.ch/protter/start/), an interactive and customizable web-based application that enables the integration and visualization of both annotated and predicted protein sequence features together with experimental proteomic evidence for peptides and PTMs, onto the transmembrane topology of a protein^40^. For both illustrations, we used UniProt protein accession P0DTC2 for the S protein and P0DTC9 for the N protein.

### Purification of SARS-CoV-2 S, RBD and N proteins

The SARS-CoV-2 S HexaPro plasmid (Addgene Cat#154754) was transfected into HEK293F cells, a human embryonic kidney cell line, and the cells were grown in 293FreeStyle expression medium (Life Technologies Cat#12338002) and cultured at 37 °C with 5% CO_2_. The supernatant was recovered, centrifuged, filtered and purified by immobilized metal affinity chromatography (IMAC) under native conditions. The resulting protein had six prolines substituted, a substitution of “GSAS” (furin cleavage site) and the addition of a C-terminal foldon trimerization motif with an eight histidine tag that generates a prefusion-stabilized SARS-CoV-2 S protein. The SARS-CoV-2 receptor binding domain (RBD) was amplified from a template of cDNA derived from a clinical isolation of SARS-CoV-2 and cloned into pLATE-31 according to the protocol of the aLICator Ligation Independent Cloning and Expression System (Cat# K1261 Thermo Scientific). The cDNA of SARS-CoV-2 protein N was also obtained from clinical isolation and cloned into pLATE-51 (Cat# K1251 Thermo Scientific). pLATE-RBD with C-terminal hexahistadine (or 6xHIS) tags and pLATE-51-N with six N-terminal histidine tags were used for the transformation of the Rosetta strain of *E. coli,* and IMAC purification was performed under native conditions.

### Analysis of SARS-CoV-2 variants of concern

We used CoVariants (https://covariants.org/) for the overview of mutations in SARS-CoV-2 variants: Alpha (B.1.1.7), Beta (B.1.351), Gamma (P.1), Delta (B.1.617.2) and Omicron (BA.1). Then, we searched for amino acid changes and deletions that define each variant and their localization in the sequence of all selected peptides.

### Peptides synthesis

The multiantigenic peptide 8 (MAP8) format allows the synthesis of peptides with a length of < 15 residues. The synthesis was performed using PepMic (http://www.pepmic.com/) and provided information including mass spectrometry (MS) and high-performance liquid chromatography (HPLC). We extracted the theoretical and observed molecular weight and purity from the technical sheet. Each peptide was dissolved to a final concentration of 1 mg/mL and used as antigen for antibody detection in serum samples derived from healthy and COVID-19 patients and for the immunization of New Zealand rabbits. Peptides were called NT-1 (RTQLPPAYTNS), NT-2 (MESEFRVYSSANN), RBD1 (SNNLDSKVGGNY), RBD2 (RLFRKSNLKPFE), RBD3 (ISTEIYQAGST), RBD4 (YGFQPTNGVGYQ), RBD5 (GPKKSTNLVKNK) and N (MSDNGPQNQRNA).

### Sample selection and peptide evaluation using enzyme-linked immunosorbent assay (ELISA)

Ten serum samples derived from patients with positive qRT–PCR for SARS-CoV-2 detection with at least fifteen days of infection and ten serum samples from patients with negative diagnoses for SARS-CoV-2 who reported no previous contact with an infected person were used. During all the performed experiments, potentially infectious samples were processed in a biosafety cabinet or inactivated at 56 °C during 1 h. For peptide evaluation, microtiter plates (Sigma Aldrich Cat # CLS3590) were coated with individual recombinant proteins (trimeric spike, recombinant RBD or recombinant N protein), individual peptides (NT1-2, RBD1-5, and N), a mixture of the peptides located in S or all peptides (at a final concentration of 0.1 μg/mL) by adding 100 μL of peptide solution to coating buffer (50 mM Na2CO3/NaCO3H pH 9.6) and incubated overnight at 4 °C. Plates were blocked for 40 min at room temperature (RT) in 200 μL 5% skimmed milk in PBS-Tween (0.05%) and then incubated with human serum (1:50 dilution) for 1 h at 37 °C. After washing, plates were incubated with 100 μL anti-human IgG (whole molecule dilution 1:4000; Sigma–Aldrich Cat# 401455), anti-human IgG (gamma-specific dilution 1:3000; Sigma–Aldrich Cat#A8419) and anti-human IgM (mu-specific dilution 1:4000; Sigma–Aldrich Cat#A0420) coupled to HRP for 1 h at 37 °C. The enzymatic reaction was developed using O-phenylenediamine dihydrochloride (Sigma Aldrich Cat # P1526) and was stopped by the addition of 2 N H_2_SO_4_. The optical density was measured at 492 nm using a microplate reader.

### New Zealand rabbit immunization and immune response evaluation

Six New Zealand rabbits were subcutaneously immunized with 100 µg of each selected peptide in the following order: NT-1 (rabbit #1), RBD3 (rabbit #2), RBD4 (rabbit #3), N (rabbit #4), mixture of all peptides in S protein (rabbit #5) and mixture of all four peptides (rabbit #6) using complete (Life Technologies Cat # P1503) (first immunization) and incomplete Freund’s (Sigma Aldrich Cat # F5506) (second and third immunization) as adjuvants. A total of three immunizations were performed with an interval of ten days each. For immune response evaluation, rabbits were bled before immunizations (preimmune serum) and 15 (first evaluation) and 35 (second evaluation) days after the initial immunization. All collected blood samples were evaluated for immune response evaluation using indirect ELISA.

Immune response evaluation was performed similarly to the abovementioned ELISA with the following specific differences: antigen fixation was performed using 1 µg/mL of each peptide incubated for 2 h at 37 °C, serial dilutions (1:1000–1:16,000) of the sera were performed and incubated overnight at 4 °C. Finally, an anti-rabbit IgG-HRP coupled (Santa Cruz Biotechnology Cat # sc-2357) was used as a secondary antibody and incubated for 2 h at 37 °C.

### Analysis

Obtained data was analyzed using GraphPad Prism V.5.0. For statistical analysis, we performed ANOVA test with Bonferroni adjustment and a p value < 0.05 was considered statistically significant.

### Ethics declarations

All enrolled patients signed an informed consent form for serum collection considering the Helsinki Declaration, and this study was approved by the Ethics Committee of the Universidad Autónoma de Guerrero. Furthermore, the six rabbits were maintained in individual cages (with individual labels) and the number of animals was calculated considering the number of individual peptides and the potential combinations all procedures. All immunizations and bleeding were performed in the same order. For animal treatment, we consider the ARRIVE guidelines (https://arriveguidelines.org). Also, all procedures involving animal manipulation were approved by the ethical committee of the Universidad Autónoma de Guerrero with registry number CICUAL-01/2021, and experiments were performed in accordance with NOM-062-ZOO-1999.

## Supplementary Information

Supplementary Information 1.

Supplementary Information 2.

Supplementary Information 3.

Supplementary Information 4.

Supplementary Information 5.

## Data Availability

All data and supporting data associated with the conception and experiments is available in the supporting information.

## References

[1] Liu Y-C, Kuo R-L, Shih S-R (2020). COVID-19: The first documented coronavirus pandemic in history. Biomed. J..

[2] Huang C (2020). Clinical features of patients infected with 2019 novel coronavirus in Wuhan China. The Lancet.

[3] Rastogi M, Pandey N, Shukla A, Singh SK (2020). SARS coronavirus 2: from genome to infectome. Respir. Res..

[4] Wu A (2020). Genome composition and divergence of the novel coronavirus (2019-nCoV) originating in China. Cell Host Microbe.

[5] Wang M-Y (2020). SARS-CoV-2: Structure, biology, and structure-based therapeutics development. Front. Cell. Infect. Microbiol..

[6] Fontanet A (2021). SARS-CoV-2 variants and ending the COVID-19 pandemic. The Lancet.

[7] Kannan SR (2022). Omicron SARS-CoV-2 variant: Unique features and their impact on pre-existing antibodies. J. Autoimmun..

[8] Tao K (2021). The biological and clinical significance of emerging SARS-CoV-2 variants. Nat. Rev. Genet..

[9] Loeffelholz MJ, Tang Y-W (2020). Laboratory diagnosis of emerging human coronavirus infections—the state of the art. Emerg. Microbes Infect..

[10] Böger B (2021). Systematic review with meta-analysis of the accuracy of diagnostic tests for COVID-19. Am. J. Infect. Control.

[11] Dramé M (2020). Should RT-PCR be considered a gold standard in the diagnosis of COVID-19?. J. Med. Virol..

[12] Machado B, Hodel K, Barbosa-Júnior V, Soares M, Badaró R (2020). The main molecular and serological methods for diagnosing COVID-19: An overview based on the literature. Viruses.

[13] Carter LJ (2020). Assay techniques and test development for COVID-19 diagnosis. ACS Cent. Sci..

[14] Abraham Peele, K., Srihansa, T., Krupanidhi, S., Ayyagari, V. S. & Venkateswarulu, T. C. Design of multi-epitope vaccine candidate against SARS-CoV-2: A in-silico study. *J. Biomol. Struct. Dyn.* **39**, 3793–3801 (2021). 10.1080/07391102.2020.1770127 PMC7284139 32419646

[15] He J (2021). Vaccine design based on 16 epitopes of SARS-CoV-2 spike protein. J. Med. Virol..

[16] Lee E (2021). Identification of SARS-CoV-2 nucleocapsid and spike T-cell epitopes for assessing T-cell immunity. J. Virol..

[17] Sharma A, Pal S, Panwar A, Kumar S, Kumar A (2021). In-silico immunoinformatic analysis of SARS-CoV-2 virus for the development of putative vaccine construct. Immunobiology.

[18] Shehata MM (2021). In silico and in vivo evaluation of SARS-CoV-2 predicted epitopes-based candidate vaccine. Molecules.

[19] Rahman MS (2020). Epitope-based chimeric peptide vaccine design against S, M and E proteins of SARS-CoV-2 etiologic agent of global pandemic COVID-19: an in silico approach. PeerJ.

[20] Singh A, Thakur M, Sharma LK, Chandra K (2020). Designing a multi-epitope peptide based vaccine against SARS-CoV-2. Sci. Rep..

[21] Kirtipal N, Bharadwaj S, Kang SG (2020). From SARS to SARS-CoV-2, insights on structure, pathogenicity and immunity aspects of pandemic human coronaviruses. Infect. Genet. Evol..

[22] Theel, E. S. *et al.* The role of antibody testing for SARS-CoV-2: Is there one? *J. Clin. Microbiol.* **58**, (2020). 10.1128/JCM.00797-20 PMC7383527 32350047

[23] Dinesh DC (2020). Structural basis of RNA recognition by the SARS-CoV-2 nucleocapsid phosphoprotein. PLoS Pathog..

[24] Ling R (2020). In silico design of antiviral peptides targeting the spike protein of SARS-CoV-2. Peptides.

[25] Can H (2020). In silico discovery of antigenic proteins and epitopes of SARS-CoV-2 for the development of a vaccine or a diagnostic approach for COVID-19. Sci. Rep..

[26] Tahir ul Qamar, M. *et al.* Designing of a next generation multiepitope based vaccine (MEV) against SARS-COV-2: Immunoinformatics and in silico approaches. *PLoS One* **15**, e0244176 (2020). 10.1371/journal.pone.0244176 PMC7755200 33351863

[27] Watanabe Y, Allen JD, Wrapp D, McLellan JS, Crispin M (2020). Site-specific glycan analysis of the SARS-CoV-2 spike. Science.

[28] Watanabe, Y., Bowden, T. A., Wilson, I. A. & Crispin, M. Exploitation of glycosylation in enveloped virus pathobiology. *Biochim. Biophys. Acta (BBA) General Subjects* **1863**, 1480–1497 (2019). 10.1016/j.bbagen.2019.05.012 PMC6686077 31121217

[29] Lan J (2020). Structure of the SARS-CoV-2 spike receptor-binding domain bound to the ACE2 receptor. Nature.

[30] Walls AC (2020). Structure, Function, and Antigenicity of the SARS-CoV-2 Spike Glycoprotein. Cell.

[31] Chen, X. *et al.* Neutralizing antibodies against severe acute respiratory syndrome coronavirus 2 (SARS-CoV-2) variants induced by natural infection or vaccination: A systematic review and pooled analysis. *Clin. Infect. Dis.* ciab646 (2021). 10.1093/cid/ciab646. 10.1093/cid/ciab646 PMC9016754 34302458

[32] Joshi VG, Dighe VD, Thakuria D, Malik YS, Kumar S (2013). Multiple antigenic peptide (MAP): A synthetic peptide dendrimer for diagnostic, antiviral and vaccine strategies for emerging and re-emerging viral diseases. Indian J. Virol..

[33] Van Elslande J (2020). Antibody response against SARS-CoV-2 spike protein and nucleoprotein evaluated by four automated immunoassays and three ELISAs. Clin. Microbiol. Infect..

[34] Liu, W. *et al.* Evaluation of nucleocapsid and spike protein-based enzyme-linked immunosorbent assays for detecting antibodies against SARS-CoV-2. *J. Clin. Microbiol.* **58**, (2020). 10.1128/JCM.00461-20 PMC7269413 32229605

[35] Woo PCY (2005). Differential sensitivities of severe acute respiratory syndrome (SARS) coronavirus spike polypeptide enzyme-linked immunosorbent assay (ELISA) and SARS coronavirus nucleocapsid protein ELISA for serodiagnosis of SARS coronavirus pneumonia. J. Clin. Microbiol..

[36] Arévalo, A. *et al. ELIPSE-COL: A novel ELISA test based on rational envisioned synthetic peptides for detection of SARS-CoV-2 infection in Colombia*. 10.1101/2020.11.13.20230060 (2020).

[37] Polvere I (2022). A peptide-based assay discriminates individual antibody response to SARS-CoV-2. Genes Dis..

[38] Van Regenmortel MHV (2001). Antigenicity and immunogenicity of synthetic peptides. Biologicals.

[39] Liu W (2015). IBS: An illustrator for the presentation and visualization of biological sequences. Bioinformatics.

[40] Omasits U, Ahrens CH, Müller S, Wollscheid B (2014). Protter: Interactive protein feature visualization and integration with experimental proteomic data. Bioinformatics.

