# Supplementary material for: Utility of in silico-identified-peptides in spike-S1 domain and nucleocapsid of SARS-CoV-2 for antibody detection in COVID-19 patients and antibody production
Source: Sci Rep. 2022 Sep 5;12:15057. doi: 10.1038/s41598-022-18517-w (PMC9442563; doi:10.1038/s41598-022-18517-w)
Supplement: Supplementary file 1 — Supplementary Information 1. [file 41598_2022_18517_MOESM1_ESM.docx]

**Supplementary material 1.- Complete list of immunogenic and antigenic peptides found in S and N proteins.**

|  | **IEDB analysis resource** | | | |  | |
| --- | --- | --- | --- | --- | --- | --- |
| **SARS-CoV-2** | **Linear epitopes** | **Sequential epitopes** | **Surface accessibility** | **Antigenicity** | **BepiPred-2.0** | **PAP** |
| **S protein** |  |  |  |  |  |  |
| **S1**  **NTD** | RTQLPPAYTNS,SGTNGTKRFDN,LTPGDSSSGWTAG. | SQCVNLTTRTQLPPAYTN,SNVTWFHAIHVSGTNGTKRF,FLGVYYHKNNKSWME,SEFRVYSSANN,MDLEGKQGNFKNLRE,INLVRDLPQGFSA,YLTPGDSSSGWTAG,CALDPLSETK. | LTTRTQLPPAYTNSF,GVYYPDKVF,  TNGTKRFD,LDSKTQ,YYHKNNKSWM,LEGKQGN,KIYSKHT,KYNENG. | FLVLLPLVSSQCVNL,RSSVLHST,DLFLPFFS,FHAIHV,NPVLPFN,QSLLIVN,NVVIKVCEFQ,CNDPFLGVYYH,FEYVSQP,INLVRDL,LEPLVDLP,QTLLALHRSY,AAYYVGYL,PRTFLLK,AVDCALDP. | SQCVNLTTRTQLPPAYTNS,SNVTWFHAIHVSGTNGTKRF,FLGVYYHKNNKSWMESEFRVYSSANN,MDLEGKQGNFKNLRE, INLVRDLPQGFSA,YLTPGDSSSGWTAG,CALDPLSETK. | FLVLLPLVSSQCVNL,TRGVYYPDKVFRSSVLHSTQDLFLPFFSNVTWFHAIHV,DNPVLPFNDGVYFA, NATNVVIKVCEFQFCNDPFLGVYY,TFEYVSQPFLM,TPINLVRDLPQGFSALEPLVDLPIG,RFQTLLALHRSYLT,GAAAYYVGYLQPRTFLL,ITDAVDCALDP. |
| **S1**  **RBD** | VRQIAPGQTGKIAD,NNLDSKVGG,YQAGSTPCNGV,YGFQPTNGVGYQ,TVCGPKKST. | GIYQTSNFRVQ,VFNATRFASVYAWNR,KRISNCVA,ASFSTFK,  RGDEVRQIAPGQTGK,IADYNYKLPD,NNLDSKVGGNYNYLY,RLFRKSNLKPFERD,ISTEIYQAGSTPCNGVEGF,CYFPLQSYGFQPTNGVGYQ,APATVCGPKKSTNLVKNKC. | FRVQPT,ADYNYKLPDD,NSNNLD,  LFRKSNLKPFERDI,YGFQPT. | TNLCPFG,SNCVADYSVLYNS,TFKCYGVSPT,TGCVIA,CYFPLQSY,YQPYRVVVLSFEL,LHAPATVCGP. | GIYQTSNFRVQ,VFNATRFASVYAWNRKRISNCVA,RGDEVRQIAPGQTGKIADYNYKLPD,NNLDSKVGGN,LFRKSNLKPFERDISTEIYQAGSTPCNGVEGFNCYFPLQSYGFQPTNGVGYQP,LHAPATVCGPKKSTNLVKNK. | SNFRVQPTESIVRF,NITNLCPFGE,RISNCVADYSVLYNS,SFSTFKCYGVSP,KLNDLCFTNVYADSFVIR,STEIYQAGS,EGFNCYFPLQSYG,VGYQPYRVVVLSFELLHAPATVCGP. |
| **S2** | RDIADTTDAVRDPQ,VITPGTNTSN,QTQTNSPRRARSV,VEQDKNTQE,IYKTPPIKD,ILPDPSKPSKRS,PAQEKNFTT,VYDPLQPELDSF,KNHTSPDVDLG,FDEDDSEPVL. | HADQLTPTWRVYSTGSNVFQT,NNSYECDIPIGA,CASYQTQTNSPRRARSVASQSIIAYTMSLGAENSVAYSNN,VEQDKNTQEVFAQVKQIYKTPPIKDFGGFNLPDPSKPSKRS,LTDAGFIKQYGDCLGD,  DKVEAEVQID,GQSKRVDFC,RNFYEPQIITTD,VNNTVYDPLQPELDS,FKEELDKYFKNHTS,PDVDLGDISGINA,ESLIDLQELGKYE,CCKFDEDDSEPVLKGVK. | ESNKKF,DIADTTDAVRDPQT,GTNTSN,DQLTPTWRVY,HVNNSY,YQTQTNSPRRAR,EQDKNTQ,KQIYKTPPI,  DPSKPSKRSF,NVLYENQ,VPAQEKNFT,TQRNFYE,DPLQPELDSFKEELDKYFKNHTSP,IQKEIDRL,ELGKYEQYIKWP,FDEDDS. | FGGVSVIT,QVAVLYQDV,CTEVPVAIHAD,AGCLIGA,GAGICASY,VASQSII,TTEILPVS,  SVDCTMY,SNLLLQYGSFCTQL,VFAQVKQI,SQILPD,  YGDCLGD,RDLICAQ,LTVLPPL,YTSALLAG,LNTLVKQL,ISSVLND,SLQTYVTQQ,SECVLGQS,PHGVVFLHVTYVPA,PAICHDG,SGNCDVVIGI,ASVVNI,IAGLIAIVMVTIMLCCMTSCCSCLKGCCSCGSCCKF. | IHADQLTPTWRVYSTGSNVF,HVNNSYECDIPIG,CASYQTQTNSPRRARSVASQSIIAYTMSLGAENSVAYSN,VEQDKNTQEVFAQVKQIYKTPPIKDFGGFNFSQILPDPSKPSKRS,LTDAGFIKQYGDCLGD,DKVEAEVQIDR,GQSKRVDFC,QRNFYEPQIITTD,NTVYDPLQPELDSFKEELDKYFKNHTSPDVDLGDISGINA,CKFDEDDSEPVLKGVK. | QTLEILDITPCSFGGVSVIT,NQVAVLYQDVNCTEVPVAIHADQ,PTWRVYSTGSNVFQTRAGCLIGA,YECDIPIGAGICASY,RARSVASQSIIAYTM,FTISVTTEILPVS,TSVDCTMYICGD,ECSNLLLQYGSFCTQLN,QEVFAQVKQIYKT, NFSQILPDP, FIEDLLFNKVTL,FIKQYGDCLGD,AARDLICAQ,NGLTVLPPLLT,AQYTSALLAG,AGAALQIPF,VTQNVLYENQ,IGKIQDSLSSTASALGKLQDVVNQ,ALNTLVKQLSS,AISSVLNDILSRLDKVEAEVQIDR,ITGRLQSLQTYVTQQLIRA,KMSECVLGQ,KRVDFCGKGYHLMSFPQSAPHGVVFLHVTYVPA,TAPAICHDG,PREGVFVS,VSGNCDVVIGIVNN,VYDPLQPEL,GINASVVNI,NESLIDLQE,WYIWLGFIAGLIAIVMVTIMLCCMTSCCSCLKGCCSCGSCCK,SEPVLKGVK. |
| **N** | MSDNGPQNQRNAPRI,TFGGPSDSTGSNQNGERSGARSKQRRPQGLPNNTAS,QHGKEDLKFPRGQGVPINTNSSPDDQIG,RIRGGDGKMKDL,TGPEAGLPYGANK,GALNTPKDHIGTRNPANN,GTTLPKGFYAEGSRGGSQASSRSSSRSRNSSRNSTPGSSRGTSPARMAGNGGD,SKMSGKGQQQQGQTVTKKSAAEASKKPRQKRTATKAYN,AFGRRGPEQTQGNFG,EVTPSGTWL,KLDDKDPNFK,KTFPPTEPKKDKKKKADETQALPQRQKKQQ. | NGPQNQRNAPRI,FGGPSDSTGSNQNGE,RSGARSKQRRPQGLPNN,HGKEDLKFPRGQGVPINTNSSPDDQIGYYRRATRRIRGGDGKMKDLS,AGLPYGANK,  GALNTPKDHIGTRNP,ANNAAIVLQLPQ,TTLPKGFYAEGSRGGSQASSRSSSRSRNSSRNSTPGSSRGTSPARMAGNGGD,RLNQLESKMSGKGQQQQGQTVTKKSAAEASKKPRQKRTATKA,RRGPEQTQGNFGDQELIRQGTDYK,  DPNFKD,DAYKTFPPTEPKKDKKKKADETQALPQRQKKQQTVTLLPAADLDDSKQLQQSMSSADS. | NGPQNQRN,RSKQRRP,YRRATR,RSSSRSRNSSRNSKGQQQQ,ASKKPRQKRTA,RGPEQT,GTDYKH,DDKDPNF,  PTEPKKDKKKKAD,QRQKKQQ. | WFTALTQH,GQGVPIN,QIGYYRR,PRWYFYYLGT,AGLPYG,IIWVATE,NAAIVLQLPQGTT,AALALLLLDRL,GQTVTKK,AYNVTQA,KHWPQIAQFAPSASAFF,YTGAIKL,KDQVILLNKHIDAYKTF,TQALPQR,QQTVTLLPAADLDFSKQLQQSM. | NGPQNQRNAPRITFGGPSDSTGSNQNGERSGARSKQRRPQGLPNN,HGKEDLKFPRGQGVPINTNSSPDDQIGYYRRATRRIRGGDGKMKDLS,GALNTPKDHIGTRNPANNAAI,TLPKGFYAEGSRGGSQASSRSSSRSRNSSRNSTPGSSRGTSPARMAGNGGD,LNQLESKMSGKGQQQQGQTVTKKSAAEASKKPRQKRTATK,RRGPEQTQGNFGDQELIRQGTDYK,DAYKTFPPTEPKKDKKKKADETQALPQRQKKQQTVTLLPAADLDDFSKQLQQSMSSADS. | SPRWYFYYLG,NNAAIVLQLPQGT,DAALALLLLDR,YKHWPQIAQFAPSASAF,FKDQVILLNKHIDAYKT,KQQTVTLLPAADL,DFSKQLQQS. |
