# Supplementary material for: Utility of in silico-identified-peptides in spike-S1 domain and nucleocapsid of SARS-CoV-2 for antibody detection in COVID-19 patients and antibody production
Source: Sci Rep. 2022 Sep 5;12:15057. doi: 10.1038/s41598-022-18517-w (PMC9442563; doi:10.1038/s41598-022-18517-w)
Supplement: Supplementary file 2 — Supplementary Information 2. [file 41598_2022_18517_MOESM2_ESM.docx]

**Supplementary material 2.- Color patterns used for the identification of each peptide in the 3D structure.**

| **Epitope** | **Protein** | **Domain** | **Epitope sequence*** | **Epitope length** | **Position** |
| --- | --- | --- | --- | --- | --- |
| 1 | S | S1 | SQCVNLTT | 8 | 13-20 |
| 2 |  |  | **RTQLPPAYTNS (1)** | 11 | 21-31 |
| 3 |  |  | GVYYPDKVF | 9 | 35-43 |
| 4 |  |  | SNVTWFHAIHVSGTNGTKRF | 20 | 60-79 |
| 5 |  |  | FLGVYYHKNNKSWME | 15 | 140-154 |
| 6 |  |  | **MESEFRVYSSANN (2)** | 13 | 153-165 |
| 7 |  |  | INLVRDLPQGFSA | 13 | 210-222 |
| 8 |  |  | LTPGDSSSGWTAG | 13 | 249-261 |
| 9 |  |  | VRQIAPGQTGKIAD | 14 | 407-420 |
| 10 |  |  | **SNNLDSKVGGNYNYLY (3)** | 15 | 438-453 |
| 11 |  |  | **RLFRKSNLKPFERD (4)** | 14 | 454-467 |
| 12 |  |  | **ISTEIYQAGSTPCNGVEGF (5)** | 19 | 468-486 |
| 13 |  |  | **YGFQPTNGVGYQ (6)** | 12 | 495-506 |
| 14 |  |  | **GPKKSTNLVKNK (7)** | 12 | 526-537 |
| 15 |  |  | RDIADTTDAVRDPQ | 14 | 567-580 |
| 16 | S | S2 | QTQTNSPRRARSV | 13 | 675-687 |
| 17 |  |  | ILPDPSKPSKRS | 12 | 805-816 |
| 18 |  |  | VPAQEKNFTT | 10 | 1068-1077 |
| 19 |  |  | FDEDDSEPVL | 10 | 1256-1265 |
| 20 | N |  | **MSDNGPQNQRNAPRIT** | 16 | 1-16 |
| 21 |  |  | AALALLLLDRL | 11 | 217-227 |
| 22 |  |  | KHWPQIAQFAPSASAFF | 17 | 299-315 |
| 23 |  |  | RIRGGDGKMKDL | 12 | 93-104 |
| 24 |  |  | SPRWYFYYLG | 10 | 105-114 |
| 25 |  |  | ILLNKHIDAYKT | 12 | 351-362 |

*In red: selected peptides in protein S. Numbers indicate the position of each peptide in figure 2.

**Supplementary material 4.- Conservancy analysis of the selected peptides and other Coronavirus-related virus.**

|  | **Access number** | **Epitope Conservancy Analysis Result** | | | | | | |
| --- | --- | --- | --- | --- | --- | --- | --- | --- |
| **Name** | **Human** | **NT-1** | **NT-2** | **RBD1** | **RBD2** | **RBD3** | **RBD4** | **RBD5** |
| **Severe acute respiratory syndrome coronavirus 2** | YP_009724390.1 | 100% | 100% | 100% | 100% | 100% | 100% | 100% |
| **SARS coronavirus Tor2** | YP_009825051.1 | 0% | 0% | 0% | 0% | 0% | 0% | 0% |
| **Middle East respiratory syndrome-related coronavirus** | YP_009047204.1 | 0% | 0% | 0% | 0% | 0% | 0% | 0% |
| **Middle East respiratory syndrome-related coronavirus** | AHX00731.1 | 0% | 0% | 0% | 0% | 0% | 0% | 0% |
| **Human betacoronavirus 2c England-Qatar/2012** | AGG22542.1 | 0% | 0% | 0% | 0% | 0% | 0% | 0% |
| **Human coronavirus NL63** | YP_003767.1 | 0% | 0% | 0% | 0% | 0% | 0% | 0% |
| **Human coronavirus 229E** | NP_073551.1 | 0% | 0% | 0% | 0% | 0% | 0% | 0% |
| **Human coronavirus 229E** | ABB90529.1 | 0% | 0% | 0% | 0% | 0% | 0% | 0% |
| **Human coronavirus HKU1** | YP_173238.1 | 0% | 0% | 0% | 0% | 0% | 0% | 0% |
| **Human coronavirus HKU1** | ADN03339.1 | 0% | 0% | 0% | 0% | 0% | 0% | 0% |
| **Human coronavirus OC43** | YP_009555241.1 | 0% | 0% | 0% | 0% | 0% | 0% | 0% |
| **Host** | **Animals** |  |  |  |  |  |  |  |
| **Miniopterus bat coronavirus HKU8** | YP_001718612.1 | 0% | 0% | 0% | 0% | 0% | 0% | 0% |
| **Rousettus bat coronavirus HKU9** | YP_001039971.1 | 0% | 0% | 0% | 0% | 0% | 0% | 0% |
| **Beluga whale coronavirus SW1** | YP_001876437.1 | 0% | 0% | 0% | 0% | 0% | 0% | 0% |
| **Bottlenose dolphin coronavirus HKU22** | AHB63508.1 | 0% | 0% | 0% | 0% | 0% | 0% | 0% |
| **Night heron coronavirus HKU19** | AFD29226.1 | 0% | 0% | 0% | 0% | 0% | 0% | 0% |
| **Porcine coronavirus HKU15** | AFD29187.1 | 0% | 0% | 0% | 0% | 0% | 0% | 0% |
| **Munia coronavirus HKU13-3514** | YP_002308506.1 | 0% | 0% | 0% | 0% | 0% | 0% | 0% |
| **Bat coronavirus RaTG13** | QHR63300.2 | 0% | 0% | 0% | 0% | 0% | 0% | 0% |
| **Bat coronavirus** | QPD89843.1 | 0% | 0% | 0% | 0% | 0% | 0% | 0% |
| **Bovine coronavirus** | CCE89341.1 | 0% | 0% | 0% | 0% | 0% | 0% | 0% |
| **Avian coronavirus** | QIM61640.1 | 0% | 0% | 0% | 0% | 0% | 0% | 0% |


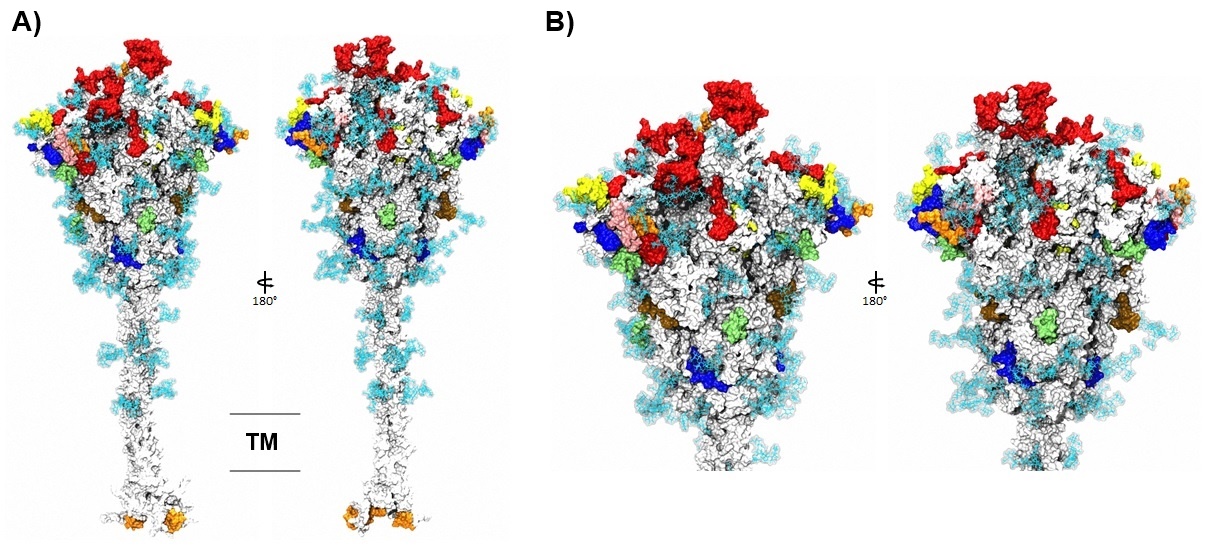


**Supplementary Material 6.- Localization of all selected peptides in the trimeric structure of the spike protein.** (A) The structure of the glycosylated trimeric S protein is shown in the side view and 180-degree view. (B) A zoom of the head of the S protein. In both images, peptides are indicated with color patterns (see Supplementary Material 2), glycans are represented as cyan line surf structures, and the transmembrane viral region is represented by two lines in gray.


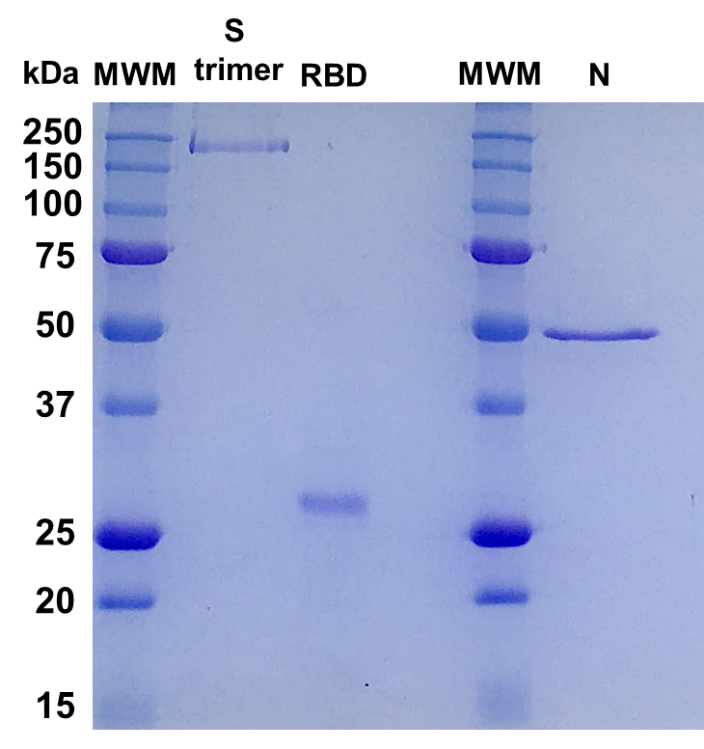


**Supplementary material 7.- Original image of the SDS-PAGE of the used recombinant proteins.** Lane 1: Molecular weight marker (Precision Plus Protein™ Unstained Protein Standards, Biorad Cat #1610363). Lane 2: S trimer (spike glycoprotein). Lane 3: RBD (Receptor binding Domain). Lane 4: space. Lane 5: Molecular weight marker. Lane 6: N (nucleocapsid protein).

**Supplementary Material 8.- Amino acid changes and deletions in SARS-CoV-2 variants of concern.**

| **Variants*** | **Protein S** | **Protein N** |
| --- | --- | --- |
| **Alpha (B.1.1.7)** | H69, V70, Y144, N501Y, A570D, D614G, P681H, T716I, S982A, D1118H | D3L, R203K, G204R, S235F |
| **Beta (B.1.351)** | D80A, D215G, L241-243, K417N, E484, N501Y, D614G, A701V | T205I |
| **Gamma (P.1)** | L18F, T20N, P26S, D138Y, R190S, K417T, E484K, N501Y, D614G, H655Y, T1027I, V1176F | P80R, R203K, G204R |
| **Delta (B.1.617.2)** | T19R, E156, F157, R158G, L452R, T478K, D614G, P681R, D950N | D63G, R203M, D377Y |
| **Omicron (BA.1)** | A67V, H69, V70, T95I, G142-Y144, Y145D, N211, L212I, G339D, S371L, S373P, S375F, K417N, N440K, G446S, S477N, T478K, E484A, Q493R, G496S, Q498R, N501Y, Y505H, T547K, D614G, H655Y, N679K, P681H, N764K, D796Y, N856K, Q954H, N969K, L981F | P13L, E31-S33, R203K, G204R |

***Source:** CoVariants (<https://covariants.org/variants>). : Deletion.
