# Supplementary material for: Utility of in silico-identified-peptides in spike-S1 domain and nucleocapsid of SARS-CoV-2 for antibody detection in COVID-19 patients and antibody production
Source: Sci Rep. 2022 Sep 5;12:15057. doi: 10.1038/s41598-022-18517-w (PMC9442563; doi:10.1038/s41598-022-18517-w)

**Supplementary material 3.- Protein BLAST and conservation analysis of the selected peptide.**

**Protein BLAST**

**NT-1**


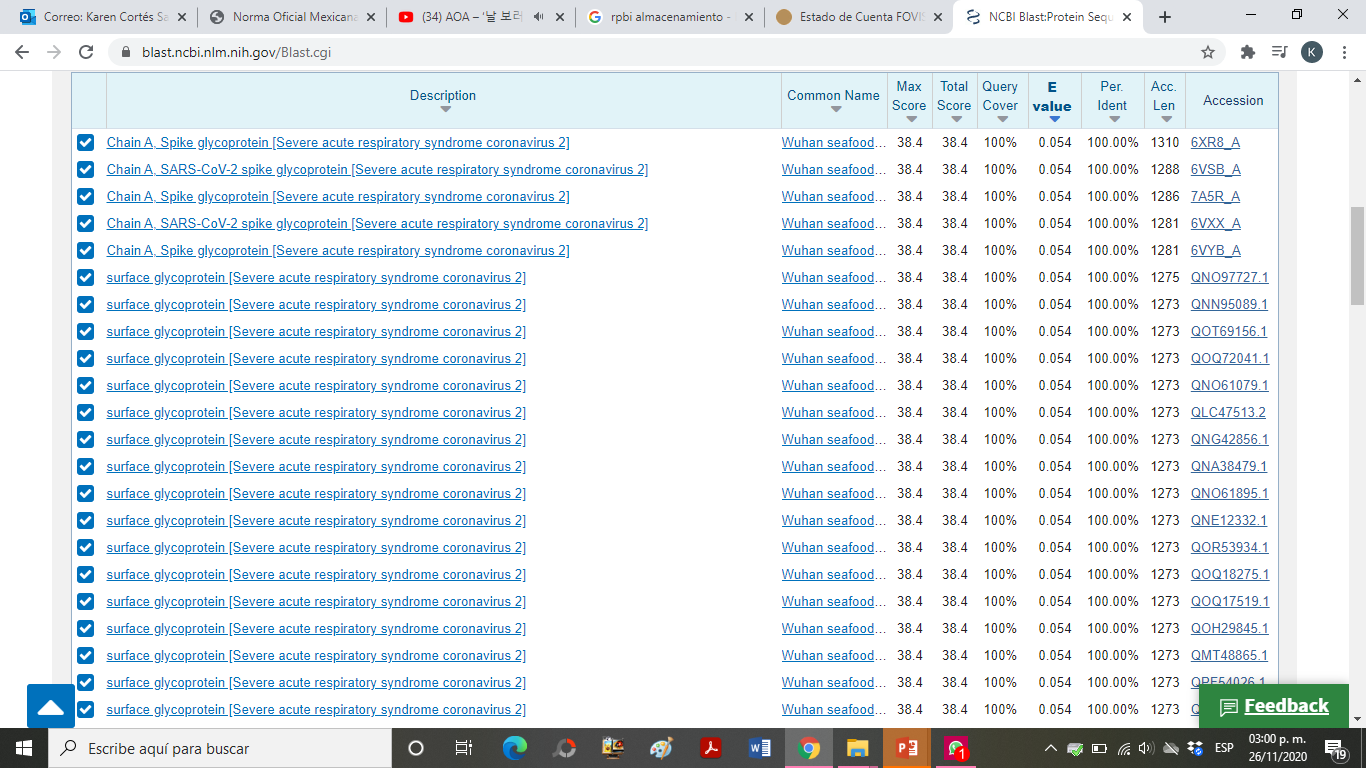


**NT-2**


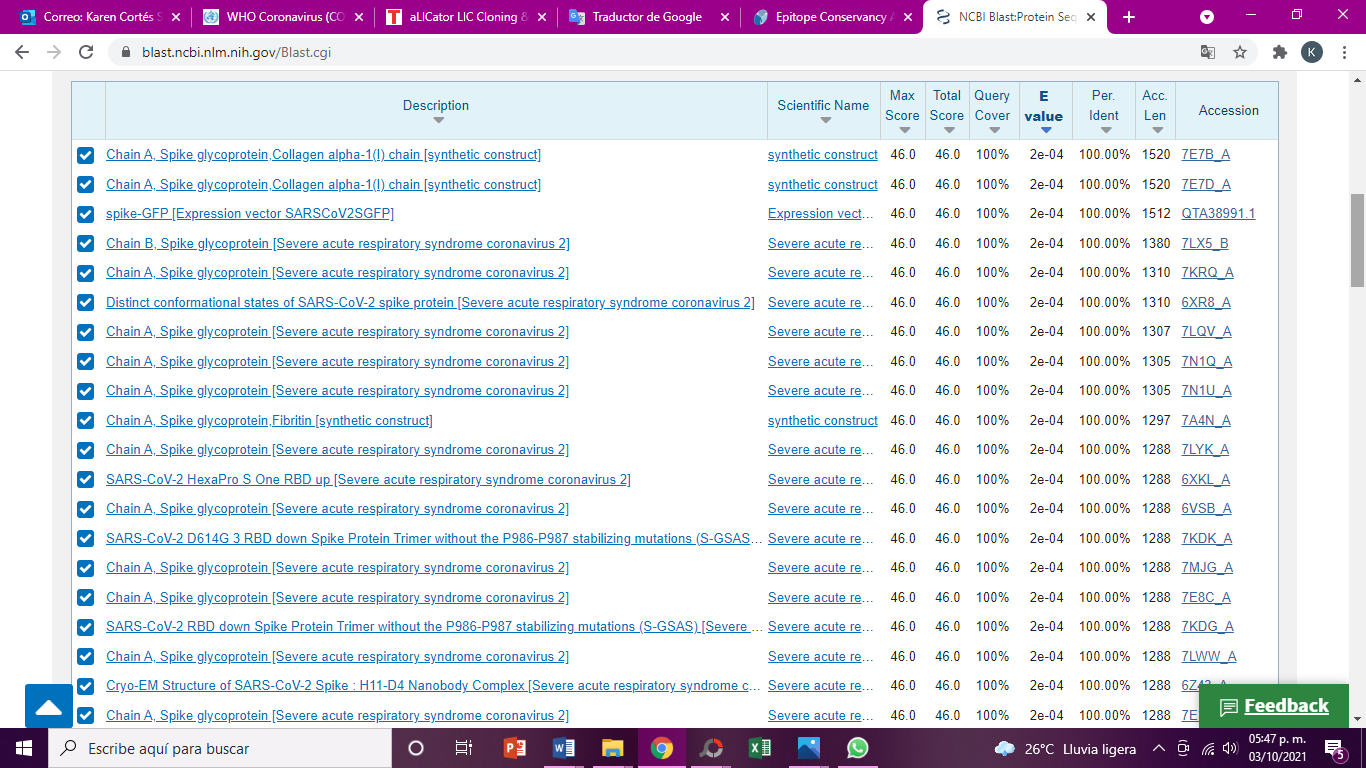


**RBD1**


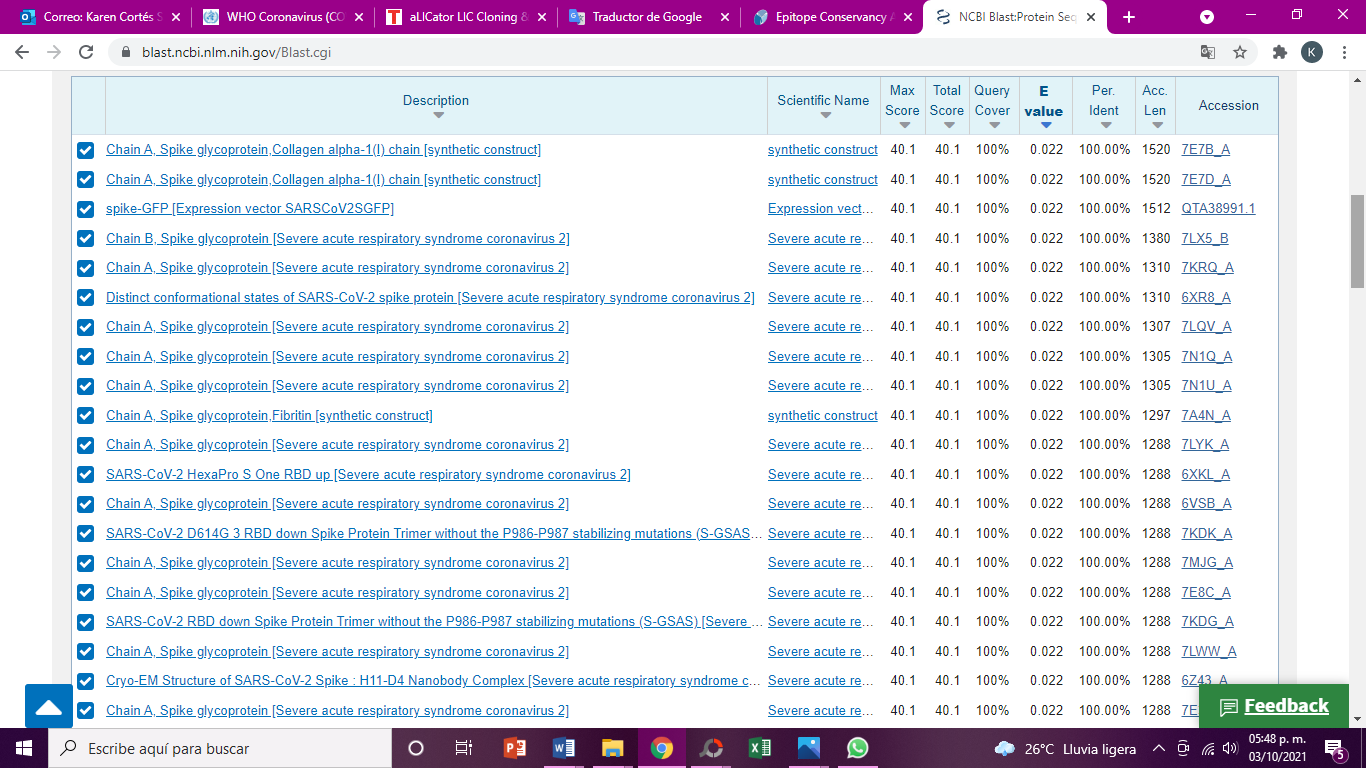


**RBD2**


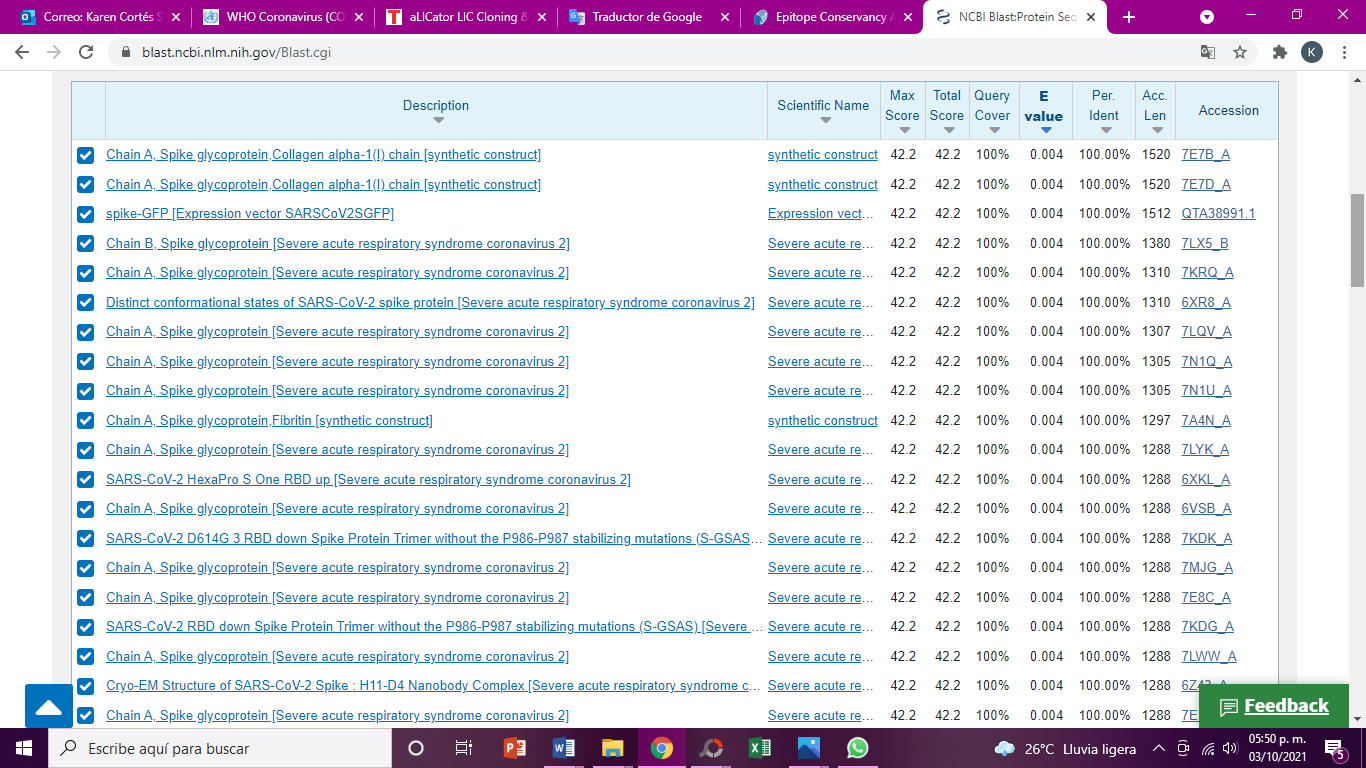


**RBD3**


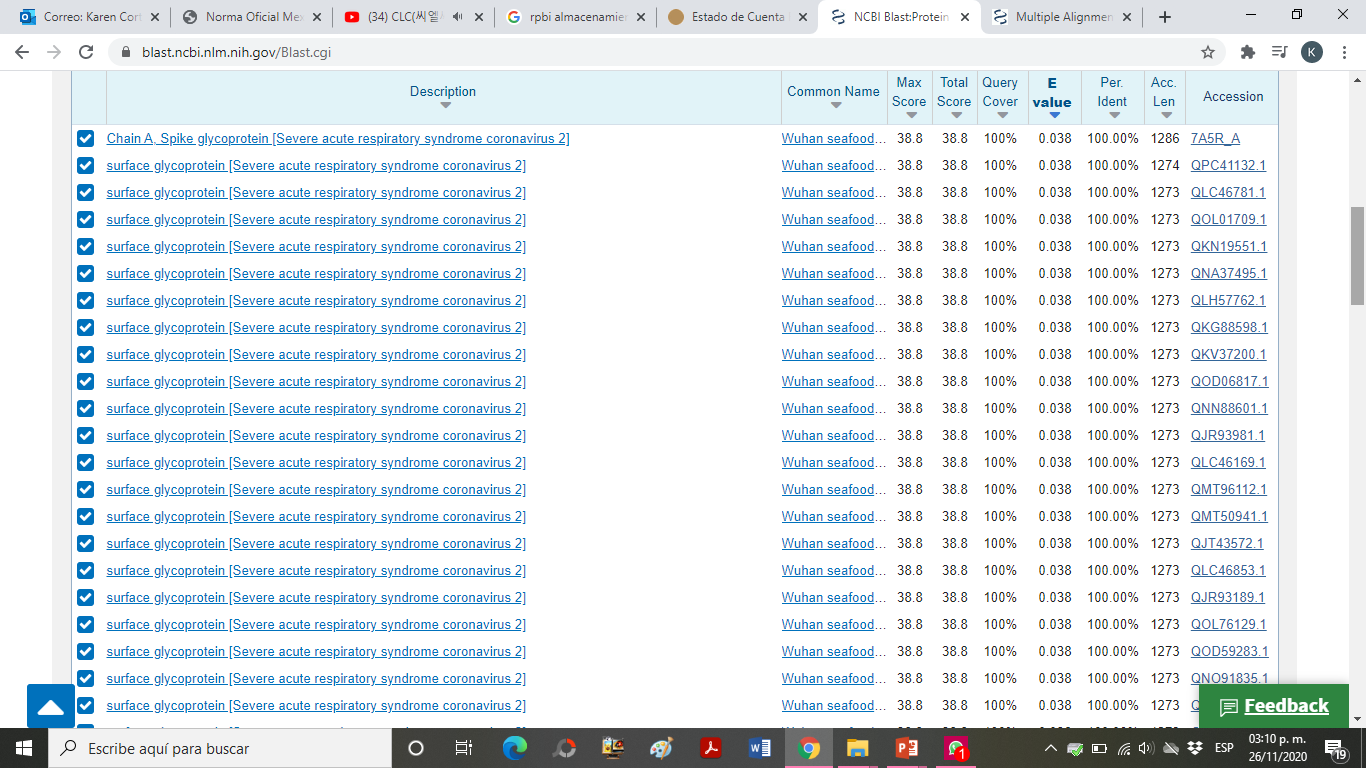


**RBD4**


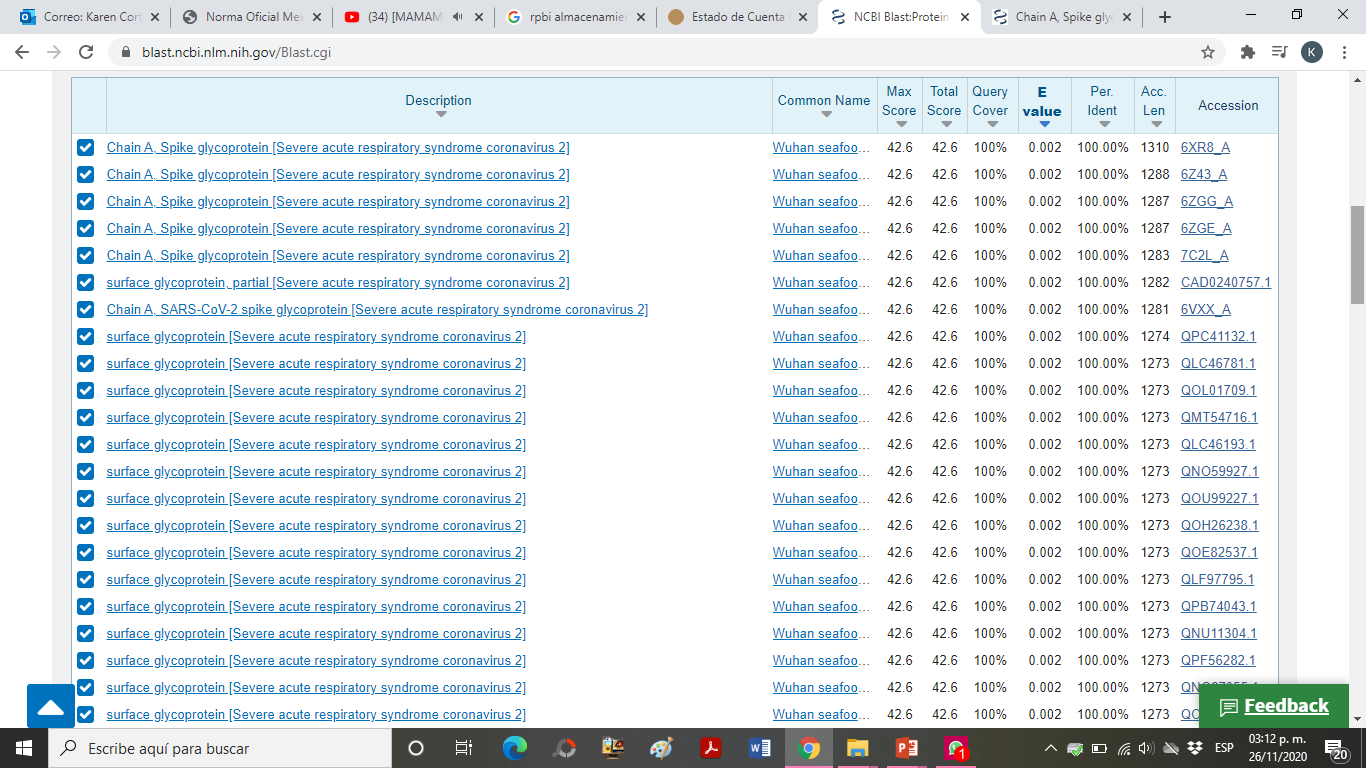


**RBD5**


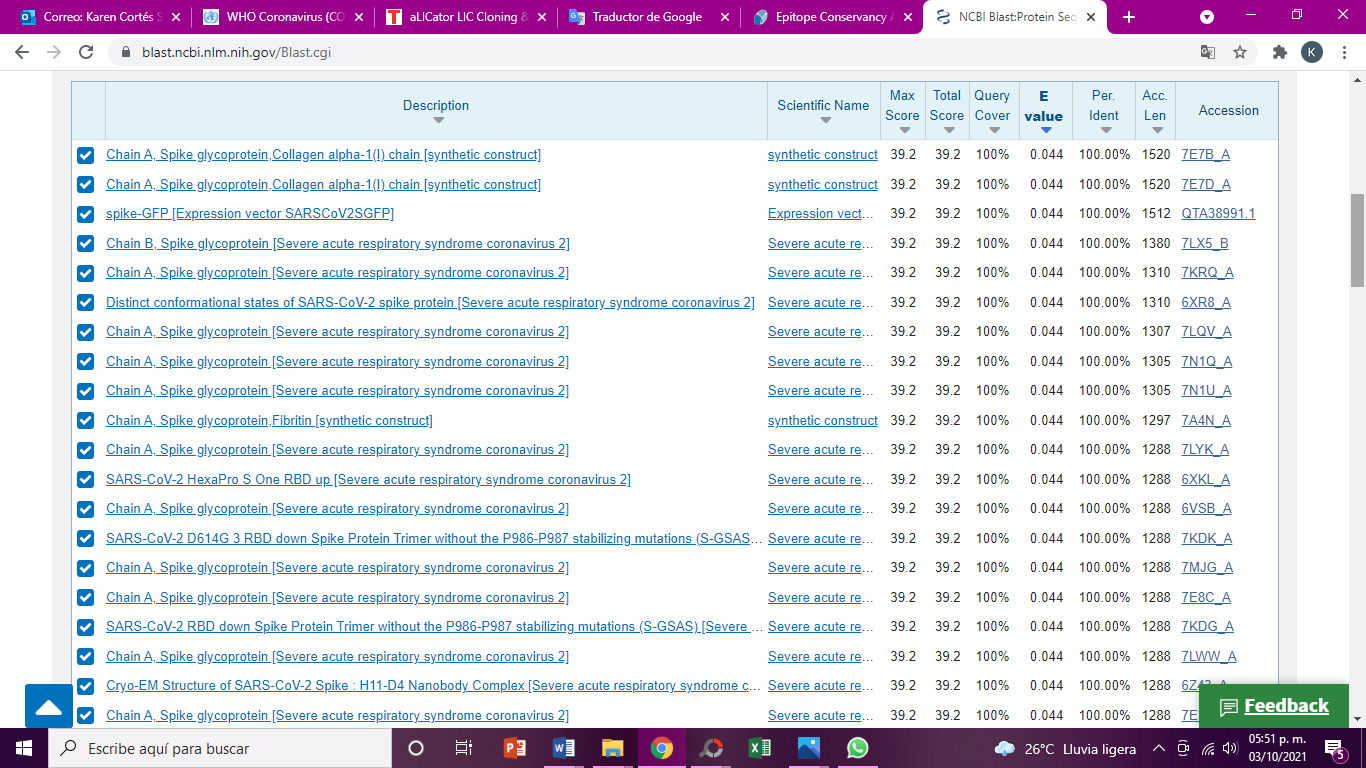


**N**


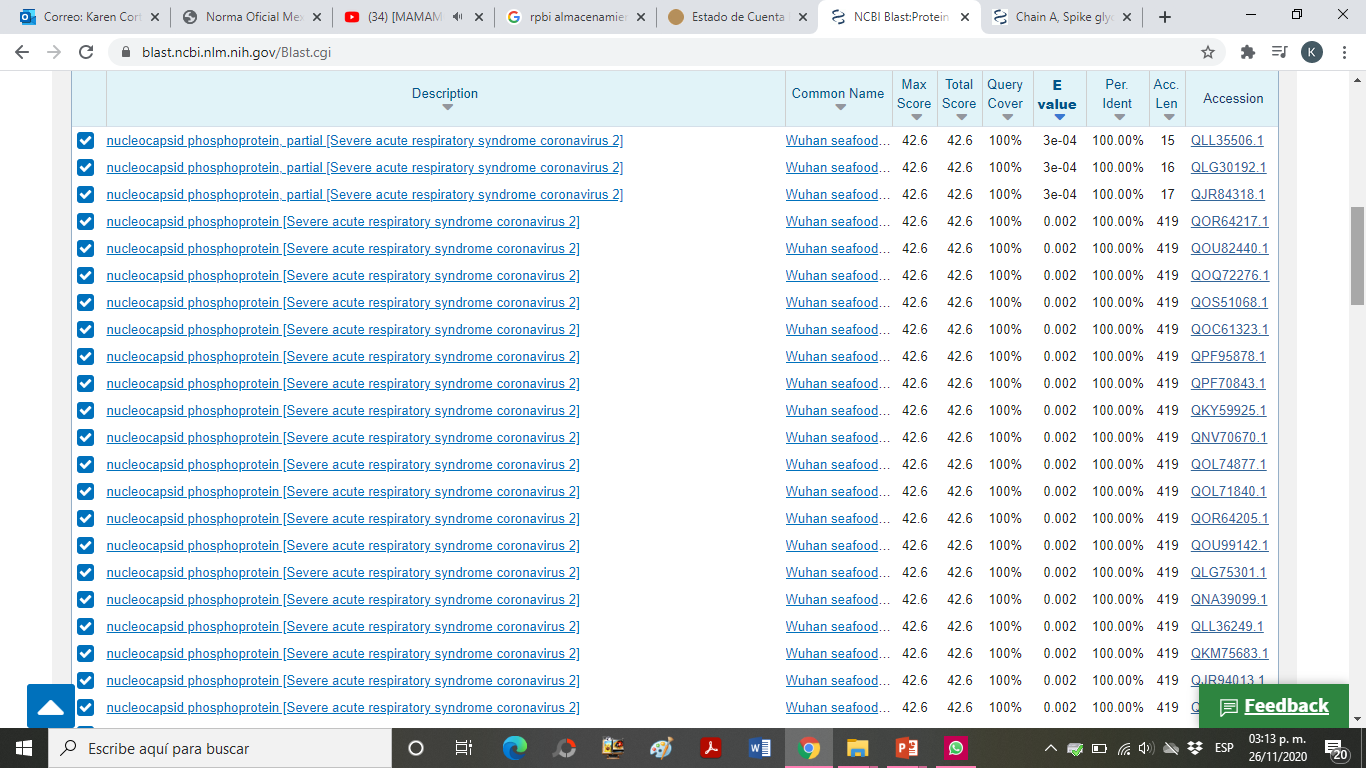


**Epitope conservation analysis**

**NT-1**


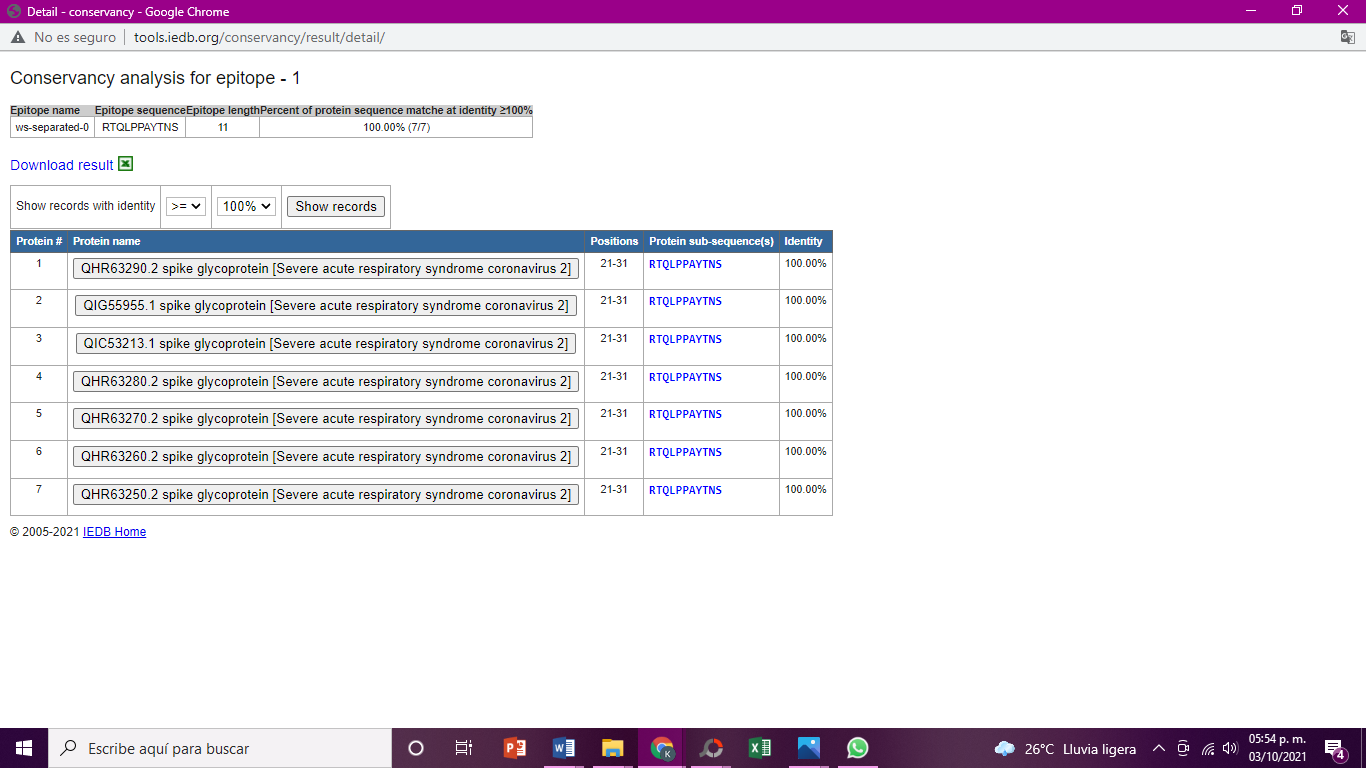


**NT-2**


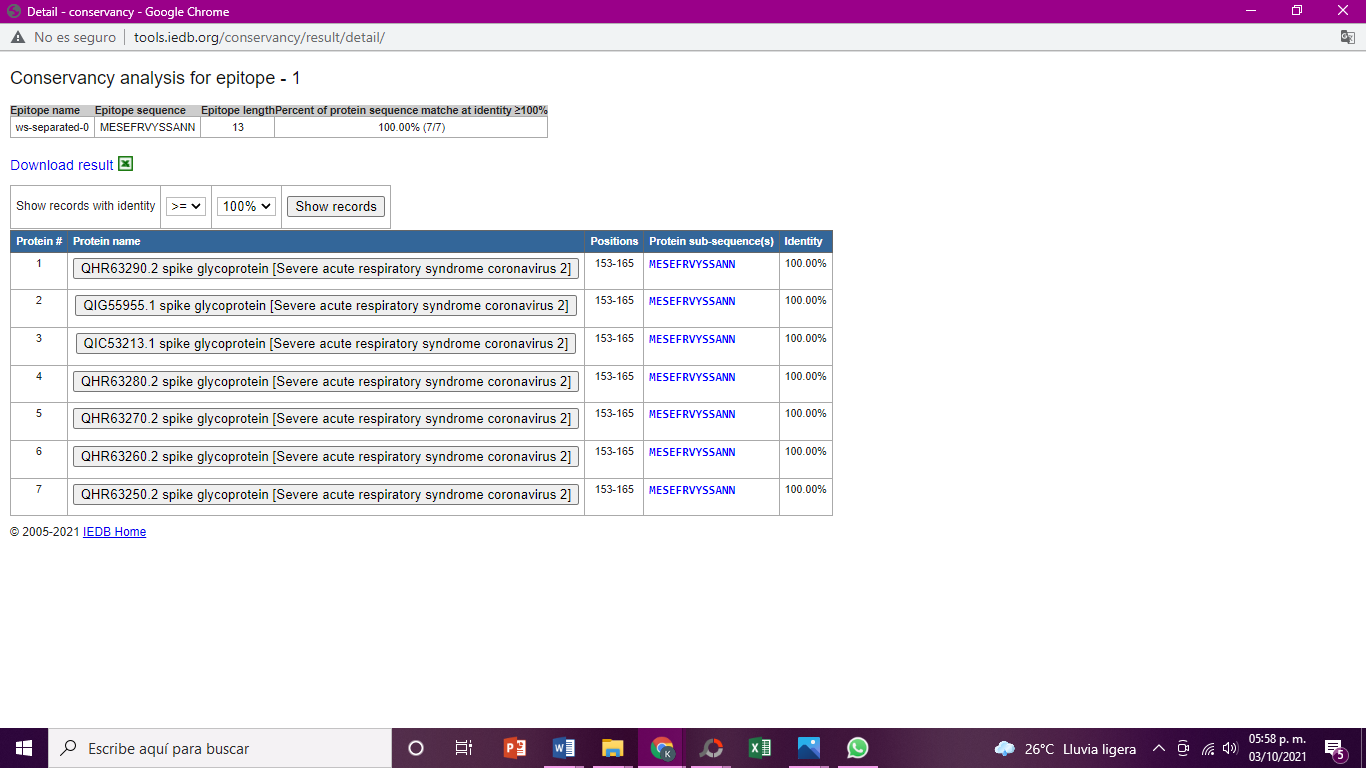


**RBD1**


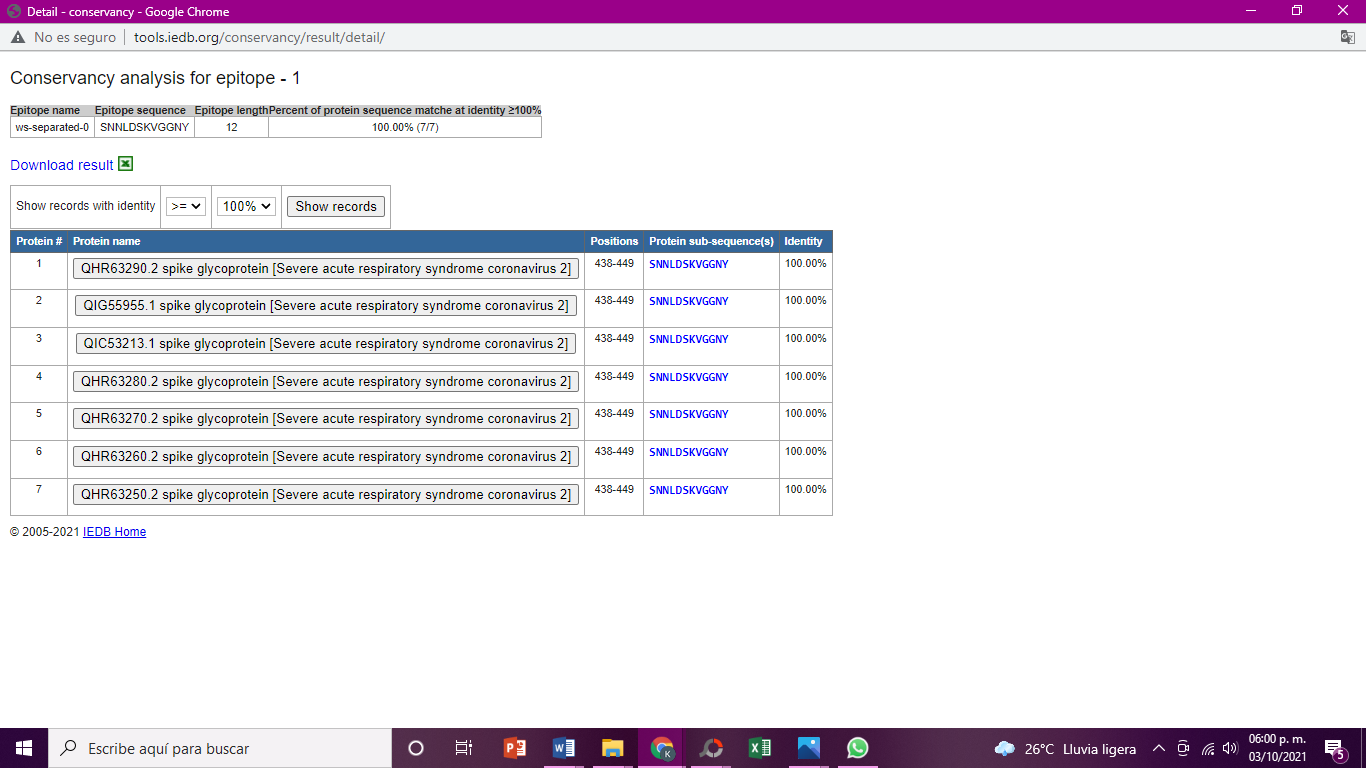


**RBD2**


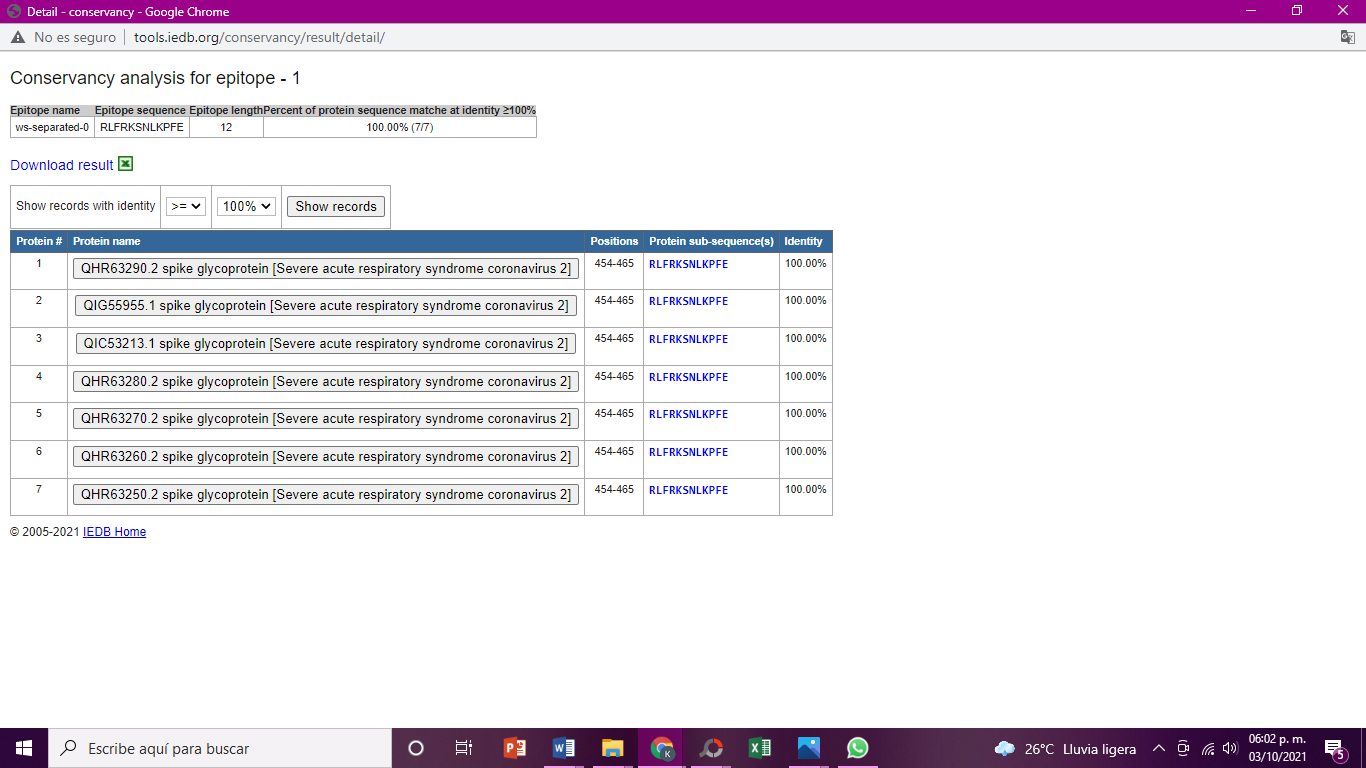


**RBD3**


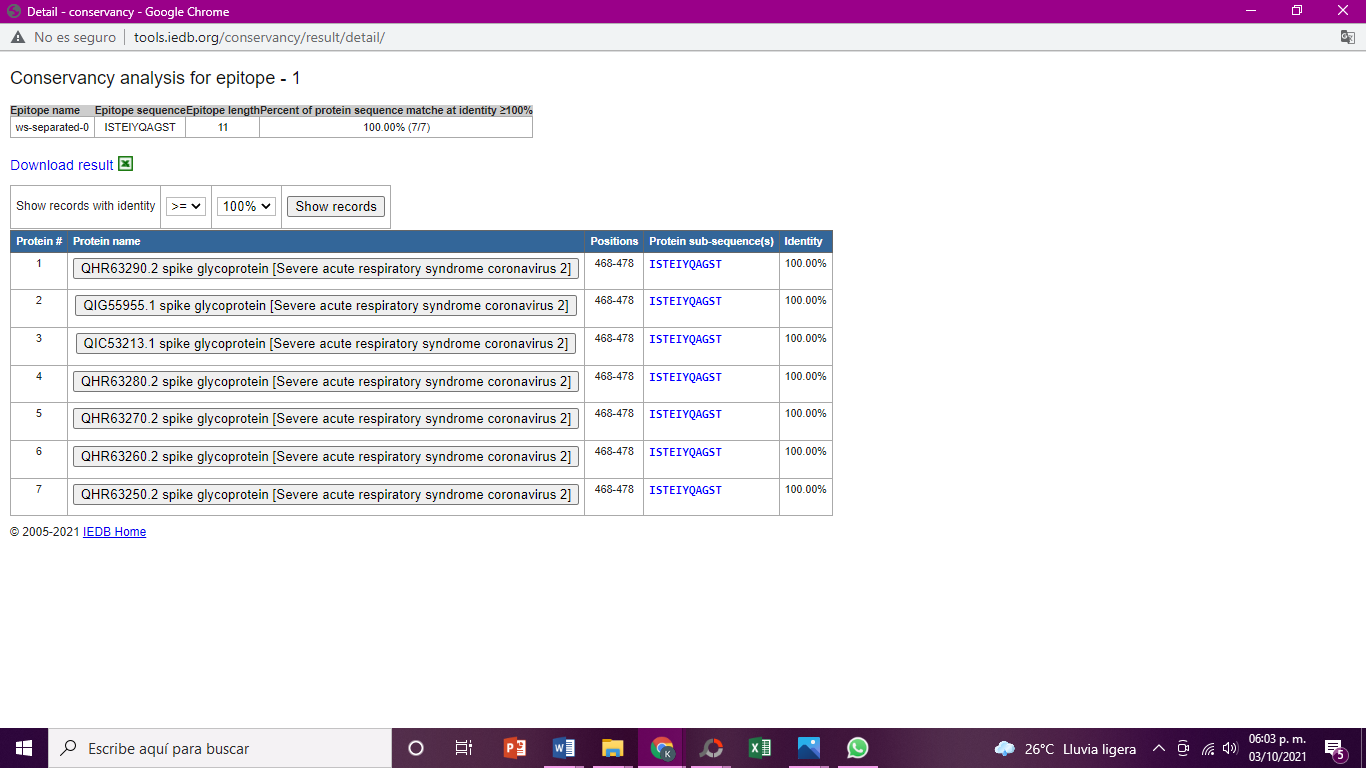


**RBD4**


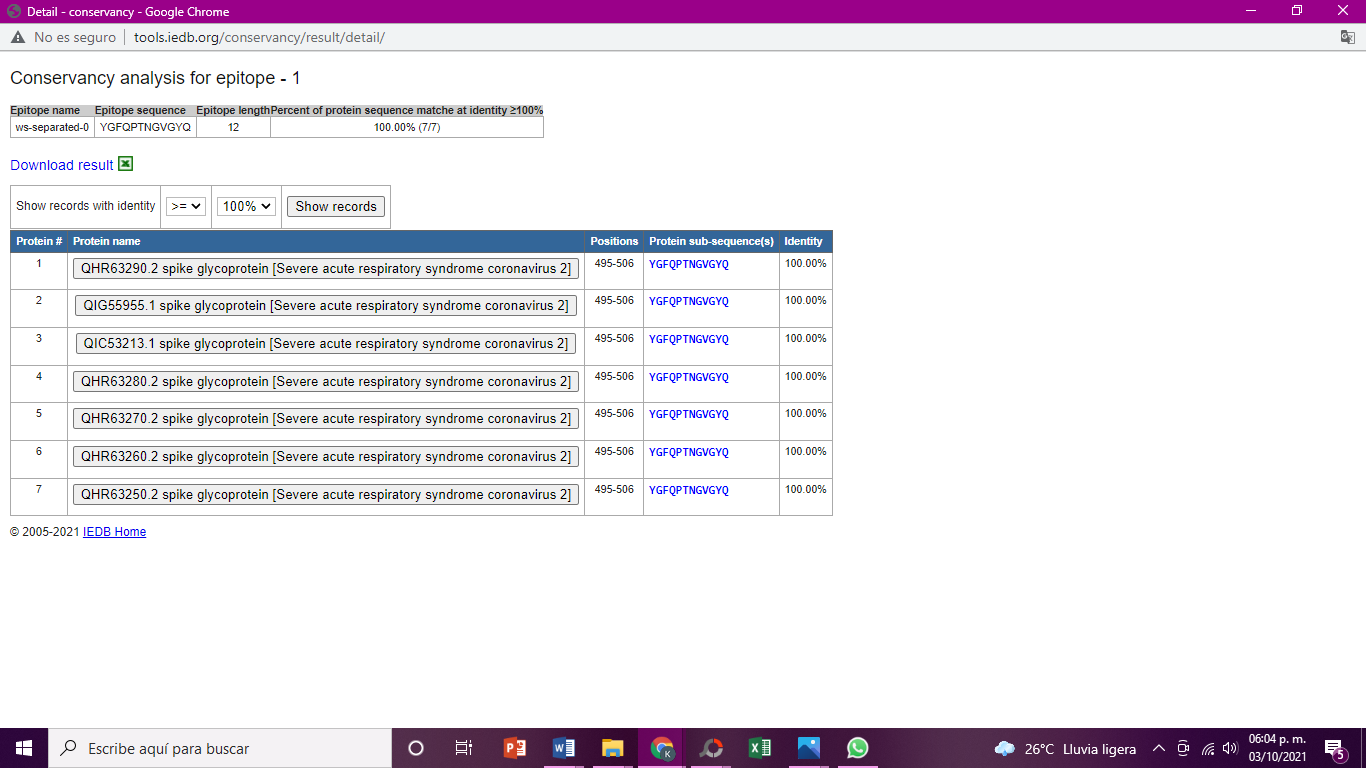


**RBD5**


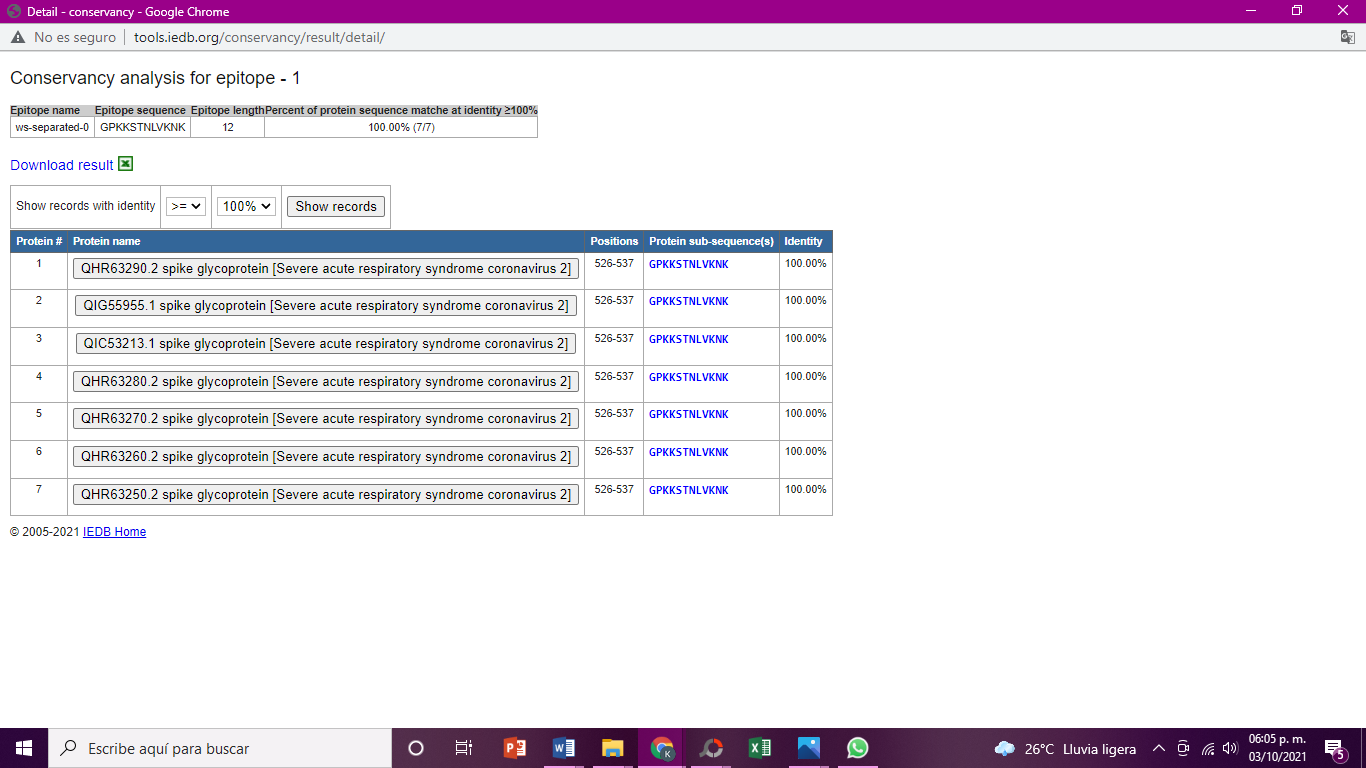


**N**


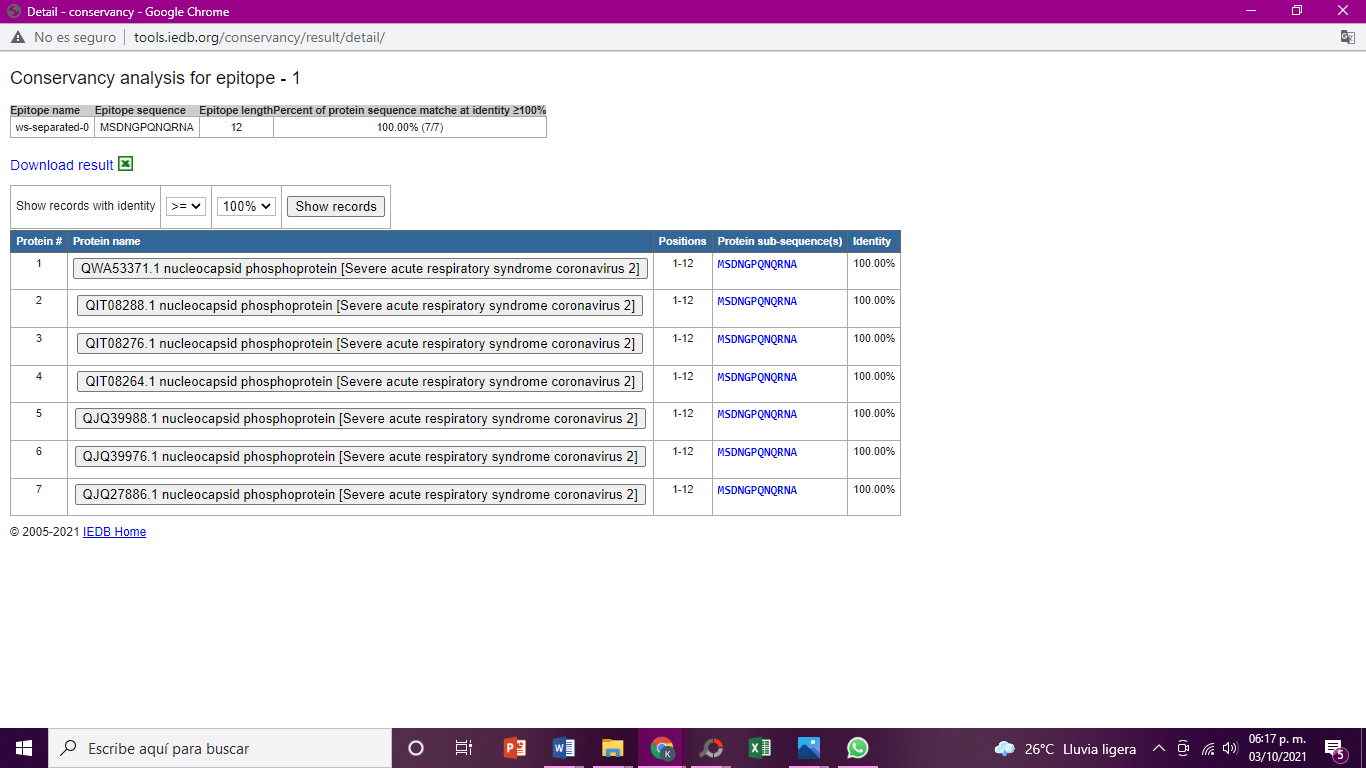

Supplement: Supplementary file 3 — Supplementary Information 3. [file 41598_2022_18517_MOESM3_ESM.docx]
