# Supplementary material for: Utility of in silico-identified-peptides in spike-S1 domain and nucleocapsid of SARS-CoV-2 for antibody detection in COVID-19 patients and antibody production
Source: Sci Rep. 2022 Sep 5;12:15057. doi: 10.1038/s41598-022-18517-w (PMC9442563; doi:10.1038/s41598-022-18517-w)

**Supplementary material 5.- Conservancy analysis of the peptides and other coronavirus-related virus.**

**SARS coronavirus Tor2 (Access number: YP_009825051.1)**


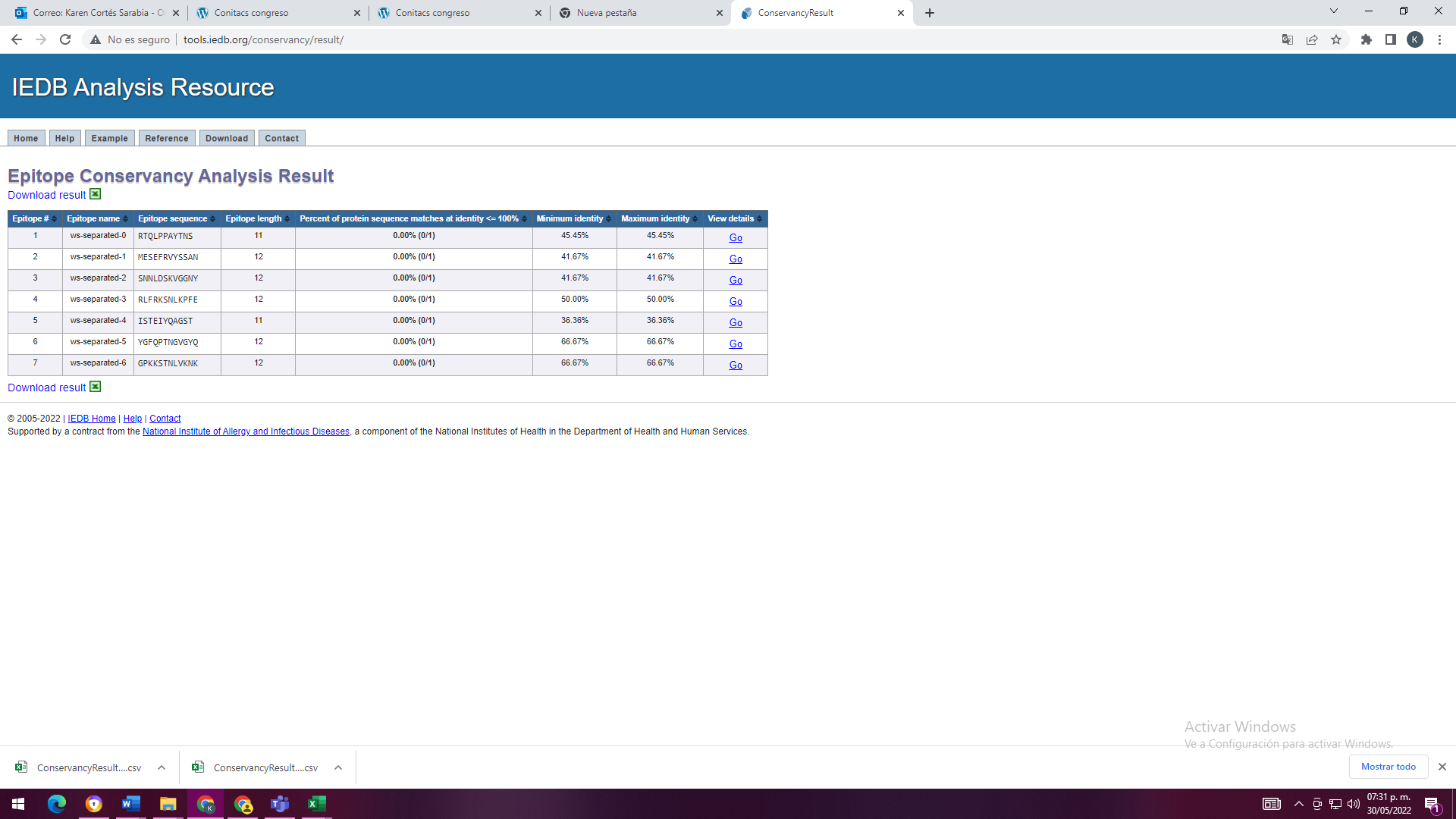


**Severe acute respiratory syndrome coronavirus 2 (Access number: YP_009724390.1)**


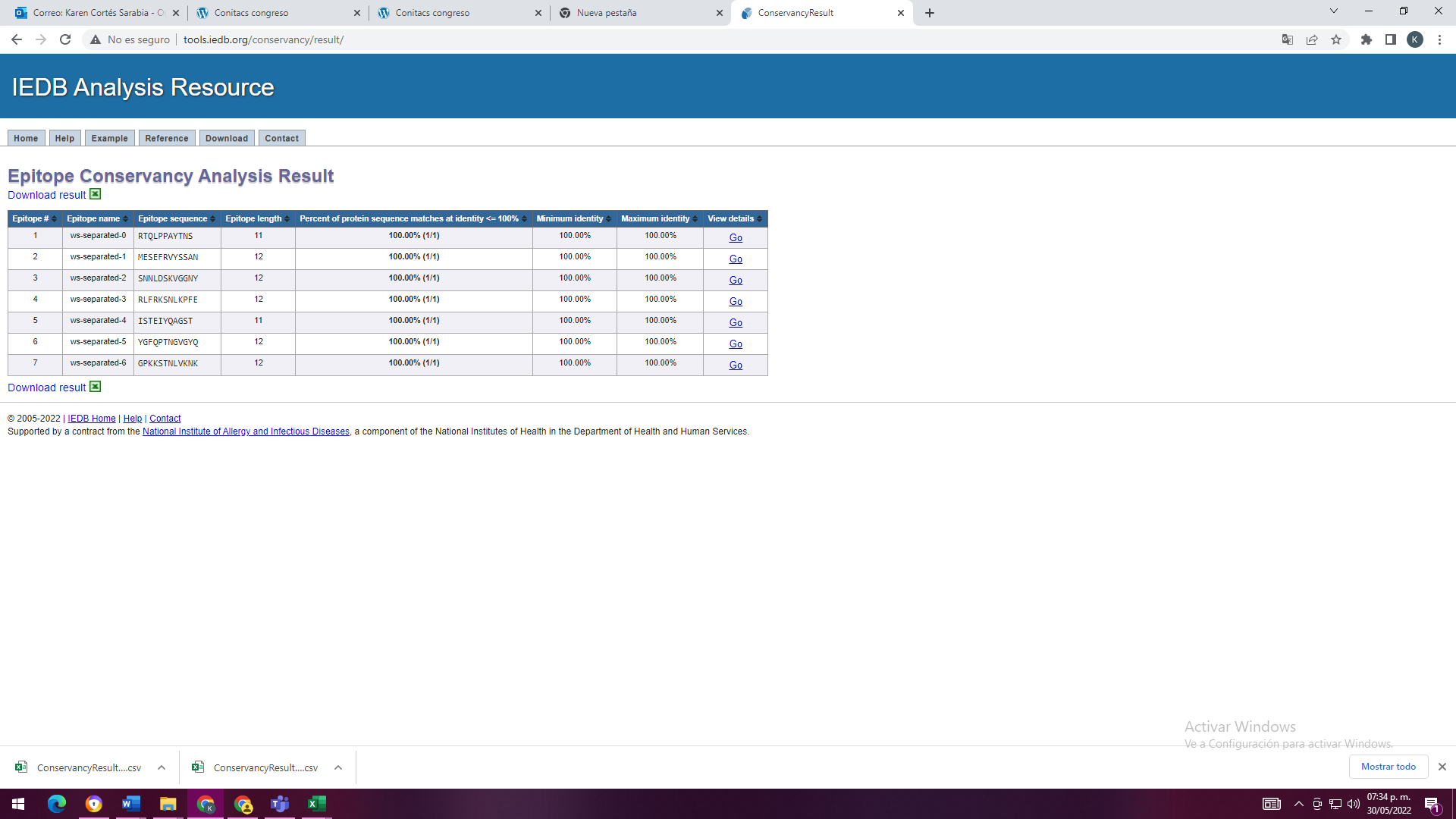


**Middle East respiratory syndrome-related coronavirus (Access number: YP_009047204.1)**


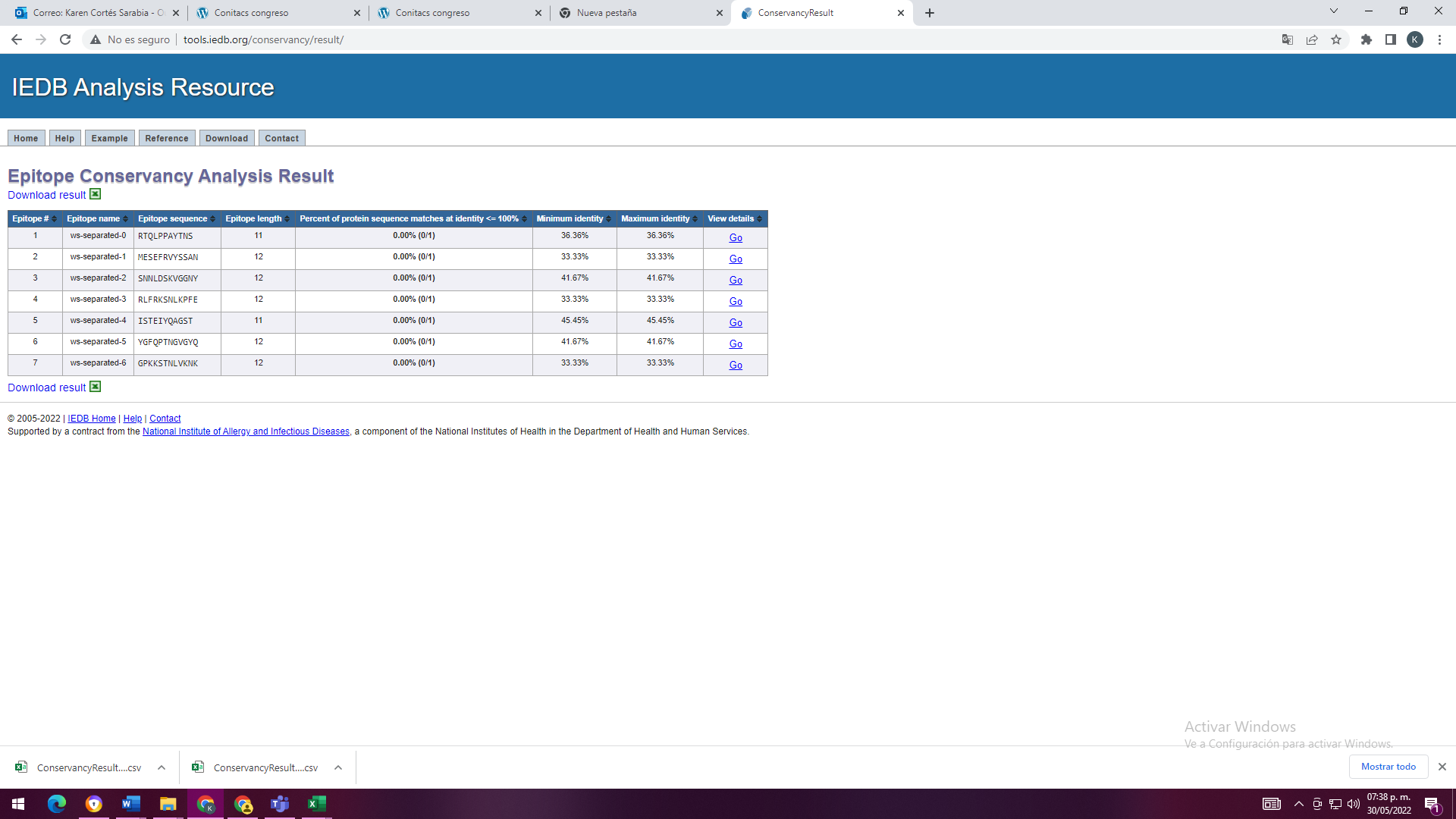


**Middle East respiratory syndrome-related coronavirus (Access number: AHX00731.1)**


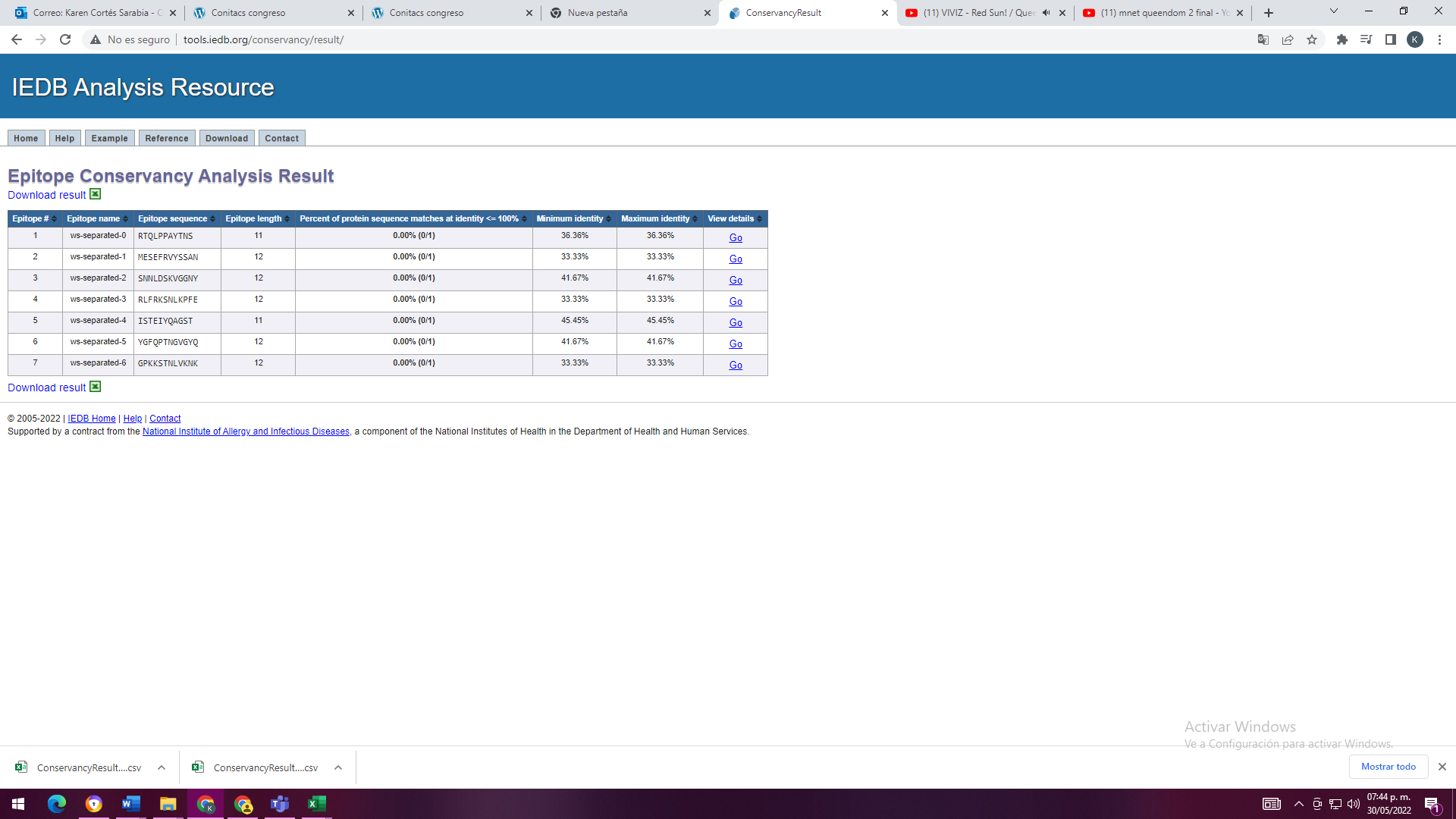


**Human betacoronavirus 2c England-Qatar/2012 (Access number: AGG22542.1)**


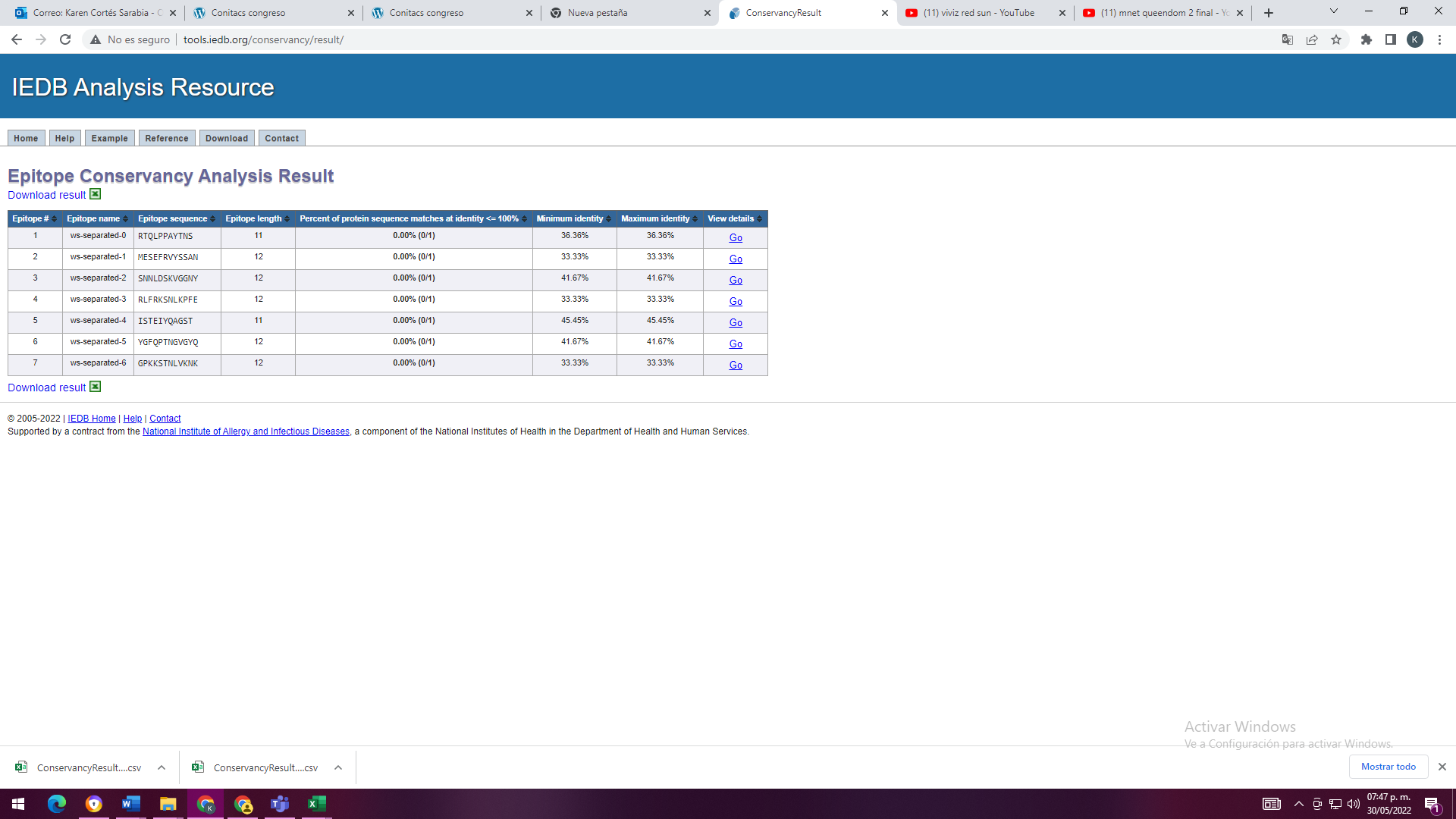


**Human coronavirus NL63 (Access number: YP_003767.1)**


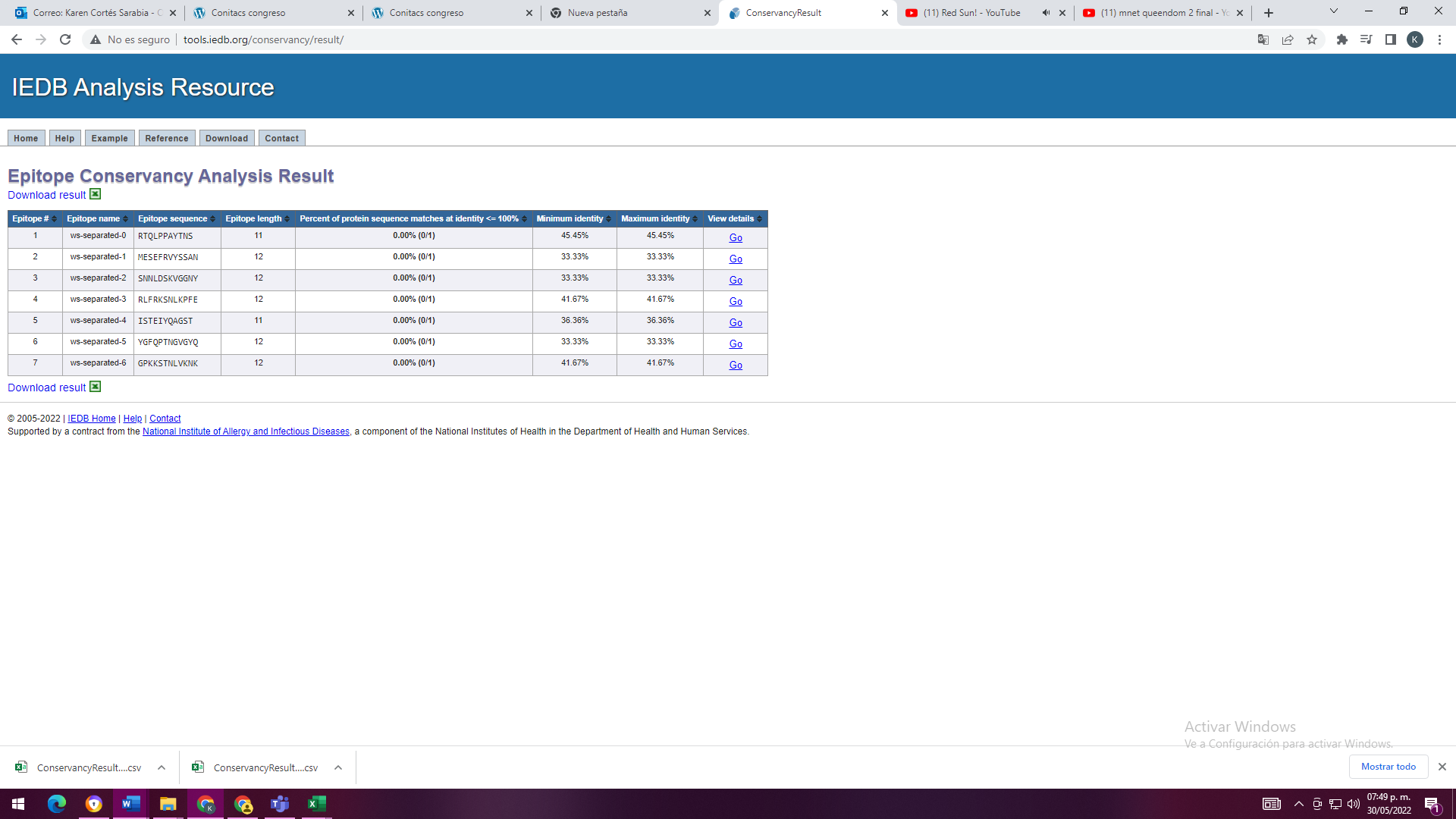


**Human coronavirus 229E (Access number: NP_073551.1)**


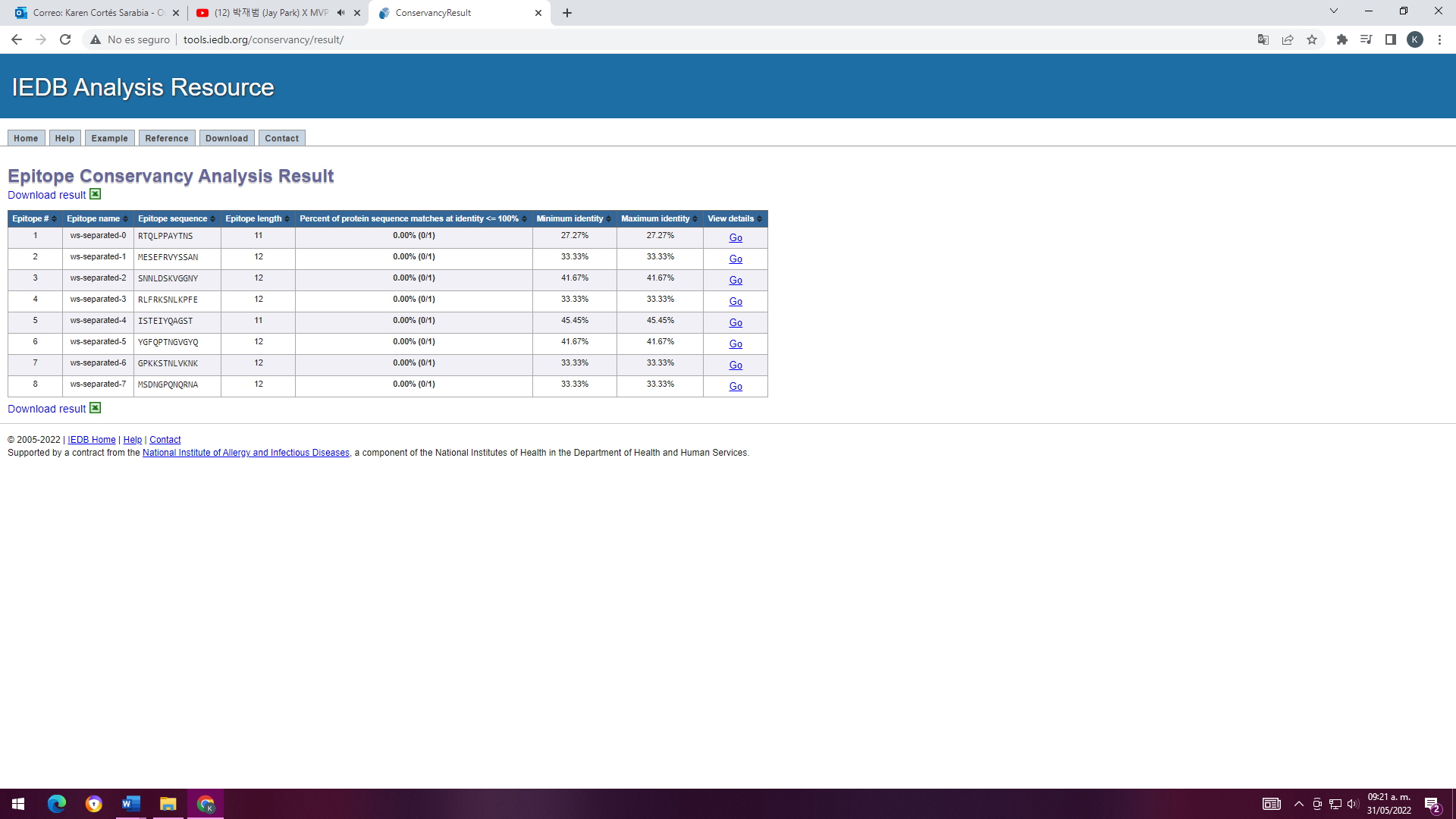


**Human coronavirus 229E (Access number: ABB90529.1)**


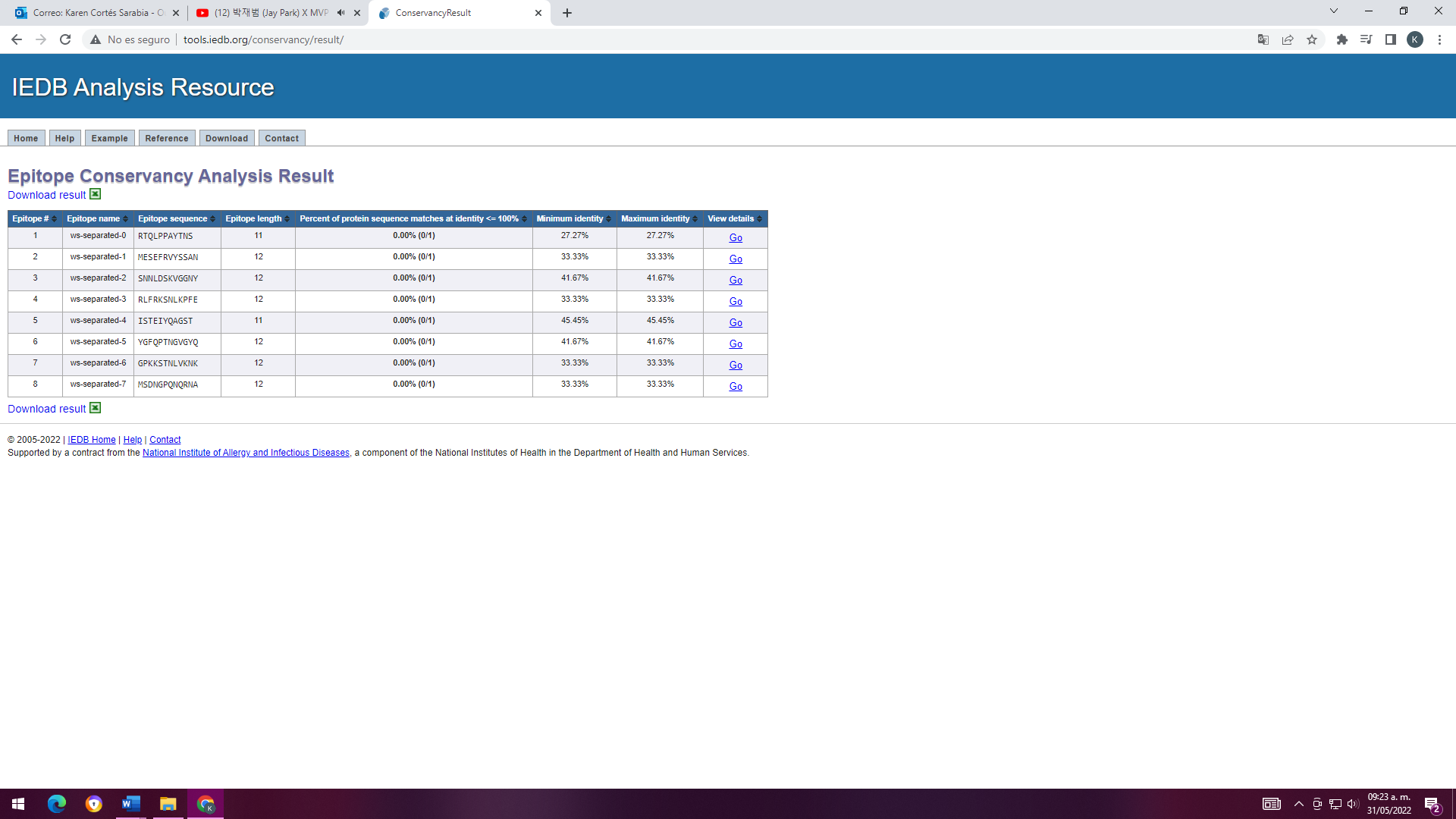


**Human coronavirus HKU1 (Access number: YP_173238.1)**


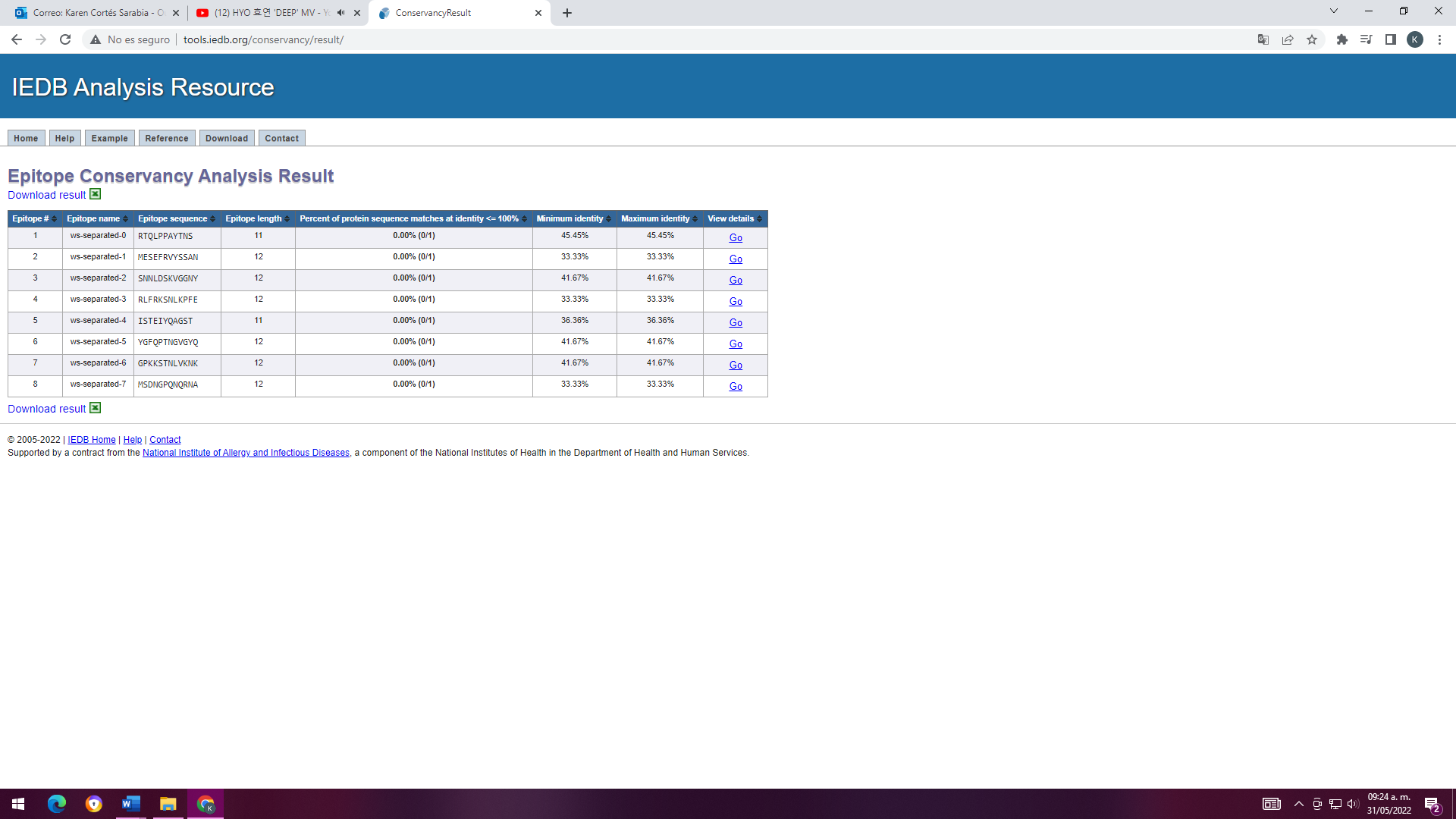


**Human coronavirus HKU1 (Access number: ADN03339.1)**


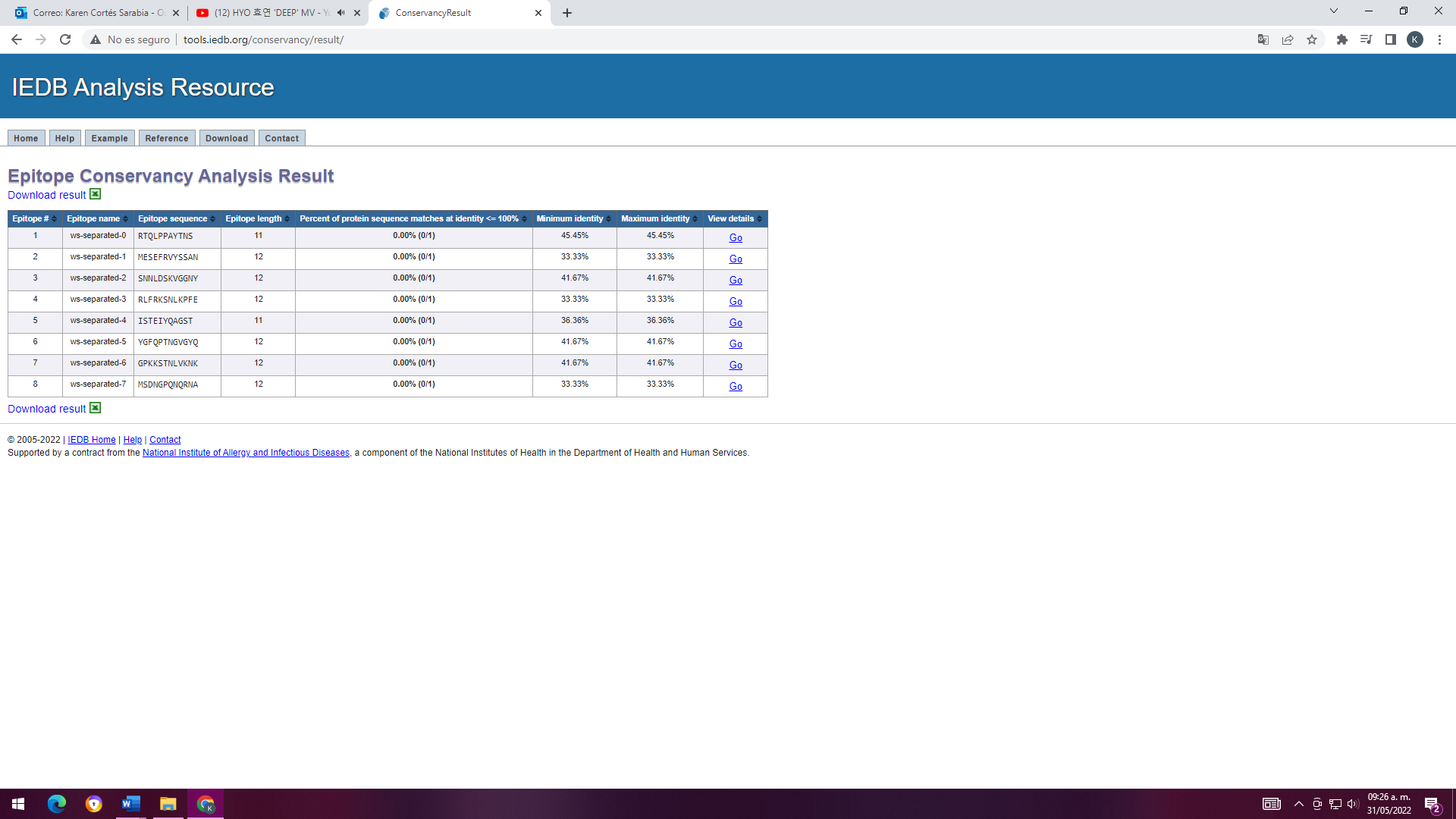


**Human coronavirus OC43 (Access number: YP_009555241.1)**


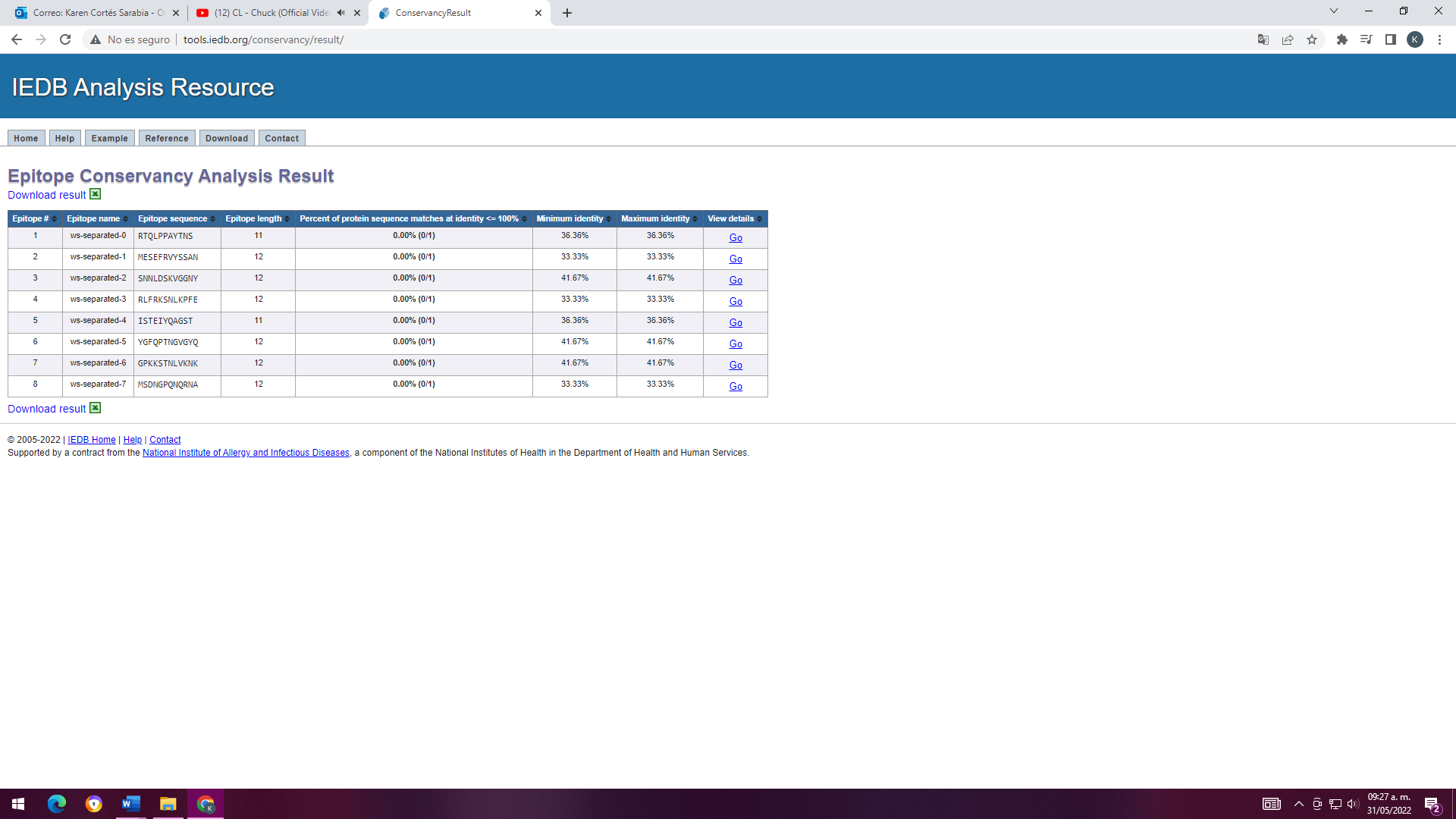


**Animals**

**Miniopterus bat coronavirus HKU8 (Access number: YP_001718612.1)**


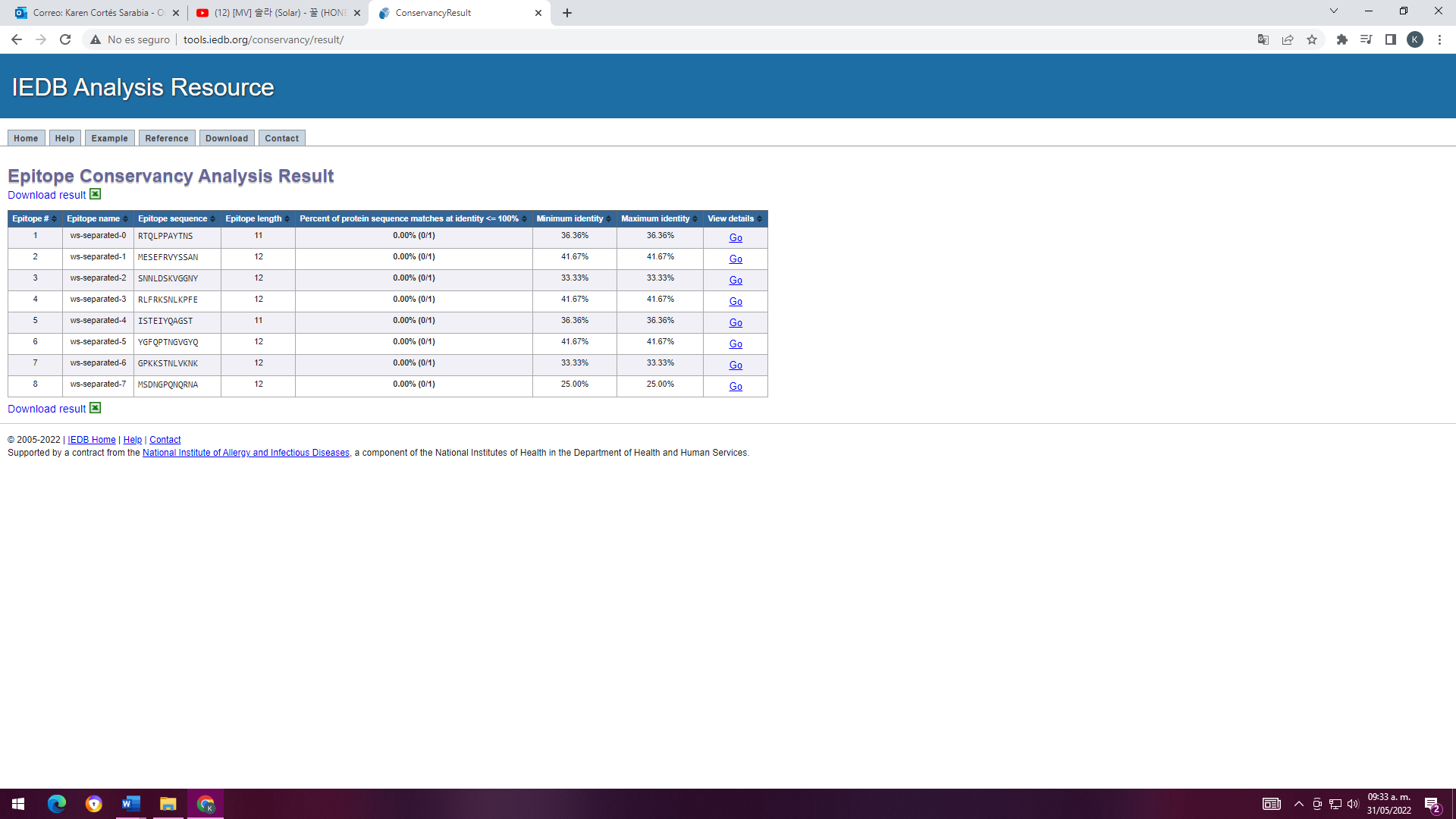


**Rousettus bat coronavirus HKU9 (Access number: YP_001039971.1)**


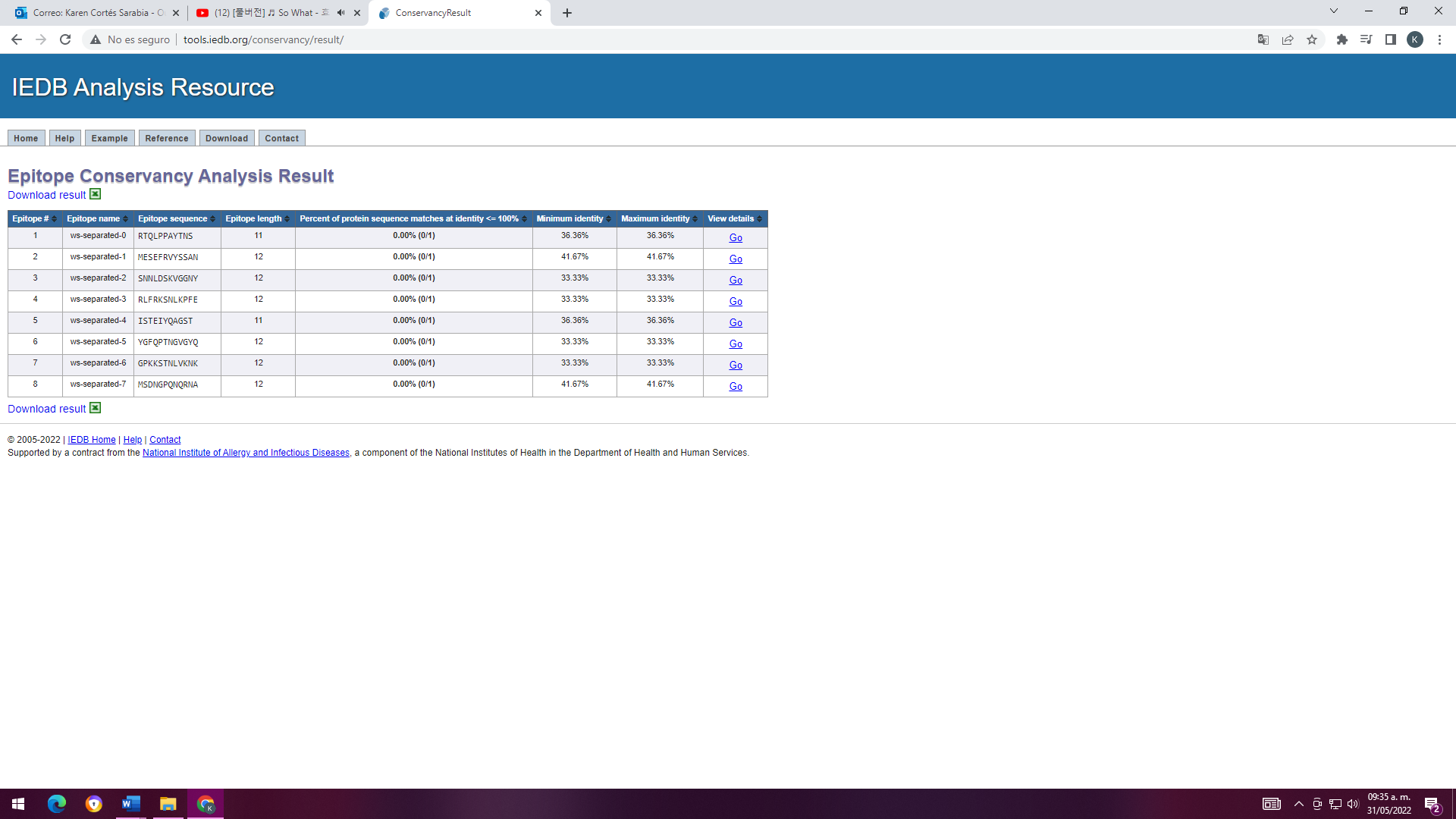


**Beluga whale coronavirus SW1 (Access number: YP_001876437.1)**


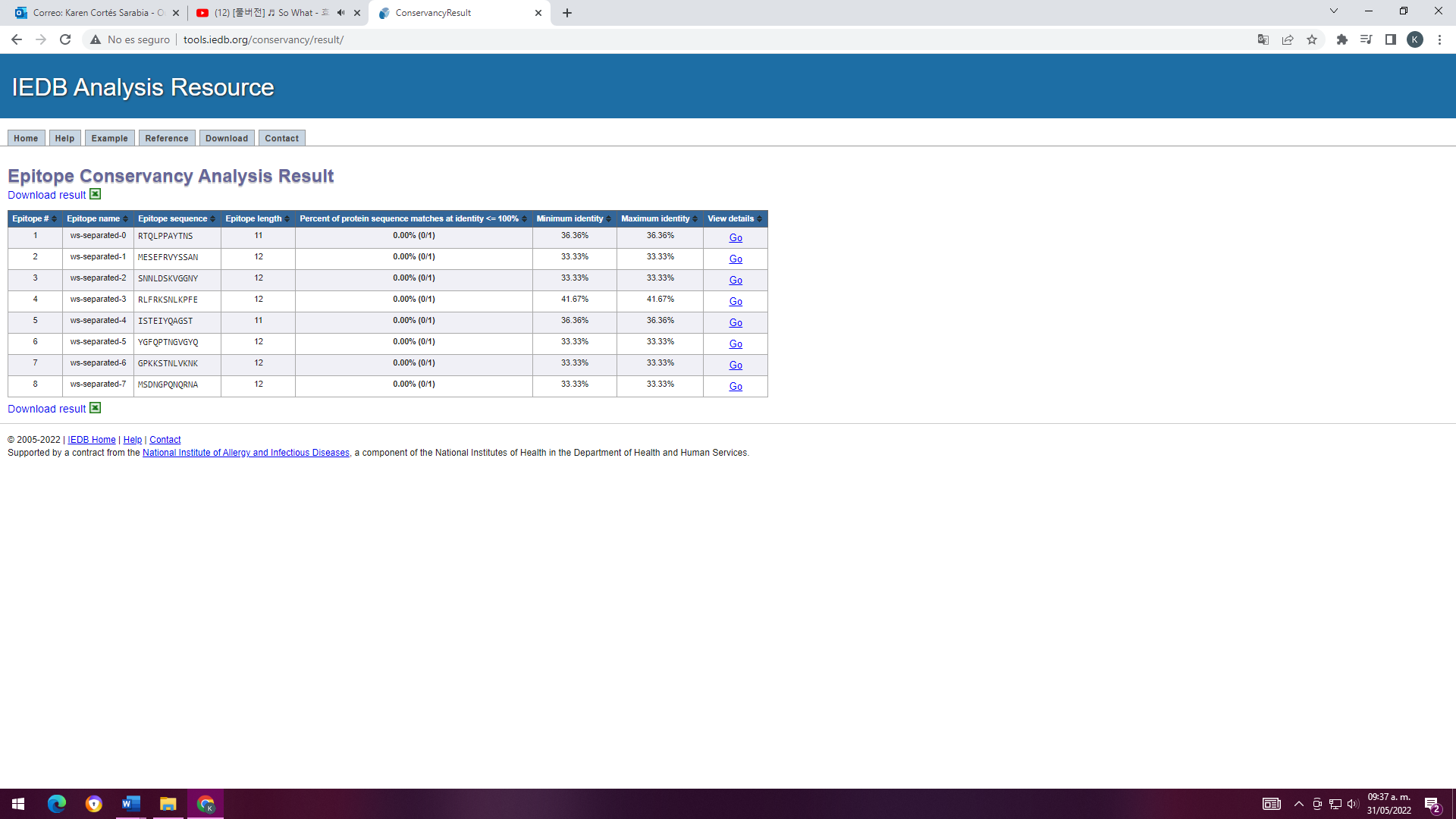


**Bottlenose dolphin coronavirus HKU22 (Access number: AHB63508.1)**


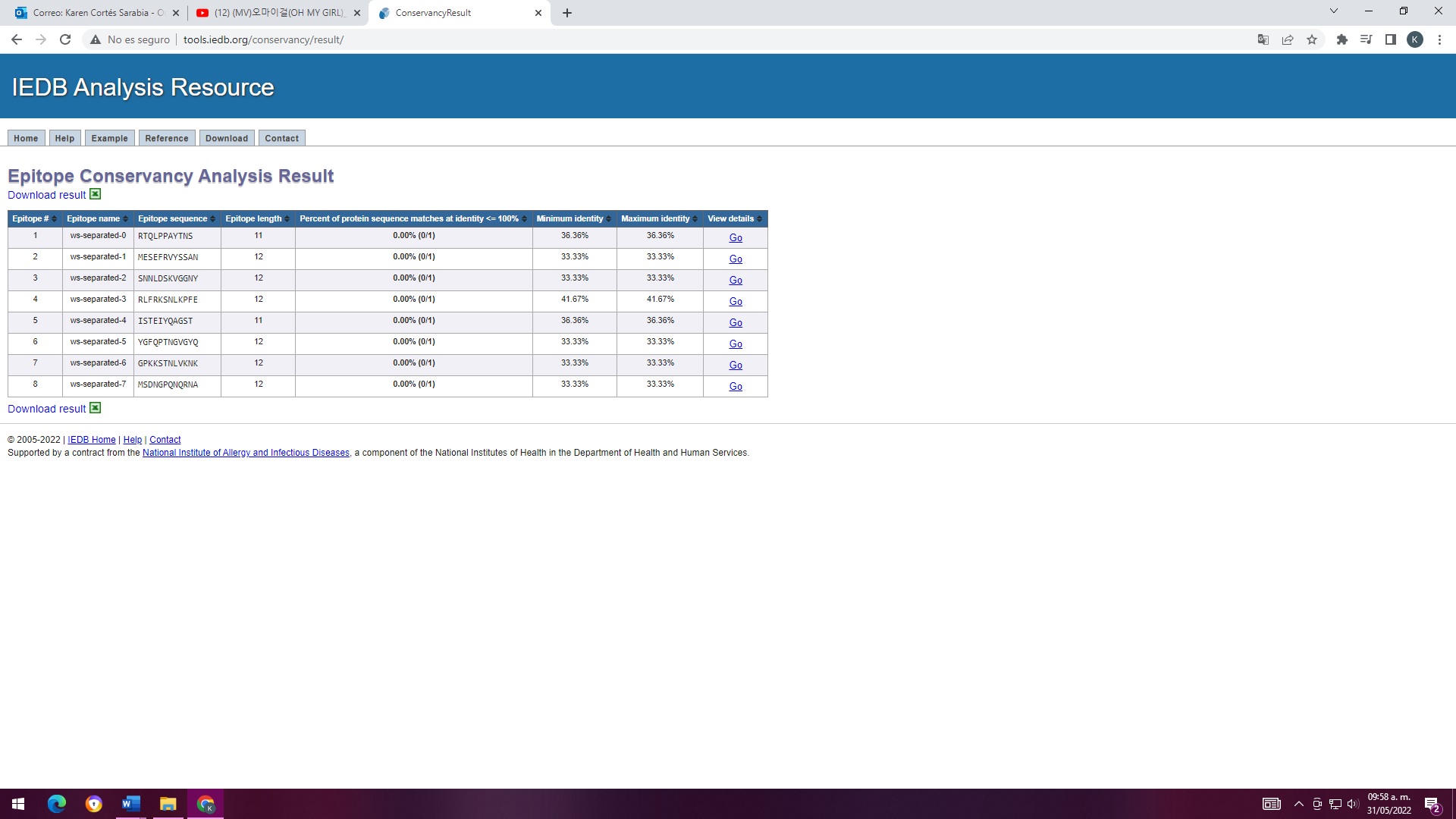


**Night heron coronavirus HKU19 (Access number: AFD29226.1)**


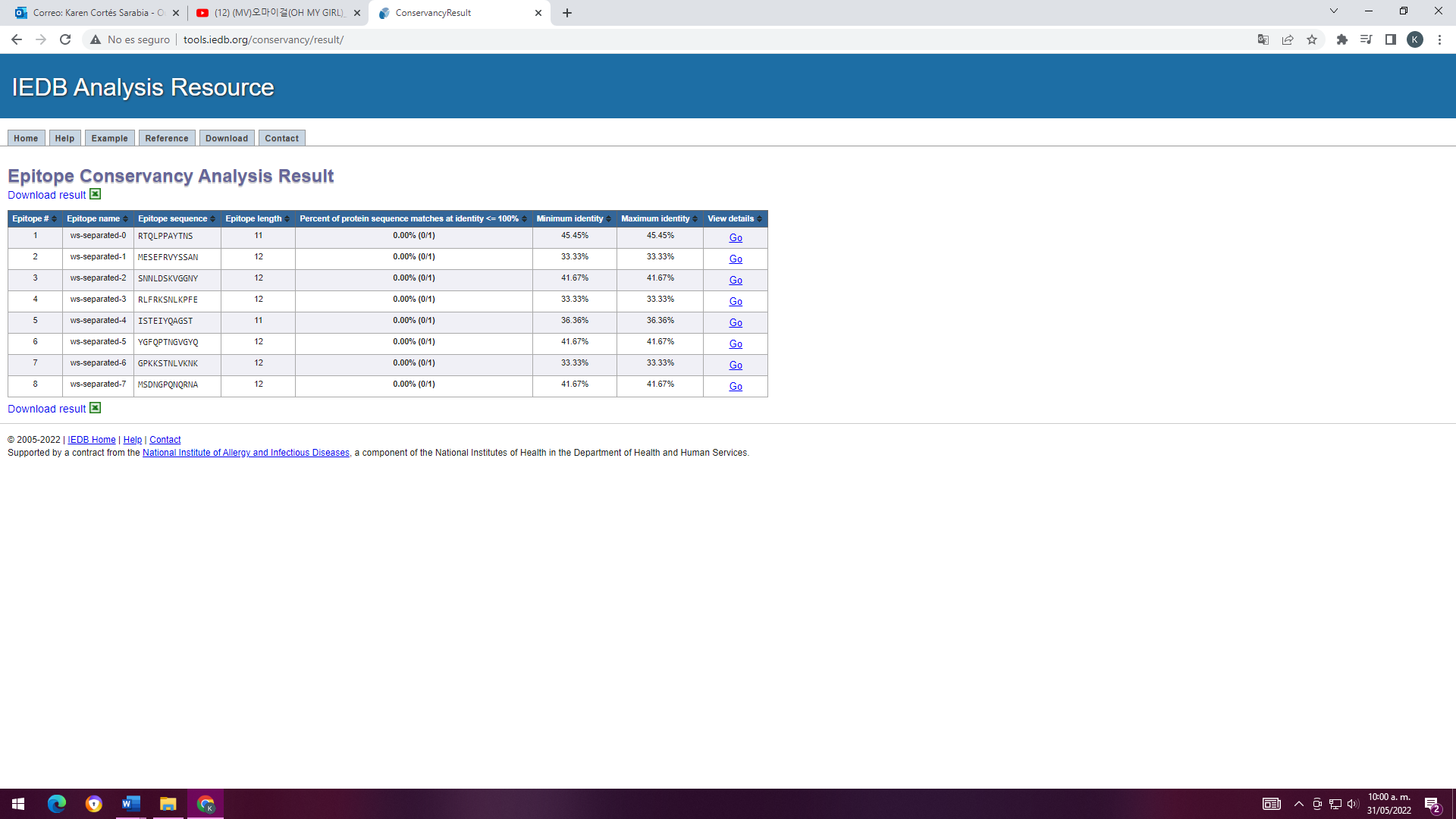


**Porcine coronavirus HKU15 (Access number: AFD29187.1)**


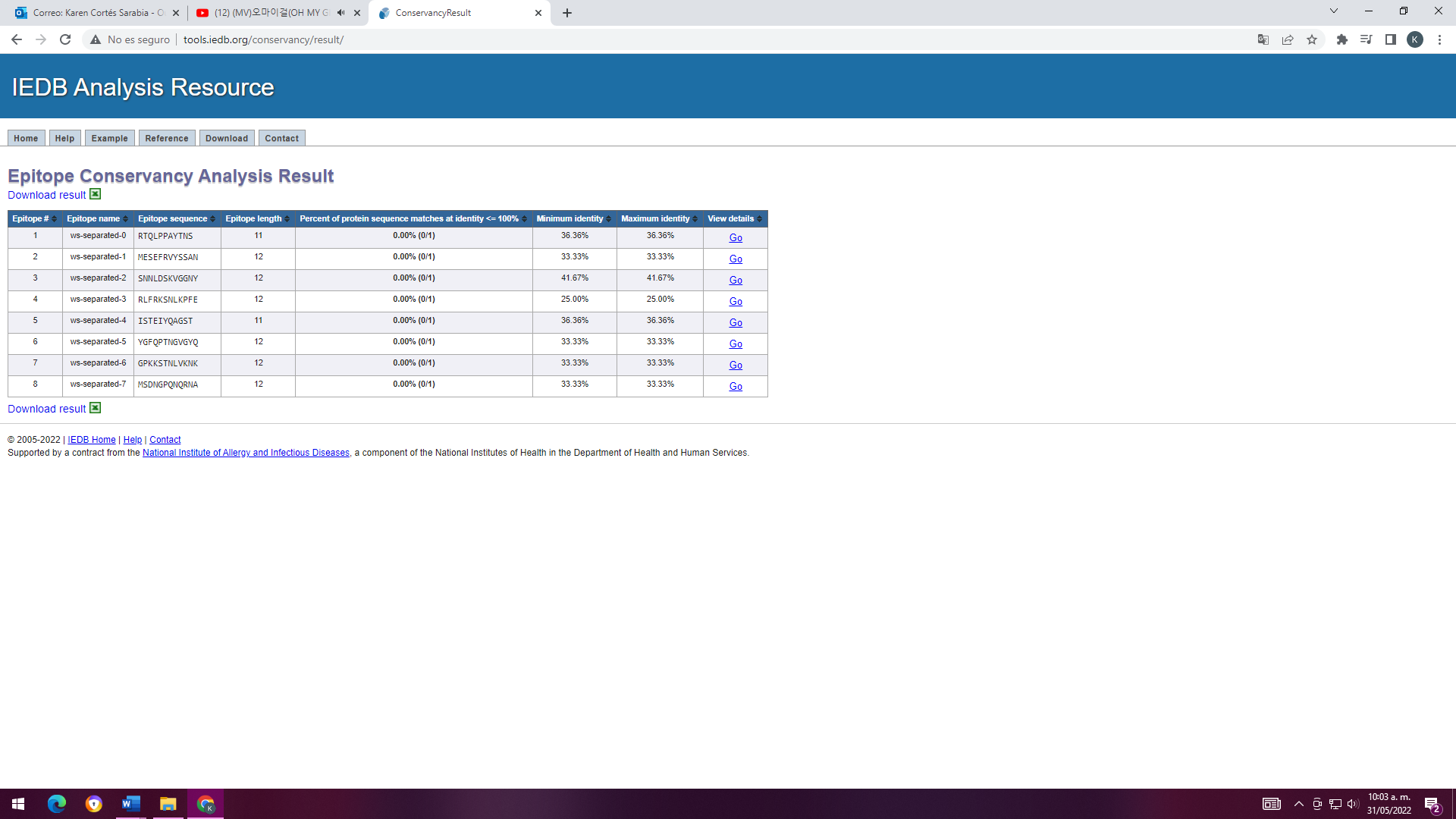


**Munia coronavirus HKU13-3514 (Access number: YP_002308506.1)**


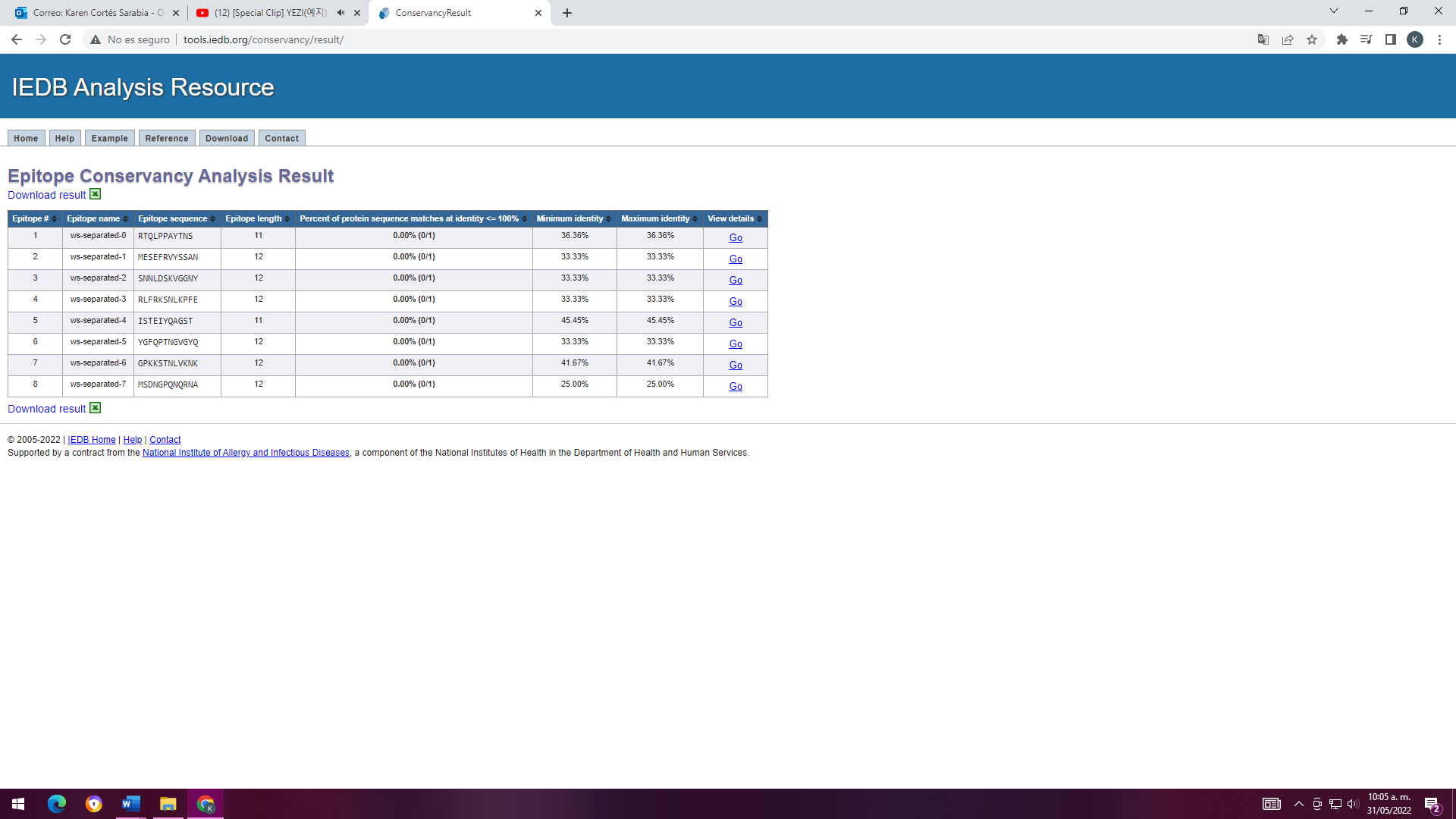


**Bat coronavirus RaTG13 (Access number: QHR63300.2)**


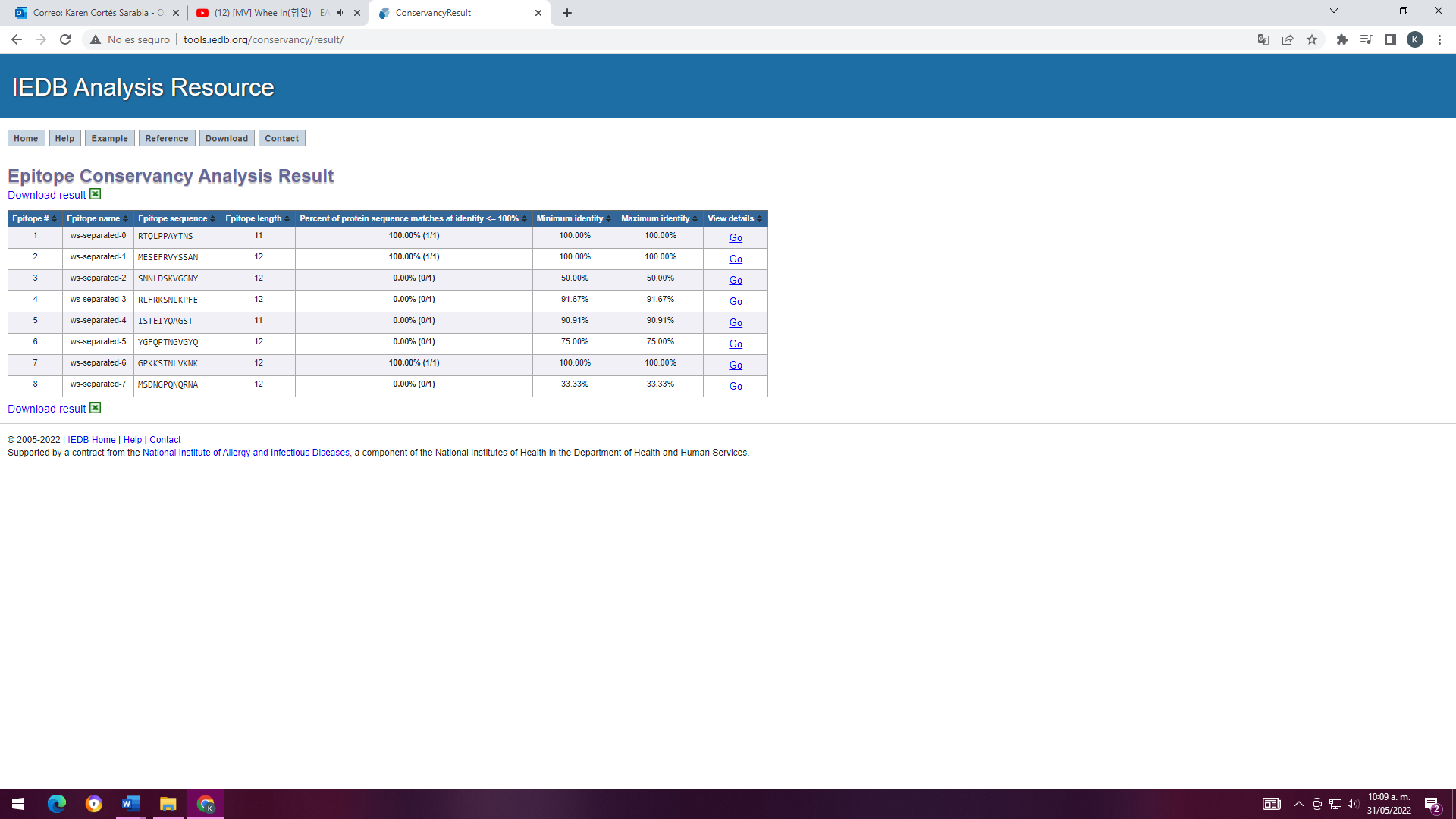


**Bat coronavirus (Access number: QPD89843.1)**


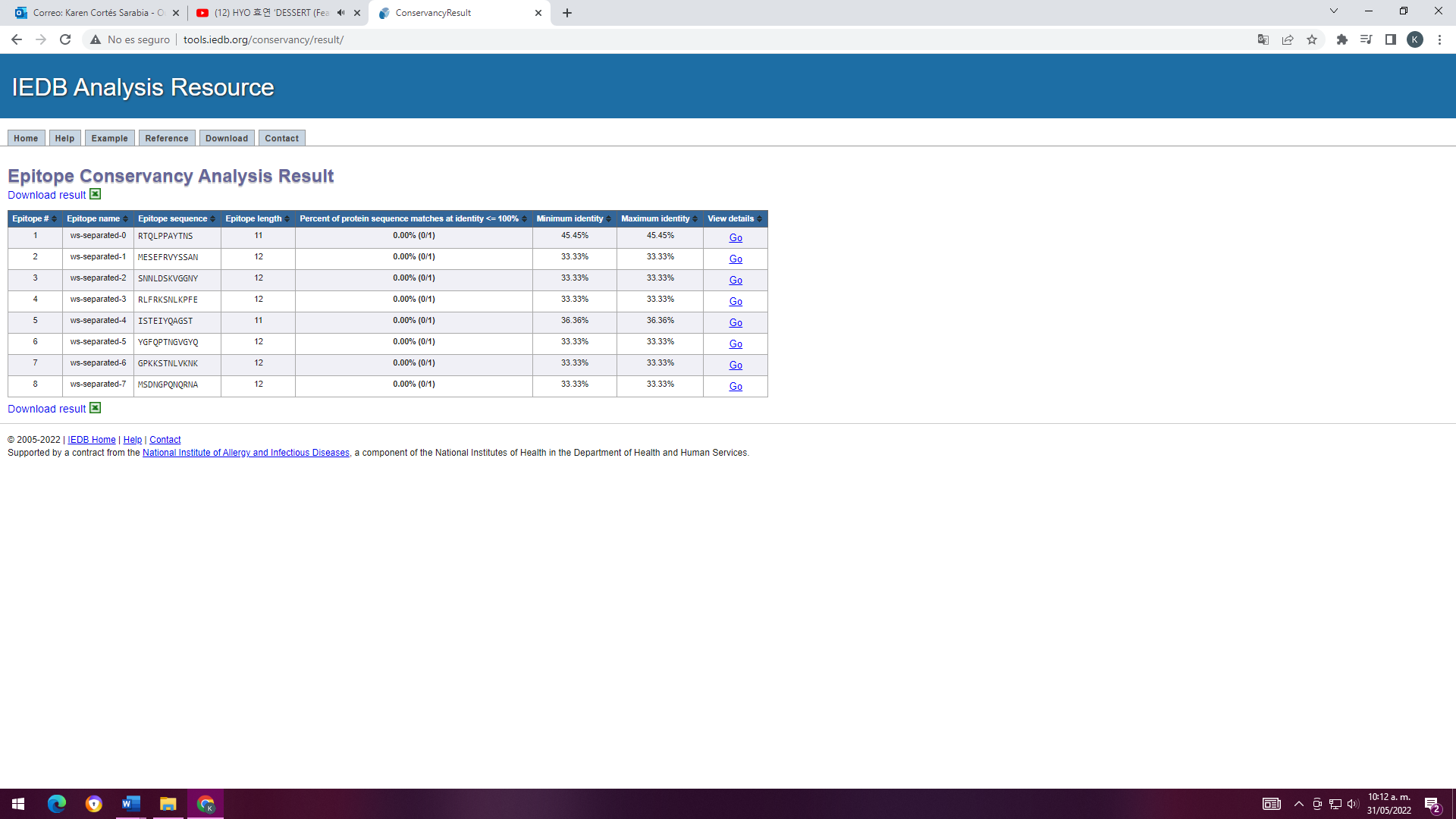


**Bovine coronavirus (Access number: CCE89341.1)**


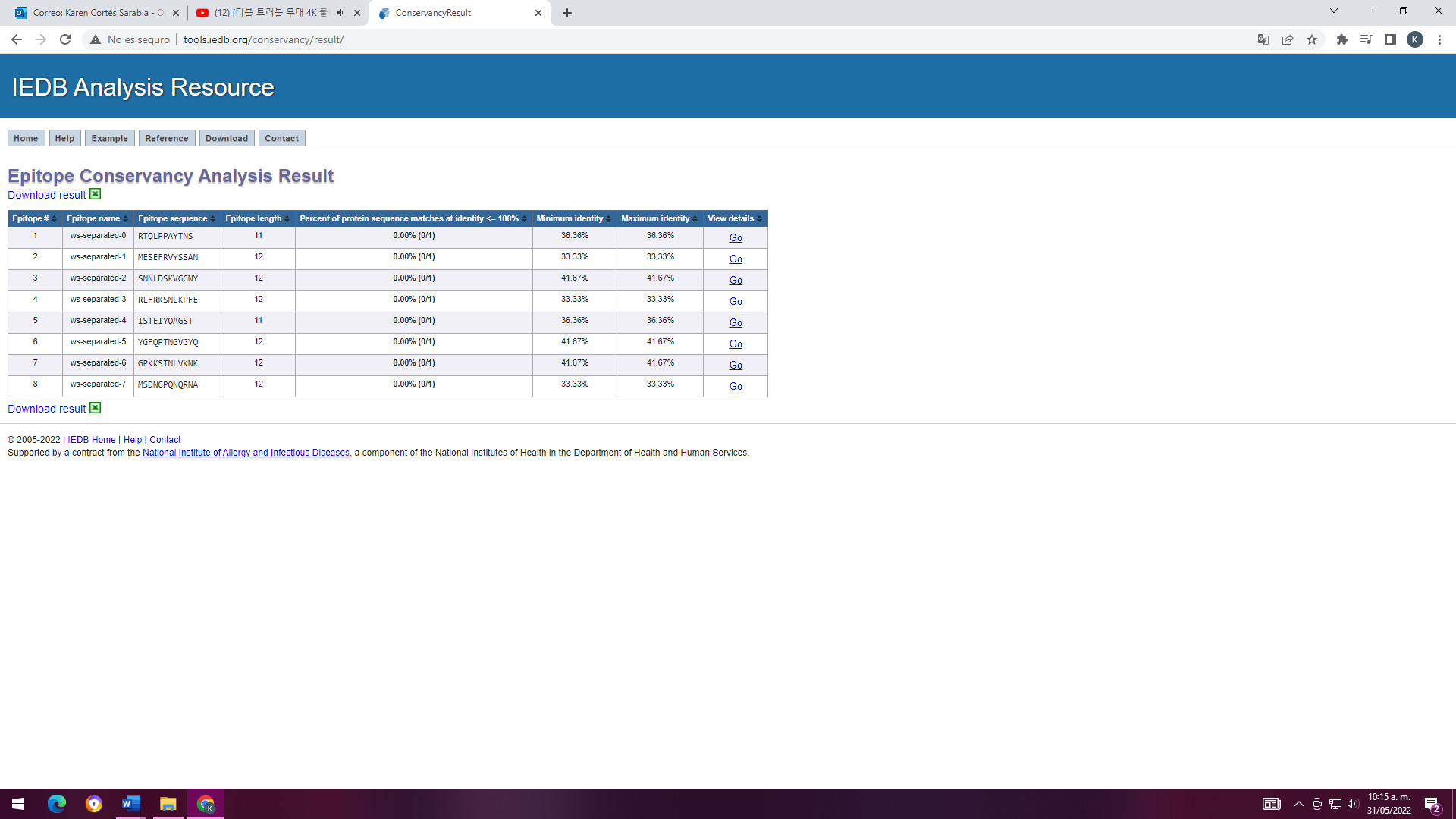


**Avian coronavirus (Access number: QIM61640.1)**


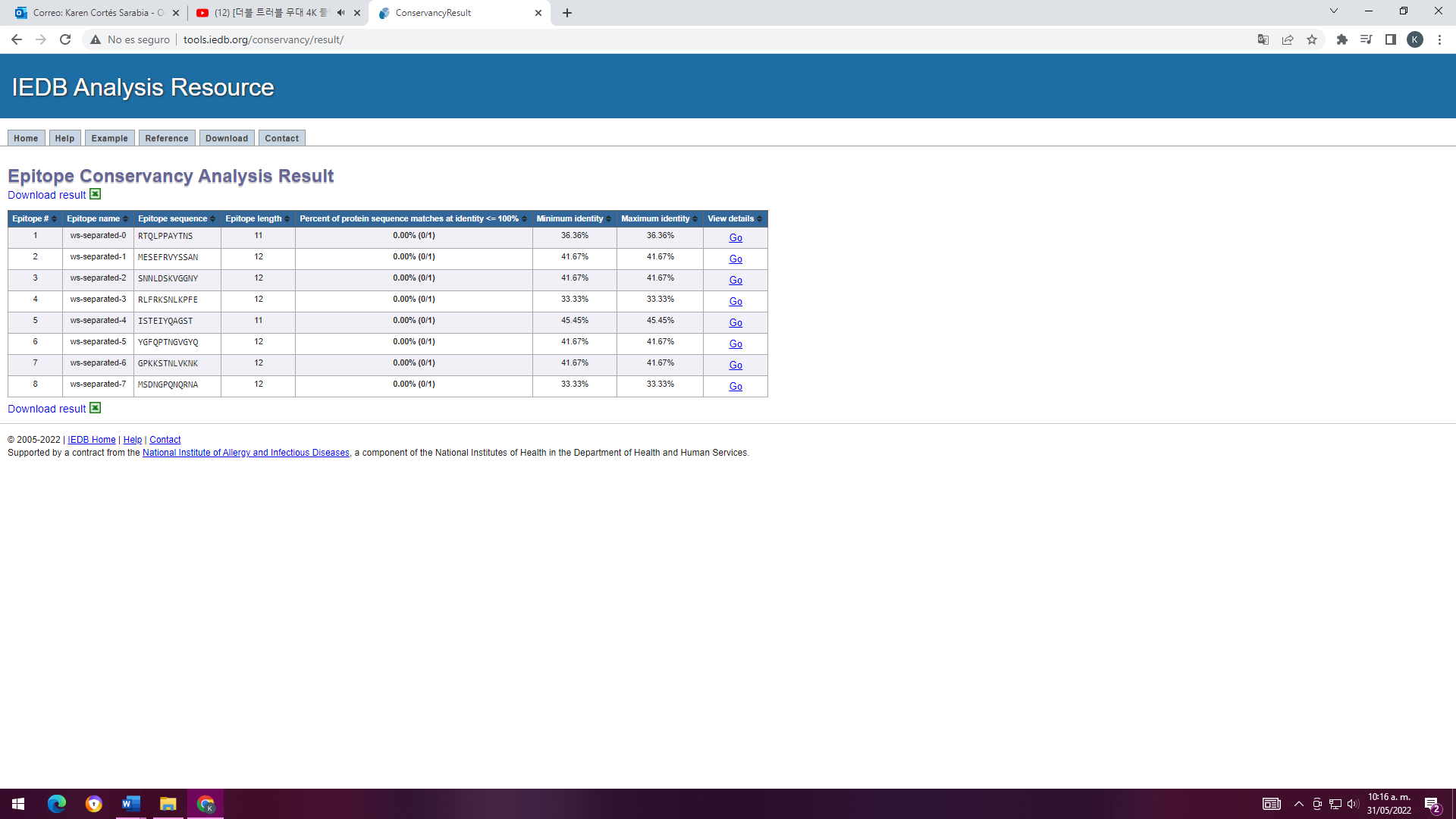

Supplement: Supplementary file 4 — Supplementary Information 4. [file 41598_2022_18517_MOESM4_ESM.docx]
