# Supplementary material for: Utility of in silico-identified-peptides in spike-S1 domain and nucleocapsid of SARS-CoV-2 for antibody detection in COVID-19 patients and antibody production
Source: Sci Rep. 2022 Sep 5;12:15057. doi: 10.1038/s41598-022-18517-w (PMC9442563; doi:10.1038/s41598-022-18517-w)

**Supplementary material 9.- Crude data from Mass spectrometry and HPLC.**

**Source:** Pepmic

**NT-1**


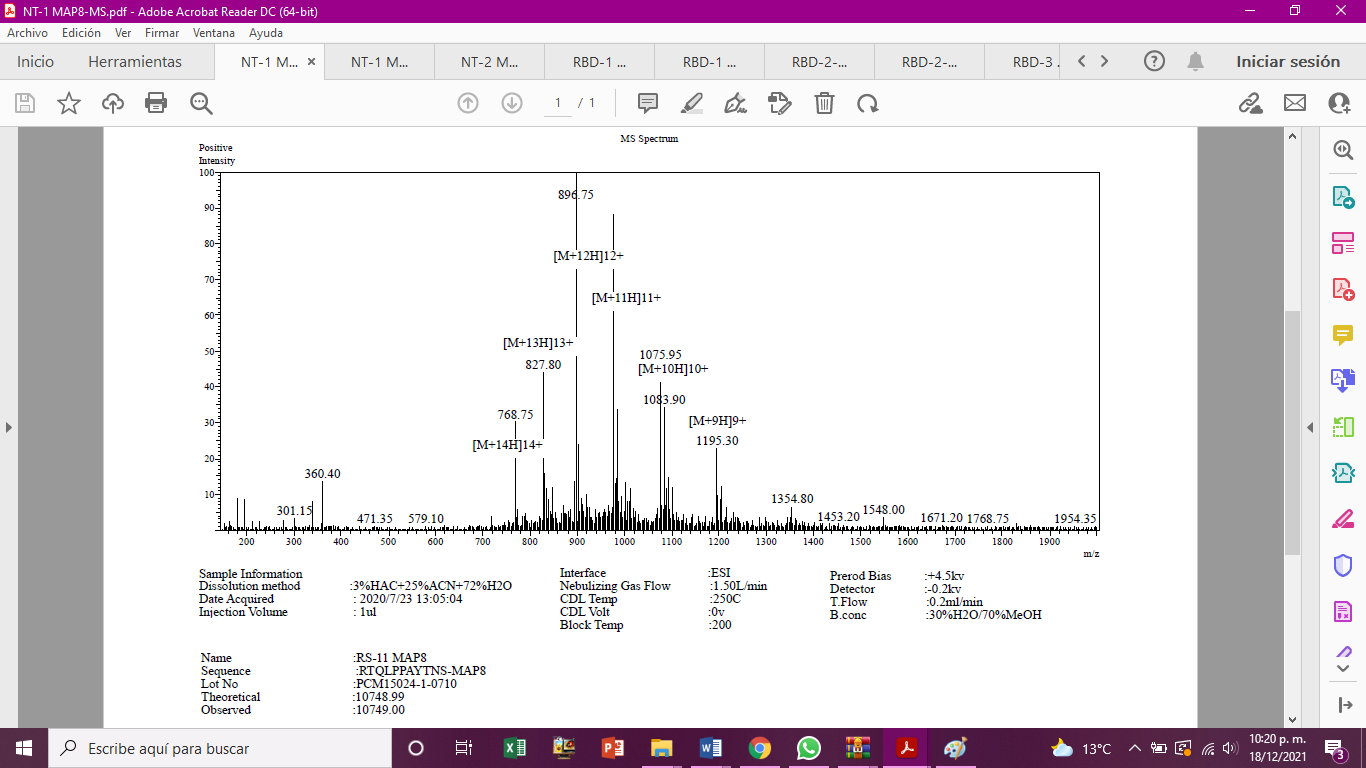


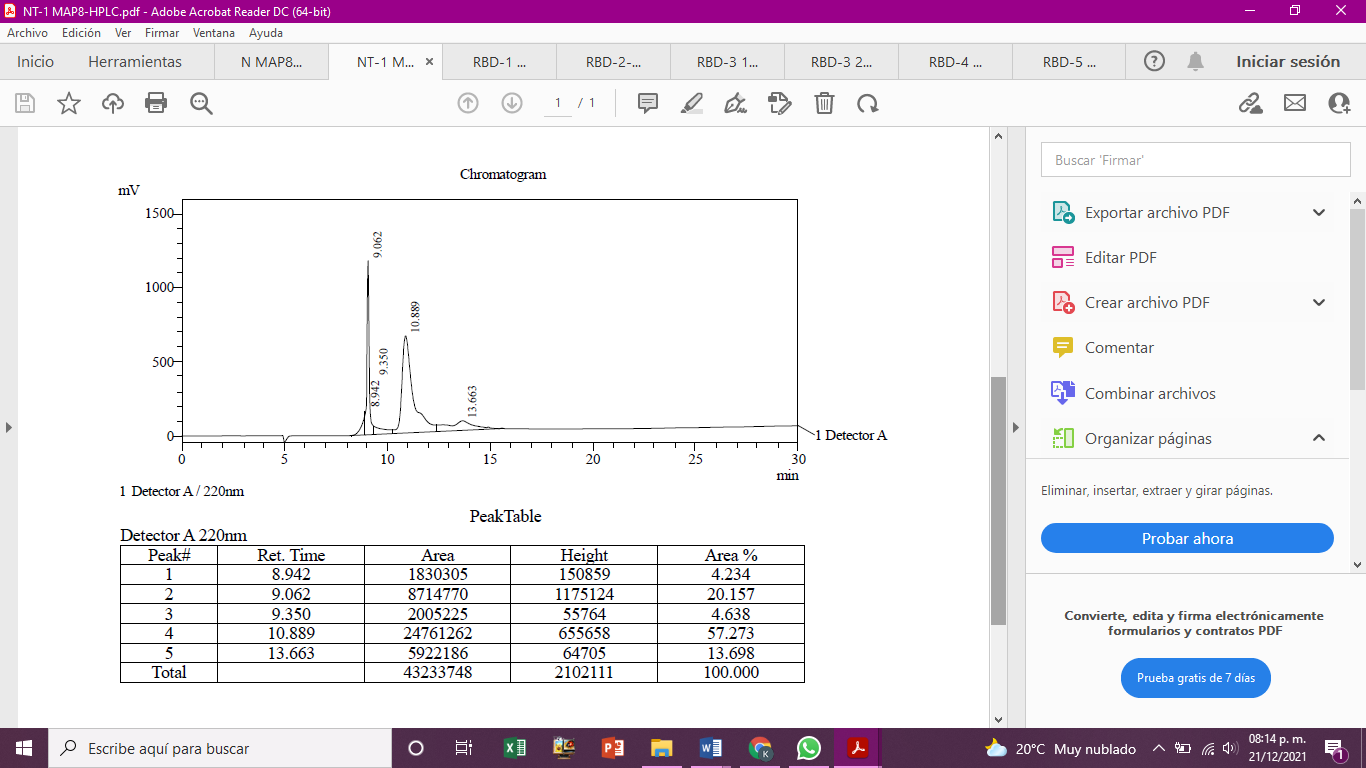


**NT-2**


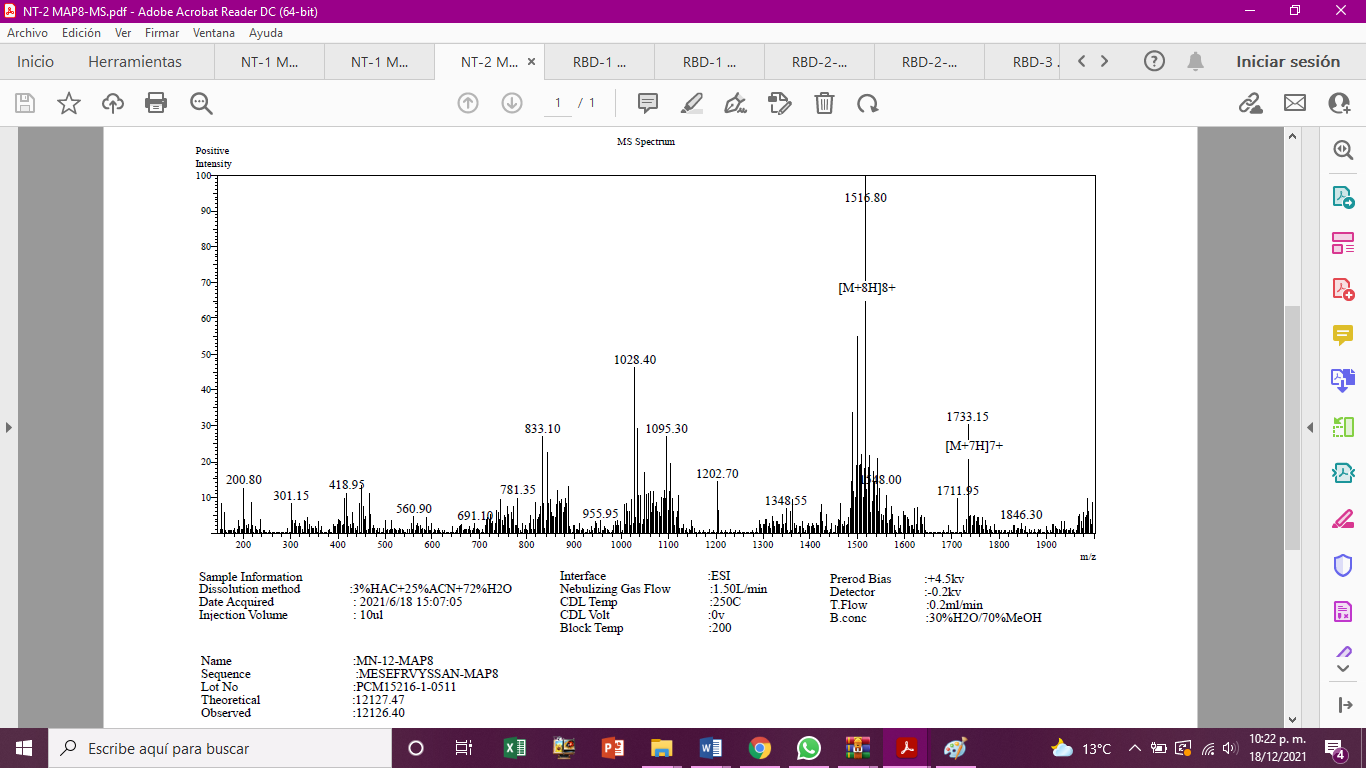


**RBD1**


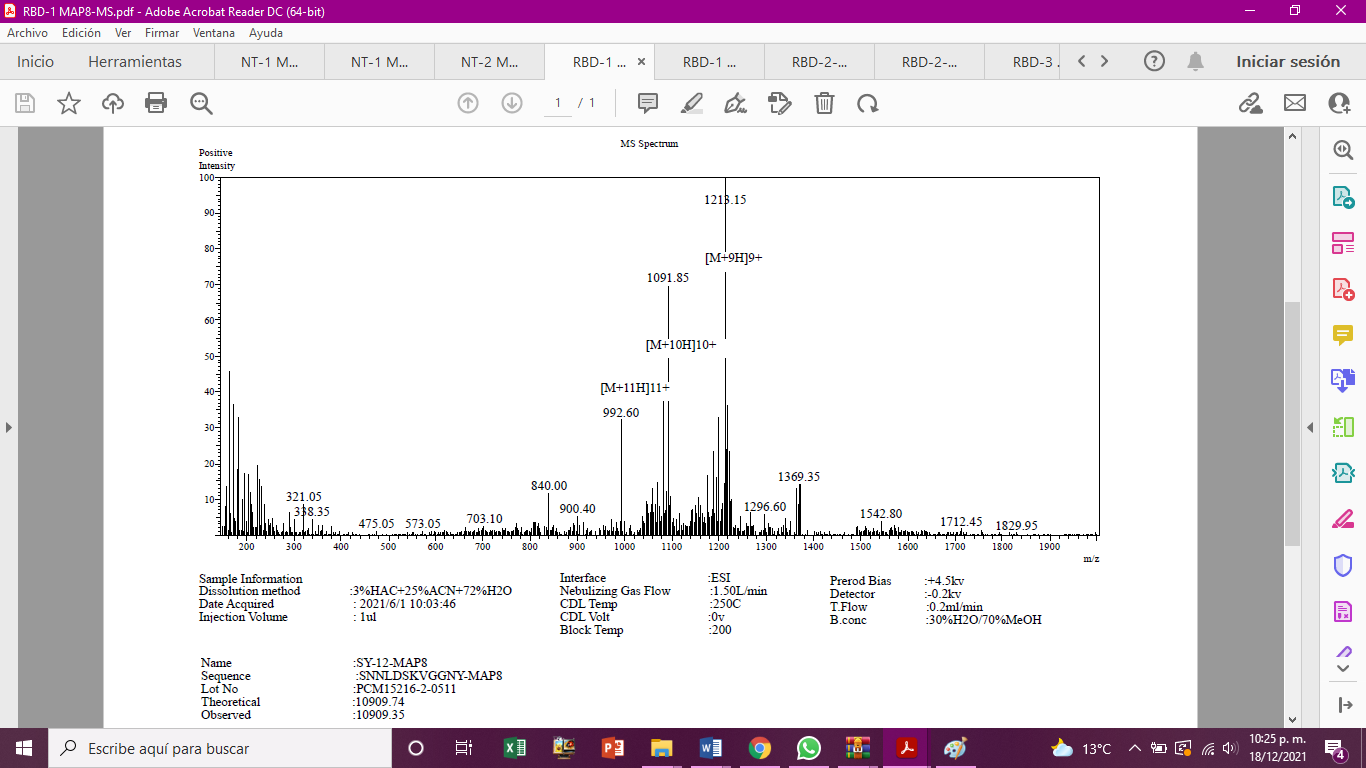


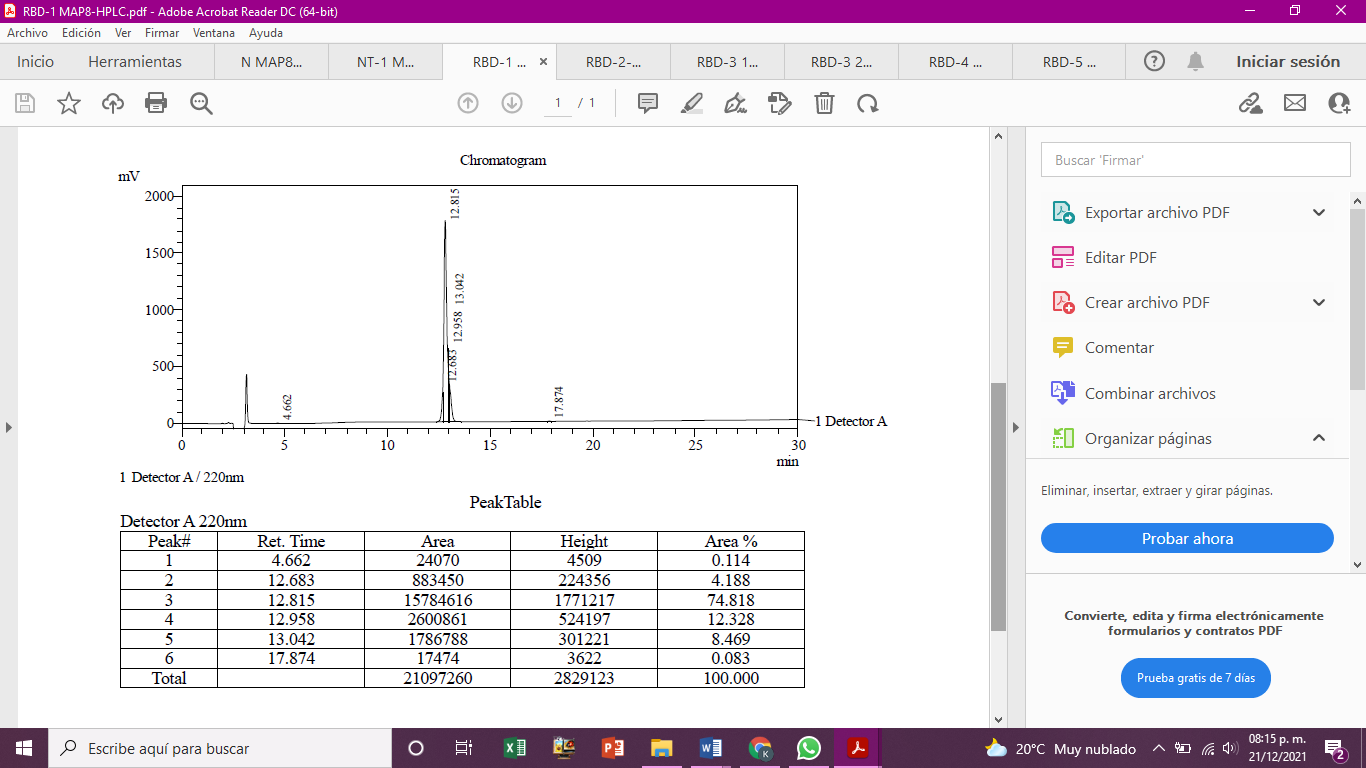


**RBD2**


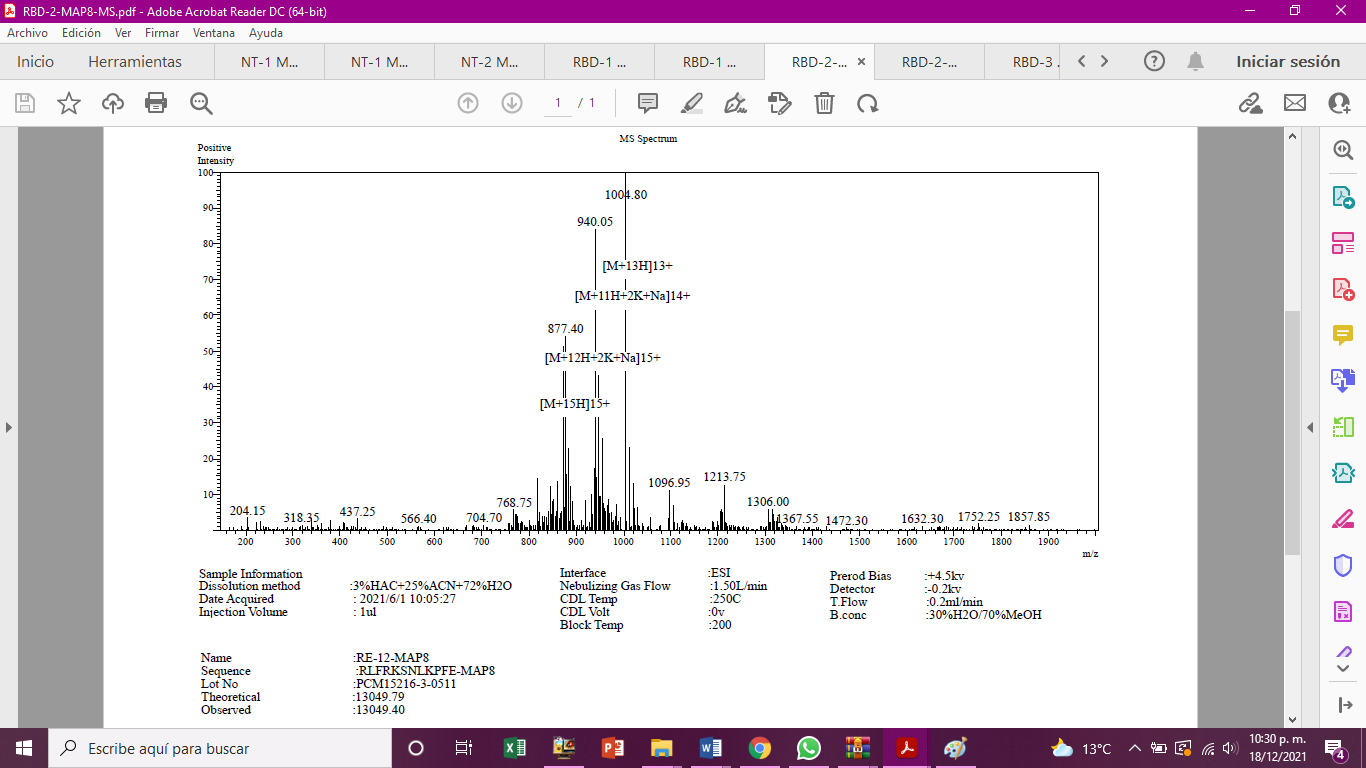


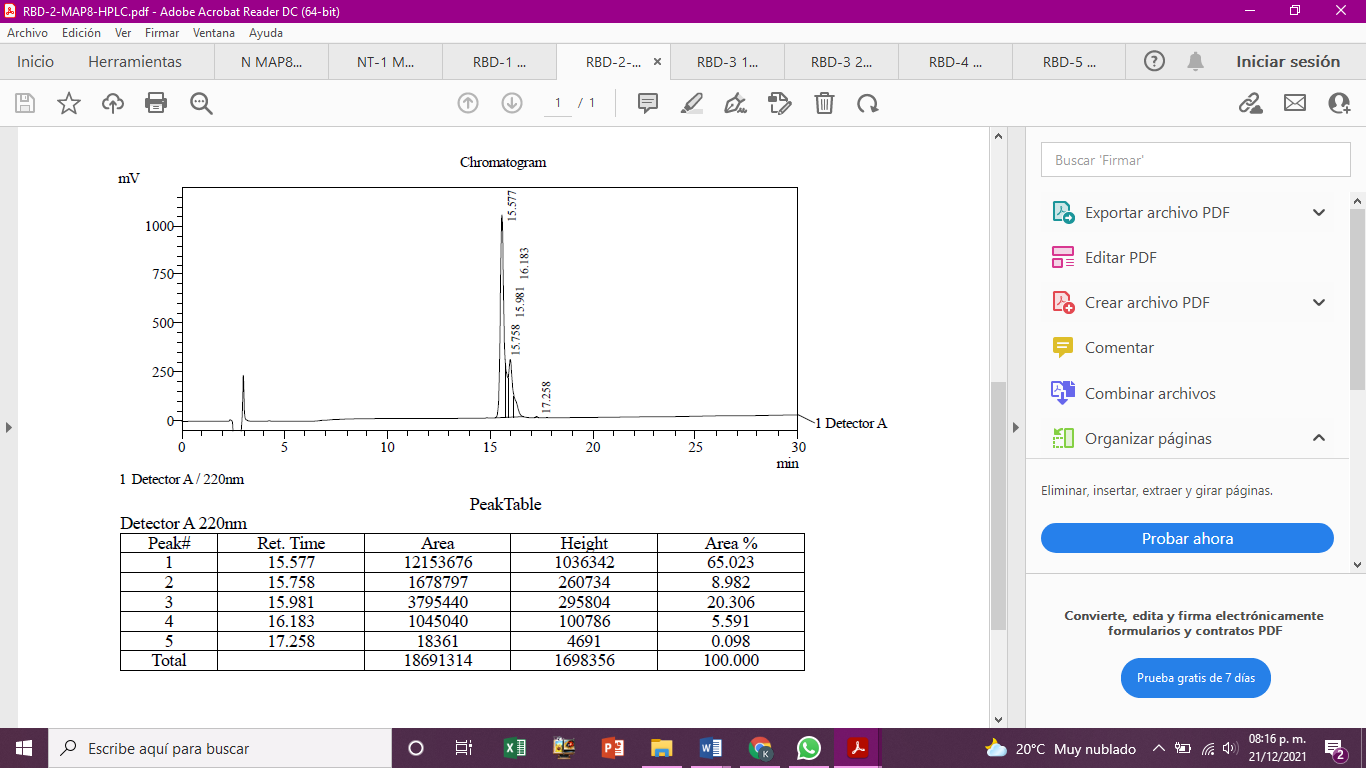


**RBD3**


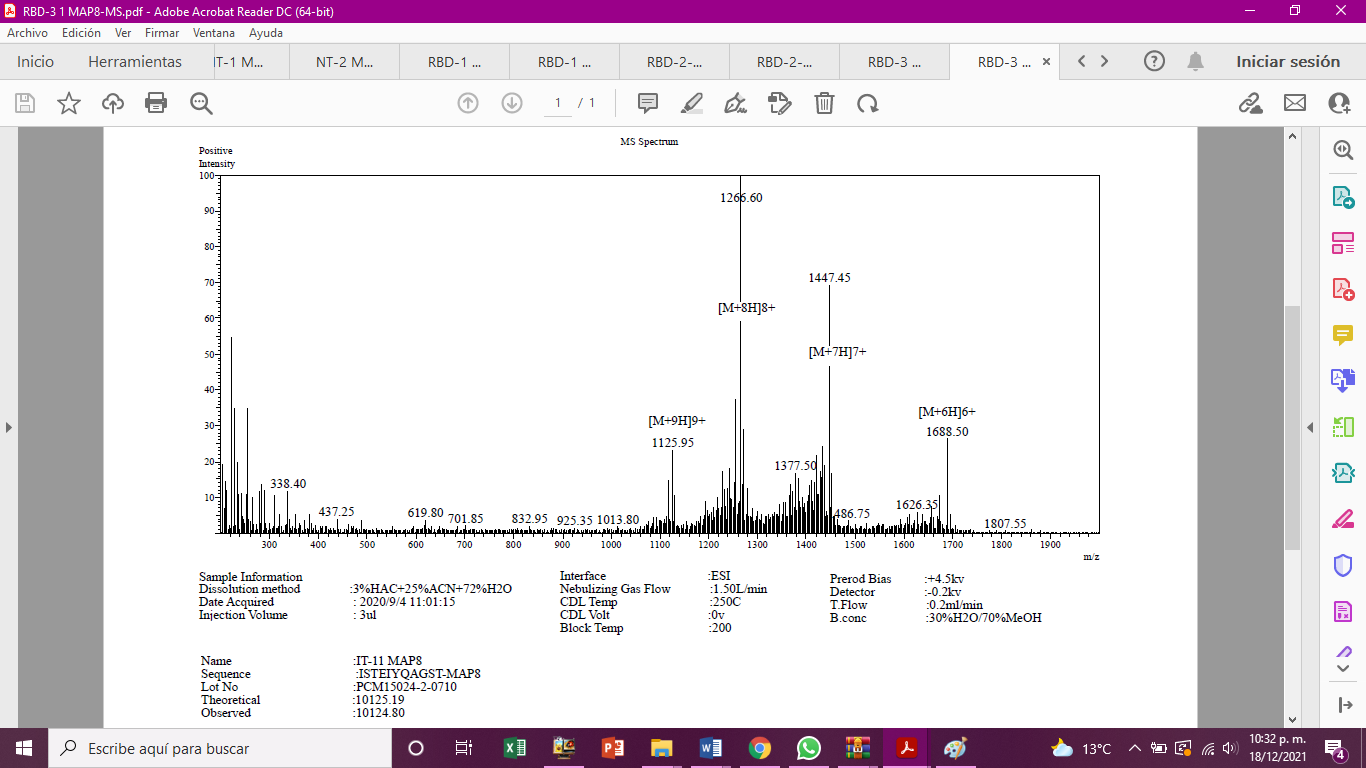


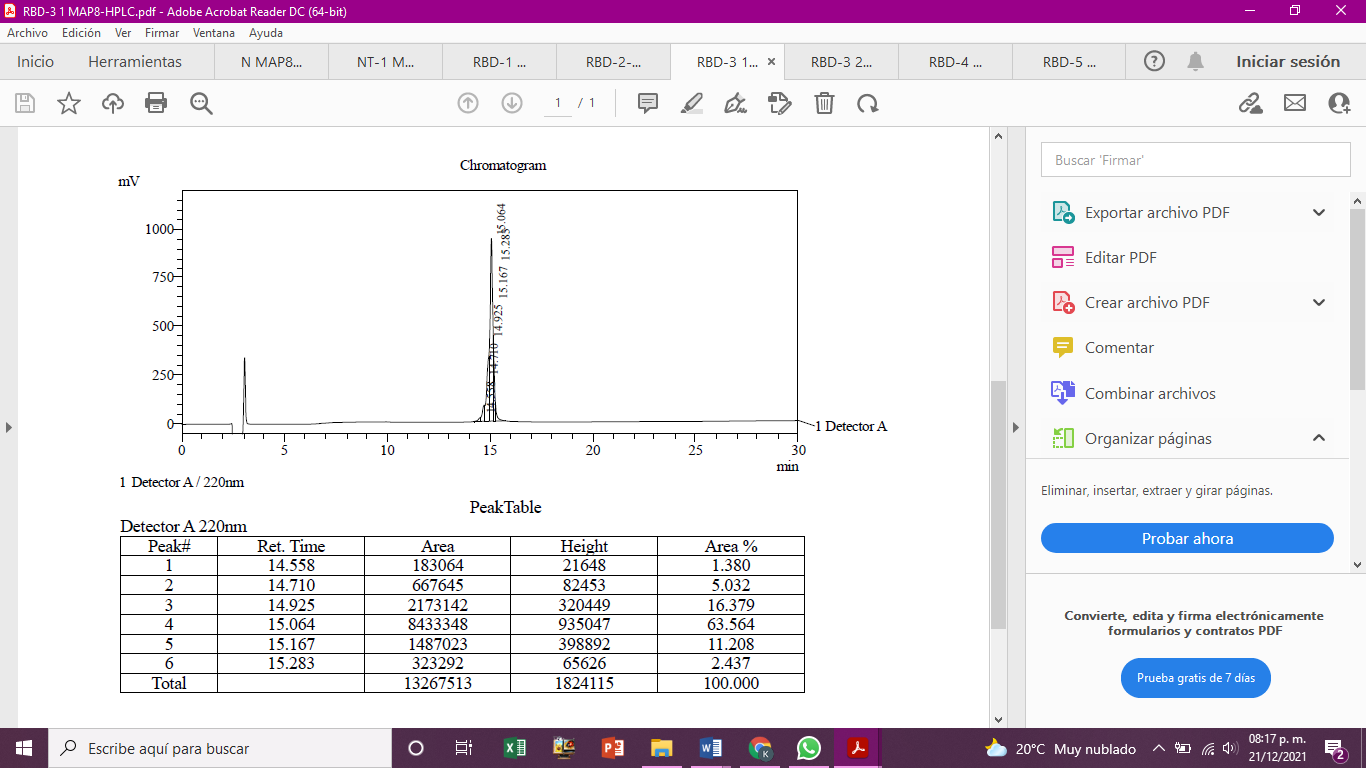


**RBD4**
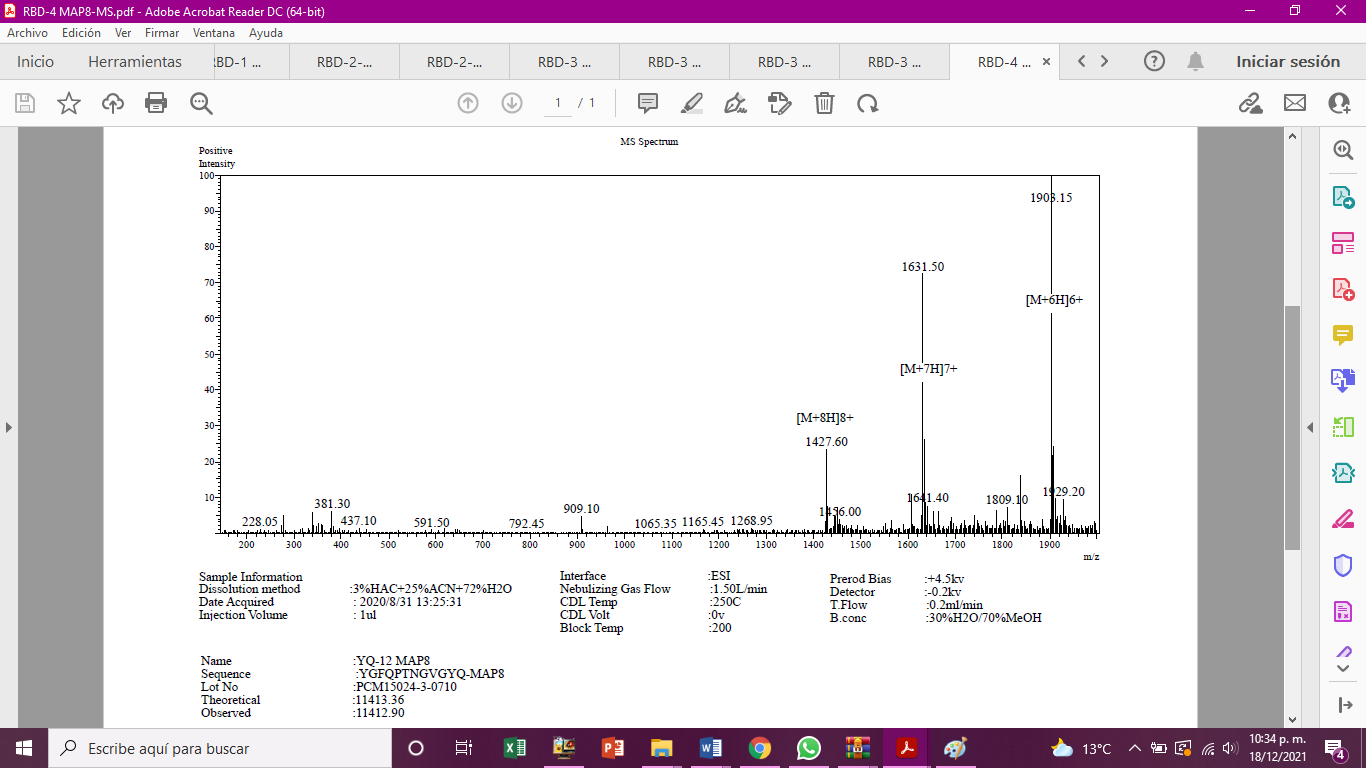


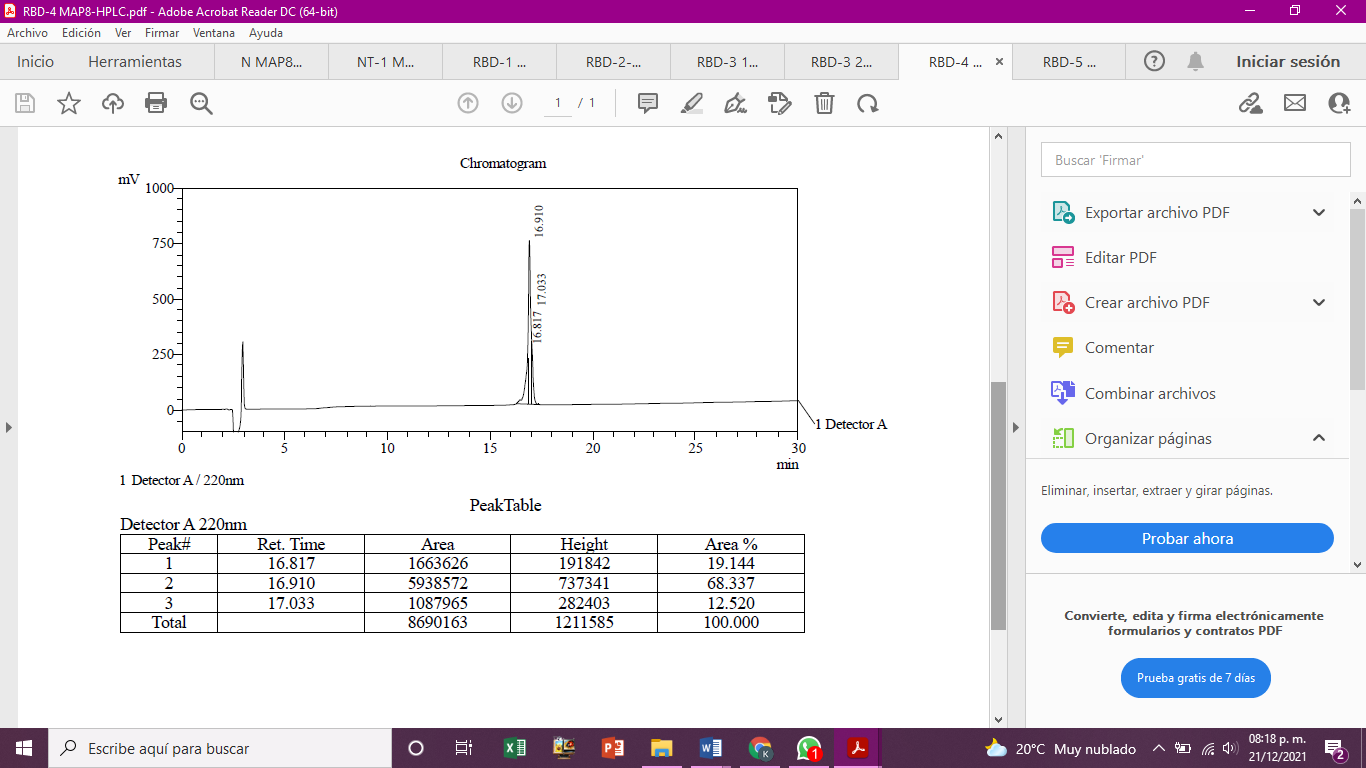


**RBD5**


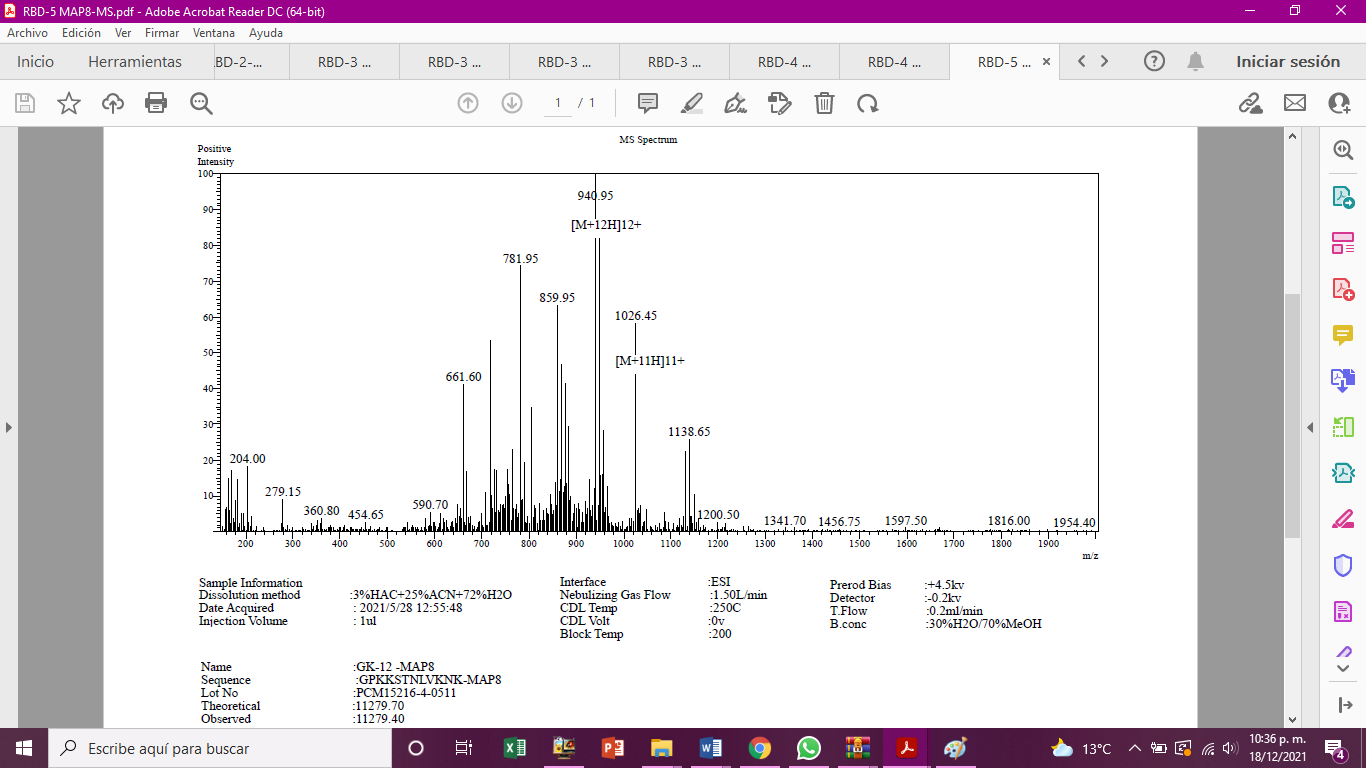


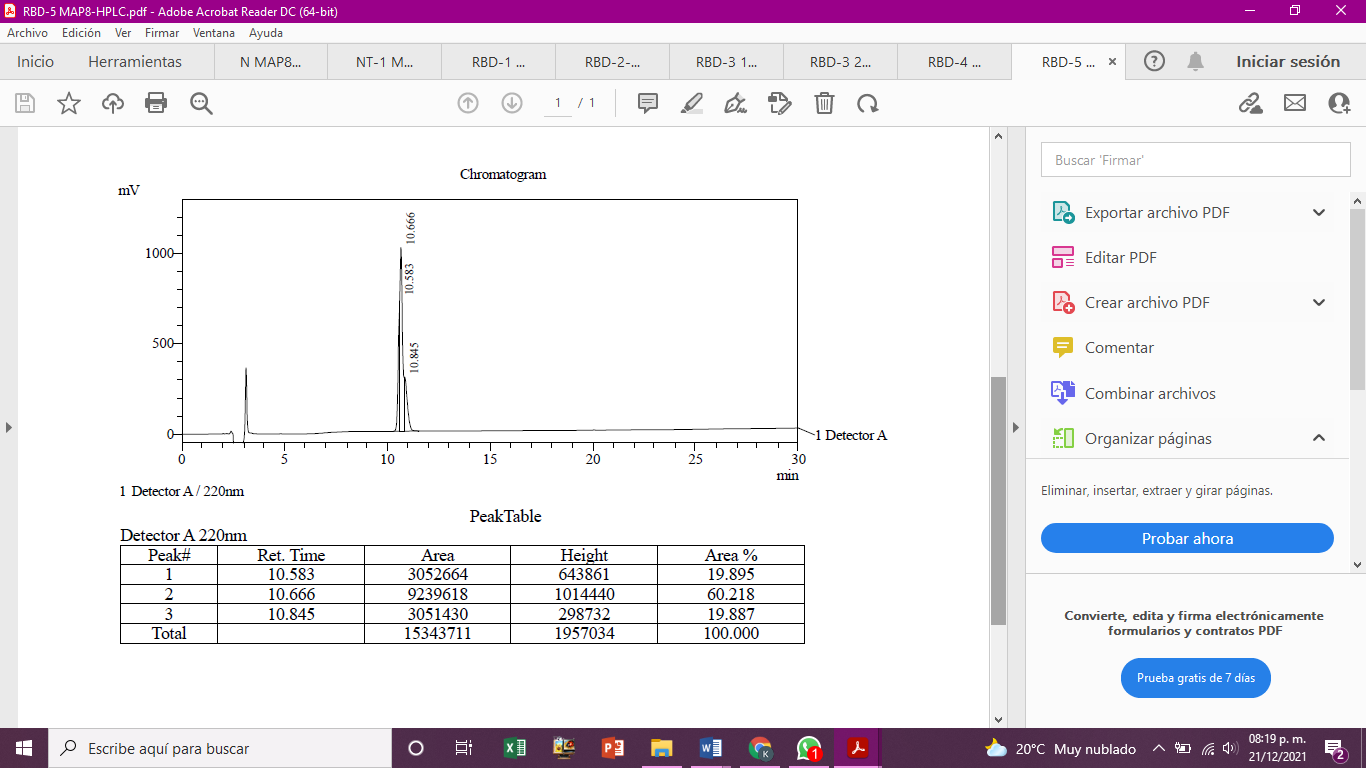


**N**
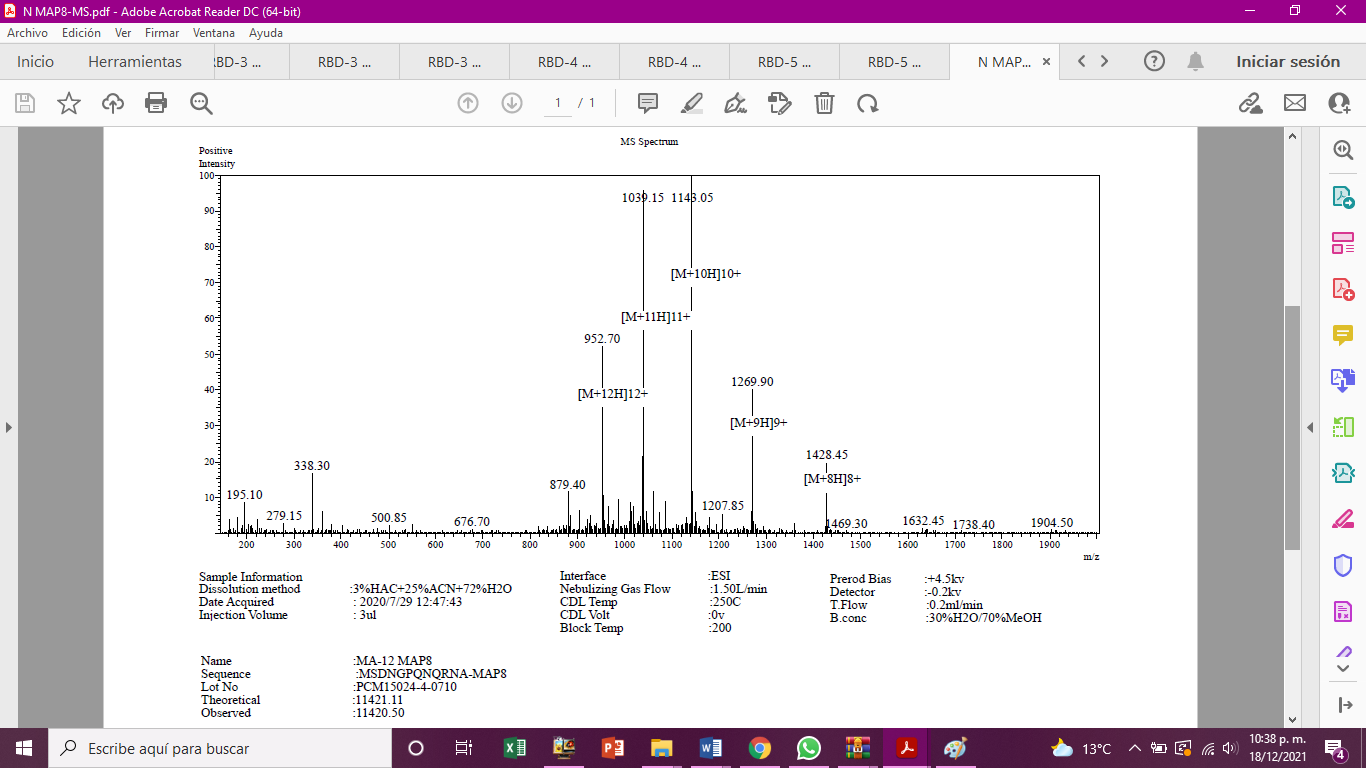


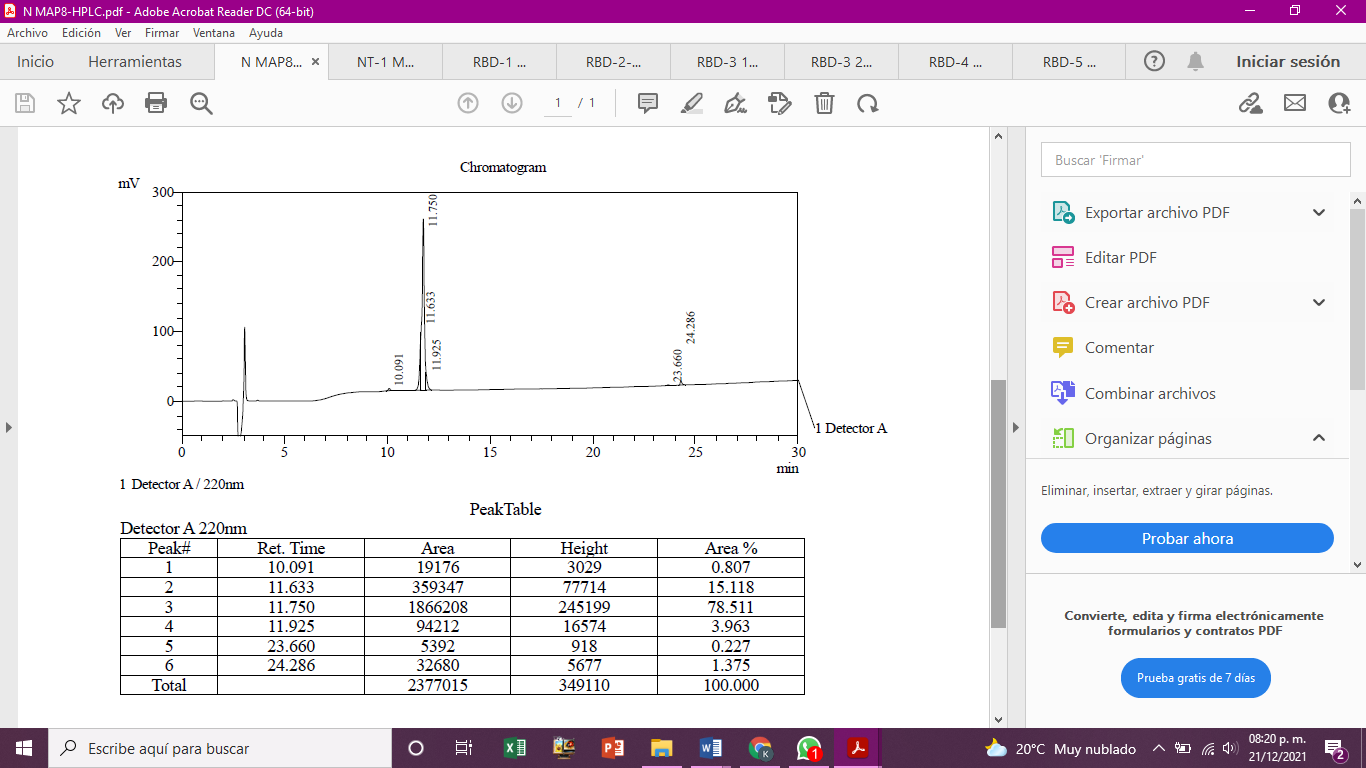

Supplement: Supplementary file 5 — Supplementary Information 5. [file 41598_2022_18517_MOESM5_ESM.docx]
